# Stable *CDK12* Knock-Out Ovarian Cancer Cells Do Not Show Increased Sensitivity to Cisplatin and PARP Inhibitor Treatment

**DOI:** 10.3389/fonc.2022.903536

**Published:** 2022-07-13

**Authors:** Rosaria Chilà, Michela Chiappa, Federica Guffanti, Nicolò Panini, Donatella Conconi, Andrea Rinaldi, Luciano Cascione, Francesco Bertoni, Maddalena Fratelli, Giovanna Damia

**Affiliations:** ^1^ Laboratory of Experimental Oncology, Department of Oncology, Istituto di Ricerche Farmacologiche Mario Negri Istituito di Ricovero e Cura a Carattere Scientifico (IRCCS), Milan, Italy; ^2^ Laboratory of Cancer Pharmacology, Department of Oncology, Istituto di Ricerche Farmacologiche Mario Negri IRCCS, Milan, Italy; ^3^ School of Medicine and Surgery, University of Milano-Bicocca, Monza, Italy; ^4^ Institute of Oncology Research, Faculty of Biomedical Sciences, USI, Bellinzona, Switzerland; ^5^ SIB Swiss Institute of Bioinformatics, Lausanne, Switzerland; ^6^ Oncology Institute of Southern Switzerland (IOSI), Bellinzona, Switzerland; ^7^ Department of Biochemistry, Istituto di Ricerche Farmacologiche Mario Negri IRCCS, Milan, Italy

**Keywords:** CDK12, ovarian carcinoma, PARP inhibitor, cisplatin, DNA damage

## Abstract

Cyclin-dependent kinase 12 (CDK12) is a serine/threonine kinase involved in the regulation of RNA polymerase II and in the transcription of a subset of genes involved in the DNA damage response. *CDK12* is one of the most mutated genes in ovarian carcinoma. These mutations result in loss-of-function and can predict the responses to PARP1/2 inhibitor and platinum. To investigate the role of CDK12 in ovarian cancer, CRISPR/Cas9 technology was used to generate a stable *CDK12* knockout (KO) clone in A2780 ovarian carcinoma cells. This is the first report on a *CDK12* null cell line. The clone had slower cell growth and was less clonogenic than parental cells. These data were confirmed *in vivo*, where *CDK12* KO transplanted cells had a much longer time lag and slightly slower growth rate than CDK12-expressing cells. The slower growth was associated with a higher basal level of apoptosis, but there were no differences in the basal level of autophagy and senescence. While cell cycle distribution was similar in parental and knockout cells, there was a doubling in DNA content, with an almost double modal number of chromosomes in the *CDK12* KO clone which, however did not display any increase in γH2AX, a marker of DNA damage. We found partial down-regulation of the expression of DNA repair genes at the mRNA level and, among the down-regulated genes, an enrichment in the G2/M checkpoint genes. Although the biological features of *CDK12* KO cells are compatible with the function of CDK12, contrary to some reports, we could not find any difference in the sensitivity to cisplatin and olaparib between wild-type and *CDK12* KO cells.

## Introduction

Cyclin-dependent kinase 12 (CDK12) is a serine/threonine kinase involved in the regulation of RNA polymerase II and mRNA processing (1–3). CDK12 is important for the maintenance of genomic stability as it regulates the transcription of a subset of genes involved in the DNA damage response (DDR) (4, 5). In murine embryonic cells, CDK12 sustains self-renewal and its knock-down reduces the expression of self-renewal genes and increases the expression of differentiation markers (6). *CDK12* knockout (KO) mice are not viable, as *CDK12* deletion is fatal during the peri-implantation stage, when *CDK12*-/- blastocysts fail to undergo inner cell mass outgrowth and do not survive *in vitro* due to the induction of apoptosis (5).

CDK12 mutations and amplifications have been reported in different tumor types (7, 8). Not only it is one of the most mutated genes in ovarian carcinoma (9), but its mutations have been correlated with alterations of its catalytic activity, leading to genomic instability, downregulation of genes in the homologous recombination (HR) repair pathway (10, 11) and increased sensitivity to platinum agents and PARP inhibitors (PARPi) (12, 13). HR genes have multiple intronic to poly-adenylation sites that are susceptible to CDK12 inhibition, leading to decreases in their expression (14, 15). In addition, CDK12 inhibition induces an RNA Pol II elongation defect with the subsequent use of proximal poly(A) sites that leads to premature cleavage and polyadenylation especially in DDR gene, known to have longer lengths (16).

High-grade serous ovarian carcinomas (HGSOC) with mutated *CDK12* present a unique signature of genomic instability characterized by tens to hundreds of large tandem duplications scattered throughout the genome, probably due to defects in DNA repair (17). In breast cancer *CDK12* amplification co-occurs with *ERBB2* amplification (18, 19) and its over-expression has been associated with aggressive disease (18). The absence of CDK12 protein, however, correlates with the triple negative phenotype and with reduced expression of some DDR proteins, but not with lower DDR mRNA levels (20). *CDK12* mutations in primary and castration-resistant prostate cancer have also been reported, mutually exclusive with other mutations in DNA repair genes (21–23).

With the aim of understanding the role of CDK12 in tumor growth and response to therapy better, we used CRISPR/Cas9 technology to generate a *CDK12* knock-out system in A2780 ovarian cancer cell lines.

## Materials and Methods

### *CDK12* KO Clone

The ovarian carcinoma cell line A2780 was obtained from the American Type Culture Collection (ATCC) and was authenticated by the authors within the last six months. Cell lines were maintained in RPMI culture medium supplemented with 10% FBS and 1% glutamine, at 37°C with 5% CO_2_. A2780 cells were initially transfected with CRISPR/Cas9 plasmid directed against exon 1 of CDK12 (U6gRNA-Cas9-2A-GFP, Sigma-Aldrich). After 48 hours, cells were seeded at very low density in order to isolate, expand and analyze single clones by western blot and DNA sequencing. We obtained only a CDK12 heterozygous KO clone, out of about 150 clones screened. On this clone, we ran a second round of transfection and selection and obtained an A2780 homozygous KO clone (A2780 KO).

### DNA Sequencing

Total DNA was purified from cells (Maxwell Total DNA Purification Kit, Promega) and the selected *CDK12* locus was amplified with a polymerase chain reaction (PCR) (**Supplementary Table 1**). Amplified DNAs were separated through electrophoresis, and Sanger-sequenced.

### Cell Growth

Growth curves were obtained seeding the cells at 15000 cells/mL in six-well plates and counting them at different time points with a cell counter (Multisizer 3, Beckman Coulter). For clonogenic assay in six-well plates, cells were seeded at 125 cells/mL; colonies were left to grow for about ten days then stained with Gram’s Crystal Violet solution (Merck). For the limiting dilution assay, cells were seeded at 0.5 cells/well in 96-well plates and colonies were left to grow for about 30 days.

### Flow Cytometry Analysis of DNA Content

Exponentially growing cells were washed twice in ice-cold PBS, fixed in ice-cold 70% ethanol, washed in PBS, re-suspended in 2 mL of a solution containing 25 μg/mL of propidium iodide in PBS and 25 μL of RNase 1 mg/mL in water, and stained overnight at 4°C in the dark. We used the FACS Calibur (Becton Dickinson) for cell cycle analysis.

### Chromosome Analysis

Exponentially growing A2780, and A2780 *CDK12* KO cells were treated for 6 hours with colcemid (Roche) 0.2 μg/mL in order to block them in metaphase, then treated with hypotonic solution (75 mM KCl) and fixed with 3:1 (vol/vol) methanol:acetic acid. We karyotyped 19 metaphases for A2780 and fifteen for A2780 *CDK12* KO cells lines. Chromosome preparations were analyzed by QFQ banding (Q-Bands by Fluorescence using Quinacrine) according to routine procedures and the karyotype was described using the International System for Chromosome Nomenclature 2016.

### Cell Death Analysis

To investigate apoptosis in the cells, we used a caspase 3/7 luminescence-based assay (Promega) and the β-galactosidase staining assay (Cell Signaling Technology) for the detection of senescent cells.

### Western Blotting

Proteins were extracted and processed as already described (24). The primary antibodies used are listed in **Supplementary Table 2**. All the protein blots for western analysis have been cropped before antibody hybridization to be able to detect in the same filter different proteins.

### Real-Time (RT)-PCR

Total RNA from cell lines was purified with the Maxwell 16 Total RNA Purification Kit (Promega) and reverse transcribed with the cDNA Archive Kit (Applied Biosystems). mRNA expression of the genes was detected using Sybr Green assays (Applied Biosystems). Reactions were run in a total volume of 20 μL containing 10 ng of cDNA with Sybr Green and the forward and reverse primers of the gene, in triplicate (**Supplementary Table 1**). All the data were normalized to the levels of the cyclophillin gene and expressed as the -fold increase over the wild type *CDK12* cells.

### Gene Expression

As already described (25), NEBNext Ultra Directional RNA Library Prep Kit for Illumina (New England BioLabs Inc., Ipswich, MA, USA) was used for RNA-seq experiments and the NEBNext Multiplex Oligos for Illumina (New England BioLabs Inc.) for cDNA synthesis. The pre-pool sequencing was done using the NextSeq 500 (Illumina, San Diego, CA, USA) with the NextSeq 500/550 High Output Kit v2.5 (75 cycles; Illumina) and for all the samples we used stranded, single-ended 75bp-long sequencing reads.

### Drugs and Treatments

Cisplatin was purchased from Sigma Aldrich; paclitaxel from ChemieTek; olaparib from LC Laboratories; VE822, MK1775 and KU55933 from Axon Medchem; THZ1 and THZ1 hydrochloride were from Insight Biotechnology Limited; ET-743 from PharmaMar and PF-00477736 from Pfizer. For cytotoxicity experiments, cell lines were seeded in 96-well plates and were treated after 48 hours with different concentrations of the drugs. After 72 hours cell viability was examined with the MTS assay (Promega) and absorbance was acquired using a plate reader (Infinite M200, TECAN). Drug concentrations inhibiting growth in 50% of the cells (IC50s) were calculated for each cell line, with the interpolation method.

### Chemotaxis and Chemo-Invasion Tests

The cell lines were tested for their ability to migrate through a Nucleopore Track-Etch Membrane (pore size 8 μm, Whatman International), coated with Matrigel Basement Membrane Matrix for chemo-invasion, using Boyden chambers. We added 05 to 2 x 10^4^ cells, re-suspended in DMEM culture medium with 0.1% FBS, to each well of the upper chamber for chemotaxis and chemo-invasion tests; DMEM+0.1% FBS (control wells) or 3T3 cell line supernatant (chemo-attractive agent) were added to the lower chamber. The chambers were incubated at 37°C overnight, then the cells from the upper side of the filter were removed and filters were fixed in methanol and stained for nuclear and cytoplasmic detection. Cells that had migrated the lower side of the filters were quantified by brightfield microscopy using a 40× objective, and the average number of cells per field was calculated.

### *In Vivo* Studies

NCr-nu/nu mice (five-week-old females) were from Harlan S.p.a Italy and maintained under standard pathogen-free conditions. The Istituto di Ricerche Farmacologiche Mario Negri IRCCS adheres to national and international laws, regulations, and policies on the maintenance, care and use of laboratory animals, as already reported (26). The *in vivo* experiments were approved by an institutional review board and the Italian Ministry of Health (Authorization no. 705/2016-PR). Exponentially growing A2780 and A2780 *CDK12* KO cells (approximately 7.5 x 10^6^ cells per mouse) were injected subcutaneously in the flank of four mice per group. A Vernier caliper was used to measure tumor diameters, and tumor volumes were calculated following the formula: [(smallest diameter)^2^ x biggest diameter]/2. Tumor and body weights were recorded at different time points from implant and mice were euthanized by carbon dioxide (CO2) overdose when tumors reached 10% of the animal’s body weight.

### Statistical Analyses

Statistical analyses were done with GraphPad Prism software. We used a *t-test* to compare cell growth, colony formation, IC50s of cytotoxic experiments and chemo-attraction and chemo-invasion data between the *CDK12* wild type (WT) and *CDK12* KO cell lines. To compare the activation of apoptosis between *CDK12* WT and *CDK12* KO cells we used two-way ANOVA followed by a Bonferroni test.

RNA-Seq was analyzed as previously described (27). Briefly, after quality control using fastqc and mapping against the human GRCh38 genome build, alignment was done with STAR, reads counted with HTSeq-Count, and differently expressed transcripts defined with the voom/limma R package. Genes were considered up-regulated or down-regulated if the adjusted p-value was <0.05 and log2 (KO vs WT) was respectively >1 or <-1. Genes deregulated in both cell lines were analyzed for enrichment in cancer hallmarks using the web-based tool of the Molecular Signaling Database (MsigDB, http://software.broadinstitute.org/gsea/msigdb), filtering for a false discovery rate (FDR) <0.05.

## Results

### *CDK12* KO Ovarian Cancer Cell Lines

**Figure 1:**
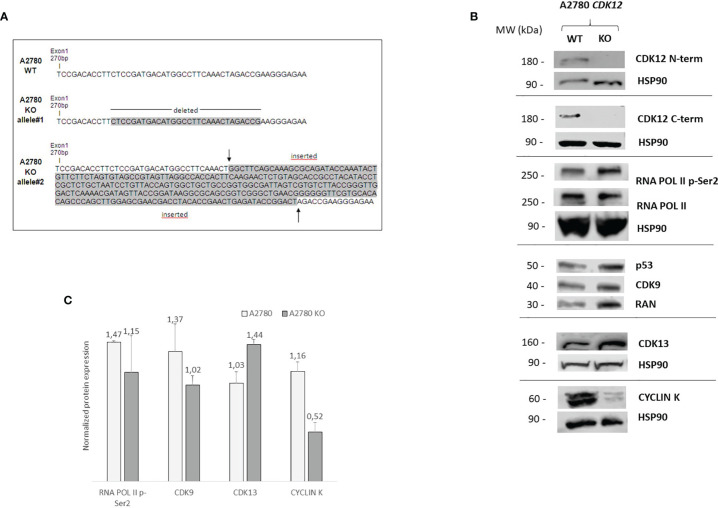
Molecular characterization of A2780 and A2780 *CDK12* KO cells. **(A)** Nucleotide sequences of the loci targeted by CRISPR/Cas9 in exon 1 of *CDK12*, generating *CDK12* KO clones. Upper panel: nucleotide sequences of A2780 *CDK12* WT and KO; shaded nucleotides were deleted and inserted in KO clone allele #1 and #2, respectively. **(B)** Western blot analysis showing *CDK12*, HSP90, RNA pol II p-Ser2, RNA pol II, p53, CDK9, cyclin K, CDK13, RAN protein levels in A2780 and A2780 *CDK12* KO cell lines. All the protein blots for western analysis have been cropped before antibody hybridization to be able to detect in the same filter different proteins. **(C)** Quantification of the western blot data by densitometric analyses. Values are normalized by actin and are the mean+SD of three replicates.

A2780 cells were selected to generate KO clones for *CDK12* with the CRISPR/Cas9 gene editing tool. As already reported (28), this was not very successful as we obtained only one clone from A2780 (A2780 KO). Sequencing of genomic DNA showed the A2780 KO clone was homozygous in exon 1 of *CDK12*, carrying one allele harboring a deletion of 30 base pairs and one containing an insertion of 270 base pairs (**Figure 1A**). These data were confirmed by the cDNA sequencing of the *CDK12* KO cells (data not shown).

We tested whether our guide RNAs could be directed against other genes besides *CDK12* (off-target genes). We used the online CRISPR Design tool by Zhang laboratory (http://crispr.mit.edu/) and found no off-target sequence in other genes.

Alterations in *CDK12* sequence are predicted to code for probably non-functional proteins. In fact, deletion in one allele (30 bp deletion) would produce a protein lacking 10 AA; the other allele has a 270 bp insertion that causes a number of stop codons, implying that no full protein should be formed. No expression of CDK12 protein in A2780 KO cells was observed, using both a C-terminal and a N-terminal epitope directed CDK12 antibody (**Figure 1B**). We carefully checked for extra bands (possibly CDK12) with different sizes, but we never found any. Considering that one allele is predicted to produce a protein lacking 10 AA, the fact that we could not find any CDK12 protein by western blot suggests that the encoded protein is very unstable.

We checked the levels of a number of proteins that have been associated and regulated by CDK12 (7, 29). We found that the basal level of RNA polymerase II phosphorylation was similar in WT and KO *CDK12*, while slightly lower levels of the CDK12 cyclin partner cyclin K (**Figures 1B, C**). The levels of CDK9, the other transcriptional CDK involved in Ser2 RNA polymerase II phosphorylation, were not changed, nor were those of CDK13 (**Figure 1B**). p53 protein levels were similar in A2780 and the corresponding CDK12 KO clone (**Figure 1B**).

### Biological Characterization of the *CDK12* KO Ovarian Cancer Cells

**Figure 2:**
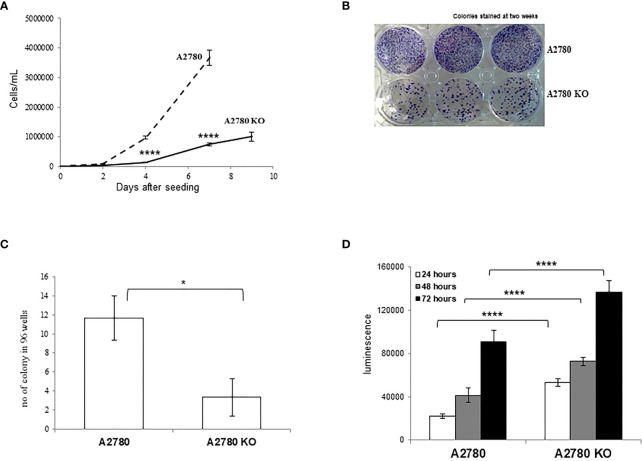
Biological characterization of A2780 and A2780 *CDK12* KO cells. **(A)** Growth curves for cells/mL (— A2780 cells; ---- A2780 *CDK12* KO cells) at different times from seeding. Data are the mean ± SD of three replicates and each experiment was performed three times. t-test was used for statistical analysis (****p < 0.0001). **(B)** Colony assays performed in six-wells plates of *CDK12* WT (upper wells) and KO (lower wells) cell lines. Cells were stained two weeks after seeding. The results shown are representative of three independent experiments. **(C)** Number of colonies in 96-wells plates. Data are the mean ± SD of three replicates done three times (*= p<0.0082). **(D)** Apoptotic signals 24 (white), 48 (grey) and 72 (black) hours from the seeding. Data are the mean ± SD of six replicates done three times. For statistical analysis, two-way ANOVA, followed by Tukey’s test was used to compare *CDK12* KO with the WT cell line at each time (****p < 0.0001).

*CDK12* KO cells were morphologically indistinguishable from their parental counterparts (data not shown). We characterized the clone for its proliferation rate and clonogenicity. KO clones had slower growth than their parental cells (**Figure 2A**). Colony and limiting dilution assays suggested that A2780 *CDK12* KO cells had lower clonogenic ability than parental cells. *CDK12* KO cells did have less colony formation ability (**Figure 2B**) and less single cell growth in the limiting dilution assay (**Figure 2C**). We wondered whether the absence of CDK12 led to an increase in cell death that could partly explain the differences in cell growth. As shown in **Figure 2D**, the percentage of cells undergoing apoptosis was higher in *CDK12* KO cells than in their parental cell line at different times. There was no activation of autophagy, as demonstrated by the absence of a cleaved form of LC3 protein and the increase in p62 (**Supplementary Figure 1A**), or senescence, as indicated by the absence of β-galactosidase staining (**Supplementary Figure 1B**) in *CDK12* KO cells.

**Figure 3:**
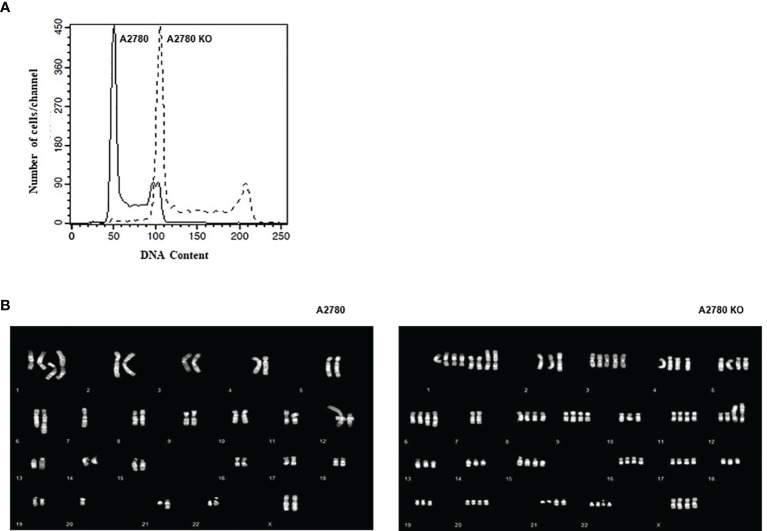
**(A)** Flow cytometric analysis of DNA content of the cell lines. **–**: *CDK12* WT cell lines, - - : *CDK12* KO cell lines. **(B)** Composite karyotypes of A2780 cell line (left panel) and of A2780 *CDK12* KO (right panel).

A2780 and *CDK12* KO cells were analyzed for their DNA content and distribution in the different phases of the cell cycle. A2780 and A2780 *CDK12* KO cells were regularly distributed in the different phases of the cell cycle (**Figure 3A**). However, A2780 *CDK12* KO cells had a twice DNA content as compared to parental cells.

Considering the recent report of an association of bi-allelic inactivation of *CDK12* with tandem duplication (17), we analyzed the karyotype of the cells to investigate the number and structure of the chromosomes. The composite reconstructions of WT and KO karyotypes (**Figure 3B**) showed that structural rearrangements of A2780 were maintained in A2780 *CDK12* KO cells, but duplicated. The A2780 cell line (upper panel) showed a modal number of 46 chromosomes, ranging from 44 to 47 corresponding to a diploid DNA content. Its composite karyotype is 44~47,XX,-1x2,+der (1) dup (1q),+del(1)(p36.3p32),+der(1)t(1);?(q11);?x2,-4,der(6)t(1;6)(q21.2;q22),7, der(7)add(7)(q32), der (12)t(?;12),del(14)(q23),der(21)add(21)(q22). The A2780 CDK12 KO cells line had a modal number of 90 chromosomes, ranging from 88 to 95 (lower panel), in line with the doubled DNA content shown by FACS analysis. Its composite karyotype is 88~95,XXXX,-1x4, +der(1)dup(1q)x3, +del(1)(p36.3p32)x2,+der(1)t(1);?(q11);?x4,+3,-4,+4,+5,der(6)t(1;6)(q21.2;q22)x2,-7x2,der(7) add (7) (q32) x 2, -10, der (12) t (?;12) x 2, -13x2, der (13;14)(q10;q10),-14,del(14)(q23)x2,-17,-18,-19,-21, der (21)add(21)(q22)x2.

### *CDK12* KO Effect on Tumor Growth *In Vivo*

We transplanted parental and *CDK12* KO cells in nude mice and examined their tumor take and tumor growth. The A2780 KO tumor lag was much longer than A2780, all the mice having a palpable tumor by day 135, while all the animals transplanted with A2780 cells were dead with tumors by day 25 (**Table 1**). Tumor growth rates of A2780 *CDK12* KO cells were slightly lower than A2780 cells (**Supplementary Figure 2**).

**Table 1:** No of mice tumor/no of transplanted mice with A2780 and *CDK12 KO* A2780 cells.

Days after tumor implant	A2780 xenograph	*CDK12* KOA2780 xenograph
10	0/4	0/4
25	04/04	0/4
15		01/04
45		02/04
75		03/04
135		04/04

### *CDK12* KO Effect on Invasiveness

CDK12 was reported to modulate the alternative splicing of specific genes, increasing the tumorigenicity and aggressiveness of breast cancer cells (30). We therefore investigated whether *CDK12* KO cells showed a difference in migrating from their parental cells. In chemotaxis experiments (**Supplementary Figure 3**) there was no difference in migration between parental and *CDK12* KO clone. In wound healing assay, we found no differences in the ability to repair the wound in *CDK12* KO cells compared to the parental cells (data not shown).

### CDK12 KO Effect on DNA Damage and Repair

**Figure 4:**
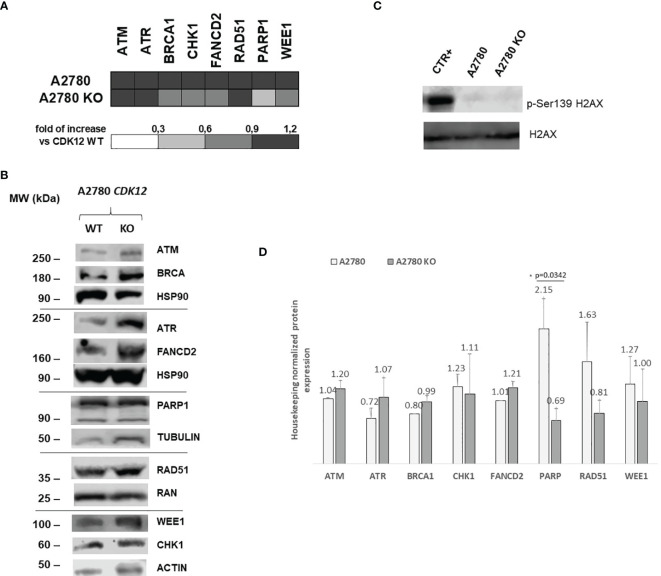
DNA repair gene expression in A2780 and A2780 CDK12 KO cells. **(A)** mRNA levels of *ATM, ATR, BRCA1, CHK1, FANCD2, RAD51, PARP1, WEE1* in A2780 and A2780 *CDK12* KO cell lines obtained by RT-PCR. Values are the mean+SD of three independent experiments, done in triplicates and are expressed as the fold of change over the *CDK12* WT cell line. **(B)** Western blot analysis showing ATM, HSP90, ATR, FANCD2, BRCA1, PARP1, RAD51, β tubulin, CHK1, WEE1, ACTIN, p-Ser139 H2AX, H2AX protein levels in A2780 and A2780 *CDK12* KO cells lines. All the protein blots for western analysis have been cropped before antibody hybridization to be able to detect in the same filter different proteins. **(C)** Western blot analysis showing p-Ser139 H2AX, H2AX protein levels in A2780 and A2780 *CDK12* KO cells. All the protein blots for western analysis have been cropped before antibody hybridization to be able to detect in the same filter different proteins. **(D)** Quantification of the western blot data by densitometric analyses. Values are normalized by actin and are the mean+SD of three replicates.*p = 0.0342.

*CDK12* down-regulation has been reported to be associated with lower mRNA and protein levels of DNA repair genes (4). We investigated the levels of a number of DNA damage genes in *CDK12* KO cells compared to those in parental cells. **Figure 4A** shows the relative mRNA levels of some of the genes acting in DNA damage and repair, expressed as the fold of increase over the parental cells. There was a partial downregulation of *BRCA1, CHK1*, and *WEE1* in A2780 KO cells, while *PARP1* was downregulated. Protein levels of the same genes (**Figures 4B, D**) assessed by western blot, confirmed the downregulation of PARP1, while no significant change of BRCA1, CHK1, and WEE1 protein levels. We then checked for the level of endogenous DNA damage evaluating the activation of H2AX, a *bone fide* marker of this. No increase in p-Ser139 H2AX was detected in *CDK12* KO cells compared to the WT cells, suggesting no greater DNA damage in *CDK12* KO cells (**Figure 4C**).

Considering the reported role of the cyclin K/CDK12 complex in eukaryotic gene expression and to gain a broader picture of changes in gene expression, we compared the baseline gene expression profiles in KO versus WT cells, as described in Materials and Methods. In *CDK12* KO cells, 6.8% of genes were up-regulated in A2780 KO and 7.6% were down-regulated (**Supplementary Table 3**). We did not find the reported (4) enrichment in DNA repair genes in A2780 CDK12 KO cells. However, among the up-regulated genes there was significant enrichment of genes involved in apoptosis, in agreement with the experimental data (**Figure 2D**) (**Supplementary Table 4**).

### CDK12 KO Effect on Sensitivity to Anticancer Drugs

**Figure 5:**
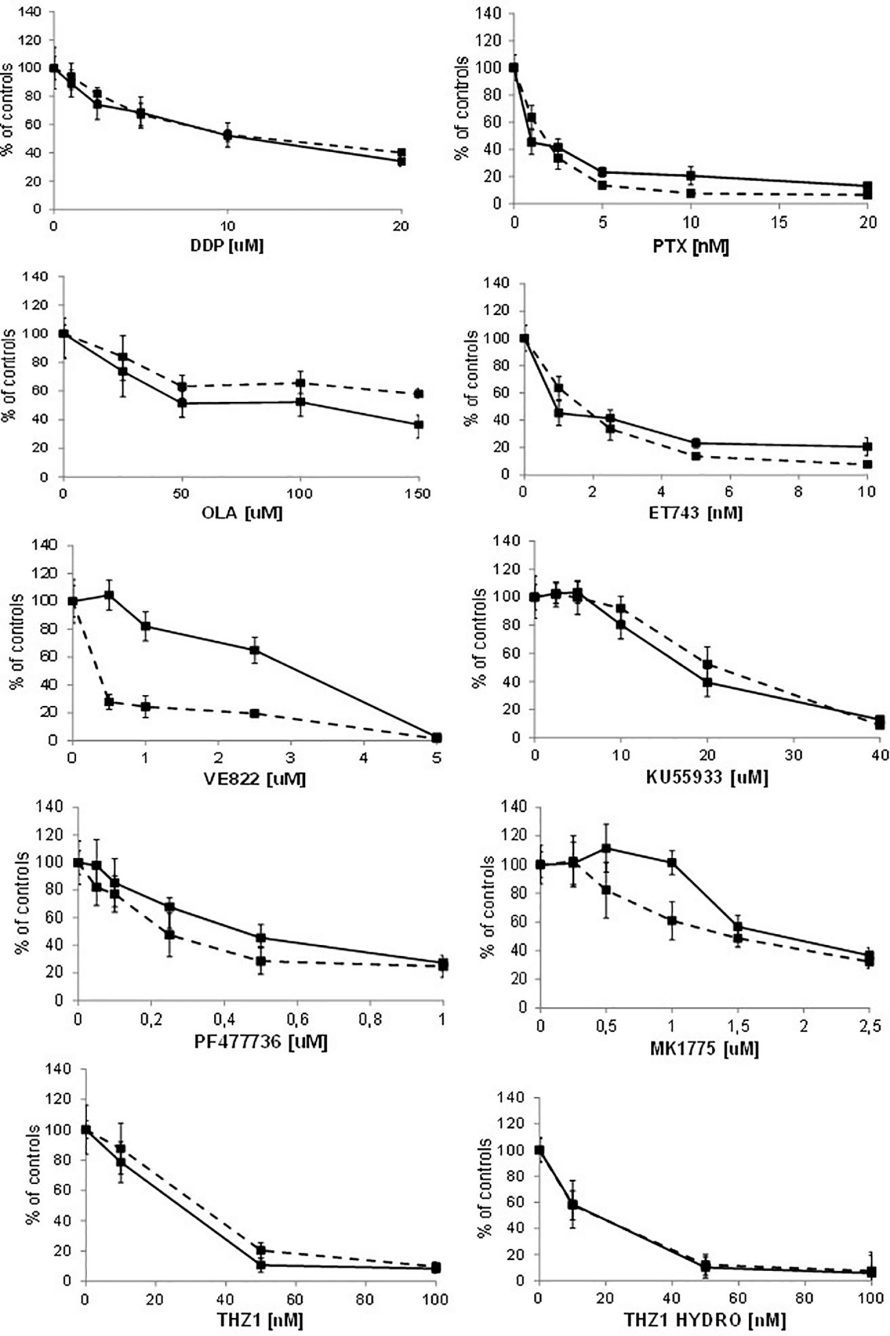
Drug dose-response curves. Dose-response curves of A2780 - - -■- - - and A2780 KO ▬▬■▬▬ to cisplatin, paclitaxel, olaparib, ET-743, VE822, KU55933, PF477736, MK1775, THZ1, THZ1 hydrocloride. Data are expressed as survival percentages over controls (+ standard error) of least three experiments done in quintuplicate.

Recent reports suggest that cells with *CDK12* mutations and/or transient downregulation with small interference RNA are more sensitive to alkylating agents, PARP inhibitors and irinotecan (11, 12, 31). We pharmacologically characterized A2780 *CDK12* KO cells compared to parental cells using agents with different mechanisms of action; DNA interfering agents: cisplatin (DDP), ET-743, olaparib (OLA); antitubulin agents: paclitaxel (PTX), and agents targeting the DNA damage response pathway: ATR inhibitor (VE822), ATM inhibitor (KU55933), CHK1 inhibitor (PF477736), WEE1 inhibitor (MK1775) and CDK7/CDK12 inhibitors (THZ1 and THZ1 HYDRO). Contrary to some reports, KO clones were not more sensitive to DDP and OLA; the patterns of sensitivity were also similar with ET-743, PTX, KU55933, PF477736 and MK1775. *CDK12* KO cells showed the same sensitivity to THZ1 and THZ1 HYDRO as WT cells (**Figure 5**). Interestingly, *CDK12* KO cells were five fold more resistant to the ATR inhibitor VE822 with an IC50 value of 1.99 + 0.81μM versus 0.39 + 0.07 μM (**Figure 5**; **Supplementary Table 5**).

To see whether the cells’ sensitivities to DDP and VE822 were due to some changes in apoptosis, we ran an apoptosis test 24, 48 and 72 hours after treatment with different drug concentrations. **Supplementary Figure 4** shows the apoptotic levels of treated and untreated A2780 and A2780 *CDK12* KO cells. There were no differences in DDP-induced apoptosis between the WT and *CDK12* KO cells, while VE822 caused weaker induction of apoptosis in KO than in WT cells. These data suggest that the lower sensitivity of A2780 *CDK12* KO to VE822 than A2780 may be partly due to a lower induction of apoptosis.

## Discussion

Cyclin-dependent kinase 12 (CDK12) has been reported to favor the maintenance of genomic stability as it promotes the transcription of a subset of genes involved in the DNA damage response (4, 5). *CDK12* inactivation causes downregulation of genes involved in DNA repair (10, 11) and increases sensitivity to platinum agents and PARP inhibitors (12, 13). Mutations in *CDK12* have been reported in different tumor types (7, 8) and it is one of the most mutated genes in ovarian carcinoma (9). Recently, we reported that CDK12 mRNA levels were predictive of platinum response in a xenobank of patient-derived ovarian carcinomas (32). To understand CDK12’s role in tumor growth and response to therapy better, we here generated ovarian cancer cells knocked out for *CDK12*.

The very low rate of success in obtaining *CDK12* KO clones using the effective CRISPR/Cas9 tool suggests CDK12 is important for cell viability (5, 28). To the best of our knowledge, no stable *CDK12* KO cells have been described; only an analog sensitive *CDK12* cellular system has been published (28), a single HeLa cell clone in which the only functional copy of *CDK12* was selectively inhibited by a cell-permeable adenine analog. In this system, CDK12 inhibition resulted in an arrest in cell proliferation and perturbation of the phosphorylation pattern of RNA polymerase II CTD. The cell growth of *CDK12* KO cells confirmed this, as the lack of CDK12 delayed cell growth and reduced the clonogenic ability of the *CDK12* KO cells. The data were also partially confirmed *in vivo*, where A2780 KO tumors were as tumorigenic as A2780 cells. However, their time lags were almost 9-13 times those of A2780 cells, and their growth rates were slightly lower than the parental cells. These data seem to contrast with the reported evidence of CDK12 as tumor suppressor. However, as recently suggested (33), if important events in oncogenesis depend on the loss of CDK12 catalytic activity leading, among others to deregulation of DDR, we did not observed in our experimental setting an impairment of response to damage in *CDK12* KO cells. As regards CDK12’s implication in tumor invasiveness and aggressiveness (30), we did not find any differences in migration capacity between WT and KO clones.

The absence of the CDK12 protein is not associated with any clear decrease in RNA pol II p-Ser2 phosphorylation, probably because CDK9 and CDK13 levels-which induce the same phosphorylation- were unchanged (34). Of note, CDK13 is evolutionarily and structurally related to CDK12 kinase, sharing the same cognate cyclin K and biochemical assays reported similar kinase activity and phosphorylation of Ser2 of the CTD heptad sequence (33). Even some studies support gene selective modulation and non-overlapping function for CDK12 and CDK13, evidence have recently been put forward on a significant redundancy between these two kinases and on the fact that both are key regulators of global POLII processivity (35). There was a slight decrease in cyclin K levels, never reaching statistical significance in *CDK12* KO cells. The pre-replicative assembly complex (licensing) is a requisite for DNA replication and sustains cell proliferation (36). Cyclin K is essential to promote efficient licensing in G1 phase and *CDK12* knockdown prevents the pre-replicative complex assembly in G1 (37). This might explain a DNA replication defect in *CDK12* KO cells, leading to slower growth.

Contributing to the slower growth in *CDK12* KO cells is the significant downregulation of genes involved in the G2/M checkpoint observed with gene expression analysis, and the higher basal level of apoptosis in *CDK12* KO than WT cells. In A2780 *CDK12* KO cells we did not find any increase in DNA damage, as demonstrated by the comparable levels of γH2AX phosphorylation in contrast to the reports in reduced licensing conditions (38) and in *CDK12*-null embryos (4, 5), suggesting a more complex picture.

*CDK12* KO cells have double the DNA content of WT cells, even with regular distribution in the cell cycle, with doubling of the chromosomes and retention of the structural rearrangements seen in the parental WT cells. It has been recently demonstrated that ovarian cancers with inactivating mutations of *CDK12* present genomic instability characterized by hundreds of tandem duplications of up to ten megabases (MB) in size (*CDK12* TD-plus phenotype) (17). In prostate cancer, the bi-allelic inactivation of *CDK12* is associated with a large number of focal copy-number gains dispersed across the genome (39). This phenotype was mutually exclusive with *BRCA1/2* mutations (germline and/or somatic) and with *BRCA1* promoter methylation and was not associated with genomic homologous recombination deficiency, partially contrasting with the data suggesting a decrease in the expression of genes involved in homologous recombination repair (4, 40). It was also reported that large genes (including the DNA repair genes) were not markedly abundant among the downregulated ones.

Given the role of CDK12 in regulating the expression of genes involved in DNA repair, we investigated the levels of some of these genes in our *CDK12* KO cells. There was a slight tendency to mRNA downregulation of some genes involved in DNA repair (i.e. BRCA1, CHK1, WEE1 and PARP1) not always supported by a downregulation at protein level. This apparently inconsistency could be due to other level of regulation at protein level, such as post-translation modifications interfering with their stability (41). As we focused on a limited number of DNA repair genes (those whose mRNAs were downregulated in CDK12 transient KO conditions), we might not have captured the whole spectrum of altered transcription. However, a wide gene expression analysis indicated a certain proportion of deregulated genes (both up- and down-regulated), but we did not find any clear enrichment in DNA repair genes among the downregulated transcripts as reported elsewhere (4, 40). However, the fact that *CDK12* KO cells have double DNA content suggests that the two-fold increase in DNA repair genes copy number could potentially lead to an increase in their transcripts, even in the absence of CDK12. A detailed gene copy number analysis coupled with transcriptome of the clones would help clarify this issue.

Although the biological and molecular characterization of our *CDK12* KO clones is compatible with the known function of CDK12 in cells, we could not find any differences in the sensitivity of CDK12 KO cells to cisplatin, PARP inhibitor olaparib, ET-743, KU55933 (an ATM inhibitor) and paclitaxel compared to WT cells. The ATR inhibitor VE822 was less active in *CDK12* KO clones than in WT cells.

These results partially contrast with reports of increased sensitivity to platinum-based drugs and olaparib in cells transiently transfected with shRNA and siRNA against CDK12 (11, 31, 42). Possible explanations can be offered: i) the experimental settings are quite different, as our *CDK12* KO cells had a stable, not transient, loss of CDK12, and may have developed some mechanisms of adaptation to survive cells; ii) given the functional redundancy between CDK12 and CDK13 (35), it could be that CDK13 can compensate for CDK12 loss; iii) our *CDK12* KO cells have double the DNA content and no significant decrease in DDR proteins. The lower sensitivity to ATR inhibitor in CDK12 KO cells - partly explained by the less induction of apoptosis - would be due to the fact that these cells are less dependent on the ATR-CHK1 axis. Finally, the similar sensitivity to the reported CDK12 inhibitors in A2780 CDK12 KO cells would suggest that these inhibitors are not specific for CDK12.

In conclusion, here we report for the first time a *CDK12* null cell line. We realize that all the data here rely on only one clone, but nevertheless all are compatible with the known function of CDK12 and support its role for survival in tumor cells too. *CDK12* KO cells showed a duplication of DNA, with doubling of the chromosomal number, corroborating the role of CDK12 in genomic maintenance. *CDK12* KO cells had reduced cell growth, no increase in endogenous DNA damage levels and no major downregulation of DNA repair genes, reported to be downregulated in transient *CDK12* KO cells. The lack of DDR gene downregulation might be partially explained by the doubling of DNA content and could justify the lack of increase sensitivity to platinum and PARPi. Detailed genome molecular study coupled with a global gene expression profile will help us understand better what kind of selection this KO clone has undergone and explain the differences from the transient *CDK12* downregulation experimental setting.

## Data Availability Statement

All data will be available at the National Center for Biotechnology Information (NCBI) Gene Expression Omnibus (GEO) (http://www.ncbi.nlm.nih.gov/geo) database; GSE183041.

## Ethics Statement

The animal study was reviewed and approved by the Italian Ministry of Health Institutional Review Board. The IRB also approved all the *in vivo* experiments performed with PDXs (Authorization n.705/2016-PR).

## Author Contributions

RC performed research, collected data, analysed and interpreted data, performed statistical analyses and wrote the manuscript; MC and FG performed the western blots, interpret the data and performed statistical analyses; NP collected data and analysed and interpreted data; DC performed research, analysed and interpreted data; AR and LC performed research, analysed and interpreted data, performed statistical analysis; FB and MF analysed and interpreted data, performed statistical analysis and wrote the manuscript; GD designed experiments, analysed and interpreted data; wrote and reviewed the manuscript. All authors reviewed the manuscript. All authors contributed to the article and approved the submitted version.

## Funding

The research was supported by a grant from the The Italian Association for Cancer Research (GD IG 19797).

## Conflict of Interest

The authors declare that the research was conducted in the absence of any commercial or financial relationships that could be construed as a potential conflict of interest.

## Publisher’s Note

All claims expressed in this article are solely those of the authors and do not necessarily represent those of their affiliated organizations, or those of the publisher, the editors and the reviewers. Any product that may be evaluated in this article, or claim that may be made by its manufacturer, is not guaranteed or endorsed by the publisher.
