# Supplementary material for: Stable CDK12 Knock-Out Ovarian Cancer Cells Do Not Show Increased Sensitivity to Cisplatin and PARP Inhibitor Treatment
Source: Front Oncol. 2022 Jul 13;12:903536. doi: 10.3389/fonc.2022.903536 (PMC9328802; doi:10.3389/fonc.2022.903536)
Supplement: Supplementary file 1 [file DataSheet_1.pdf]

Stable *CDK12* knock-out ovarian cancer cells do not show an increased sensitivity to cisplatin and PARP inhibitor treatment

Rosaria Chilà, Michela Chiappa, Federica Guffanti, Nicolò Panini, Donatella Conconi, Andrea Rinaldi, Luciano Cascione, Francesco Bertoni, Maddalena Fratelli and Giovanna Damia 1*

LEGENDS TO SUPPLEMENTARY FIGURES

Supplementary Figure 1. Western blot analysis. Panel A. Western blot analysis showing LC3 and RAN protein levels in A2780 and A2780 KO. All the protein blots for western analysis have been cropped before antibody hybridization to be able to detect in the same filter different proteins. **Panel B.** Bright-field microscopy images of A2780 positive controls, A2780, and A2780 KO (100x magnification) stained for β-galactosidase. Cells were seeded in 6-wells plates, 3 replicates for each cell line, and 48 hours later staining was performed and bright field microscopy images were captured. Arrows indicate the blue signal of the β-galactosidase positive cells (A2780 cells treated with etoposide (Sigma) 10 uM for 24 hours, then recovered for 72 hours).

Supplementary Figure 2. In vivo tumor growth. Tumor growth after A2780 (blu curves) and A2780 K0 cells (red curves) after subcutaneous transplantation in nude mice. Each curve represent a single mouse.

Supplementary Figure 3. Chemotaxis and chemoinvasion experiments. Number of cells counted in chemotaxis (**A**) and chemoinvasion (**B**) tests in the presence or absence of chem-oattractive agent. Data are represented as the mean ± SD of six replicates and each experiment was performed twice. Data are expressed as the fold increase over the control. For statistical analysis, t-tests were performed to compare chemo-positive with chemo-negative values for each cell line and chemo-negative values of CDK12 KO cell lines with the parental ones (* = p value 0.02).

Supplementary Figure 4. Apoptosis induction after DDP treatment. Apoptotic signals in A2780 and A2780 KO cell lines at 24, 48 and 72 hours from the treatments with DDP 10 uM and VE822 0.25 and 3 uM . Data are represented as fold of increase of the signal in treated cells versus the one in untreated cells for both the cell lines. Turkey's multiple comparison: *p=0.0132; **p=0.0017; ***p=0.0007; ****p=0.0002; *****p<0.0001.

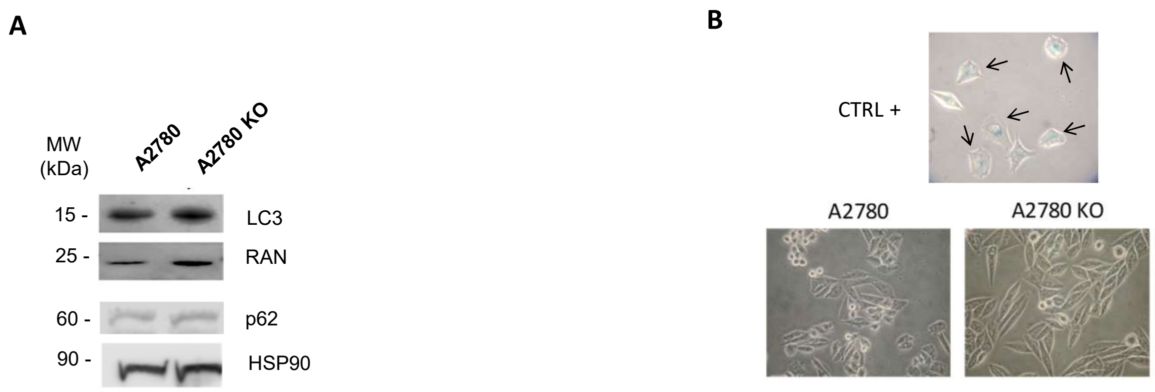

Supplementary Figure 1

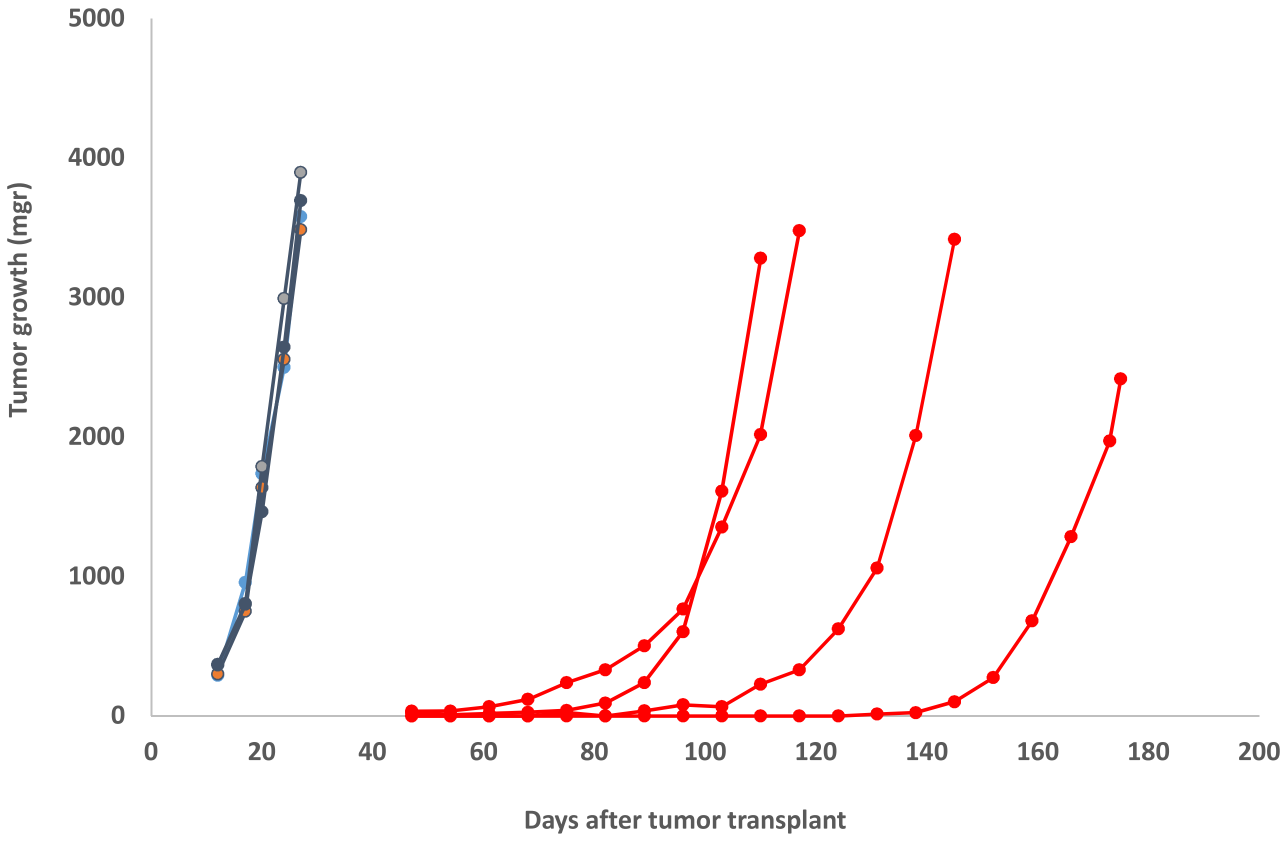

Supplementary Figure 2

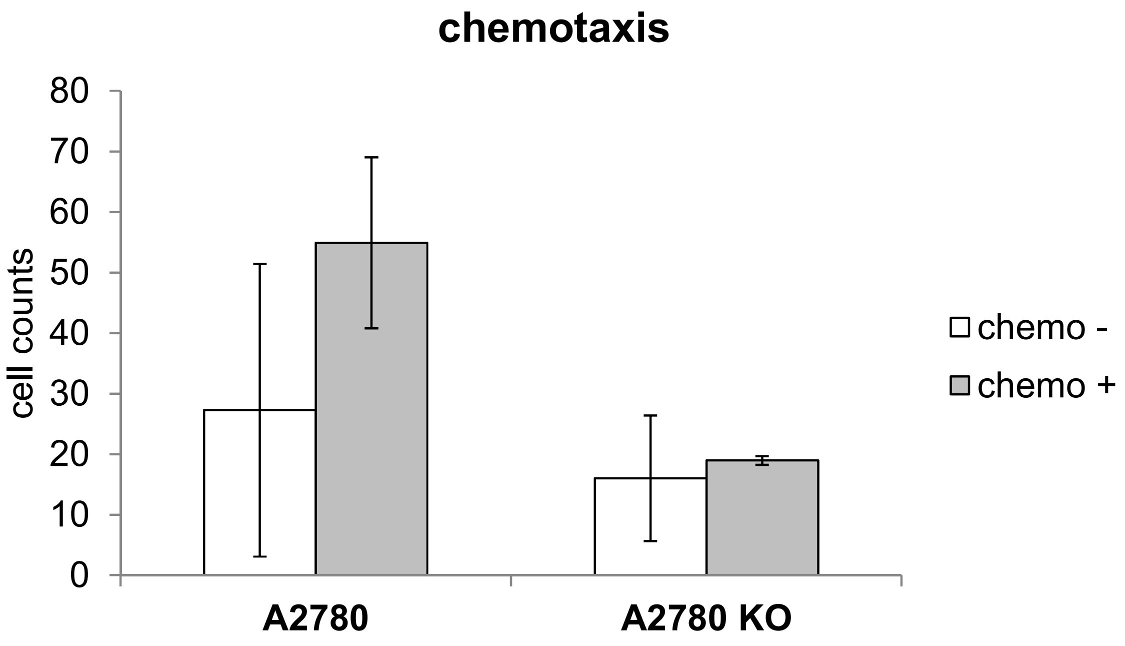

Supplementary Figure 3

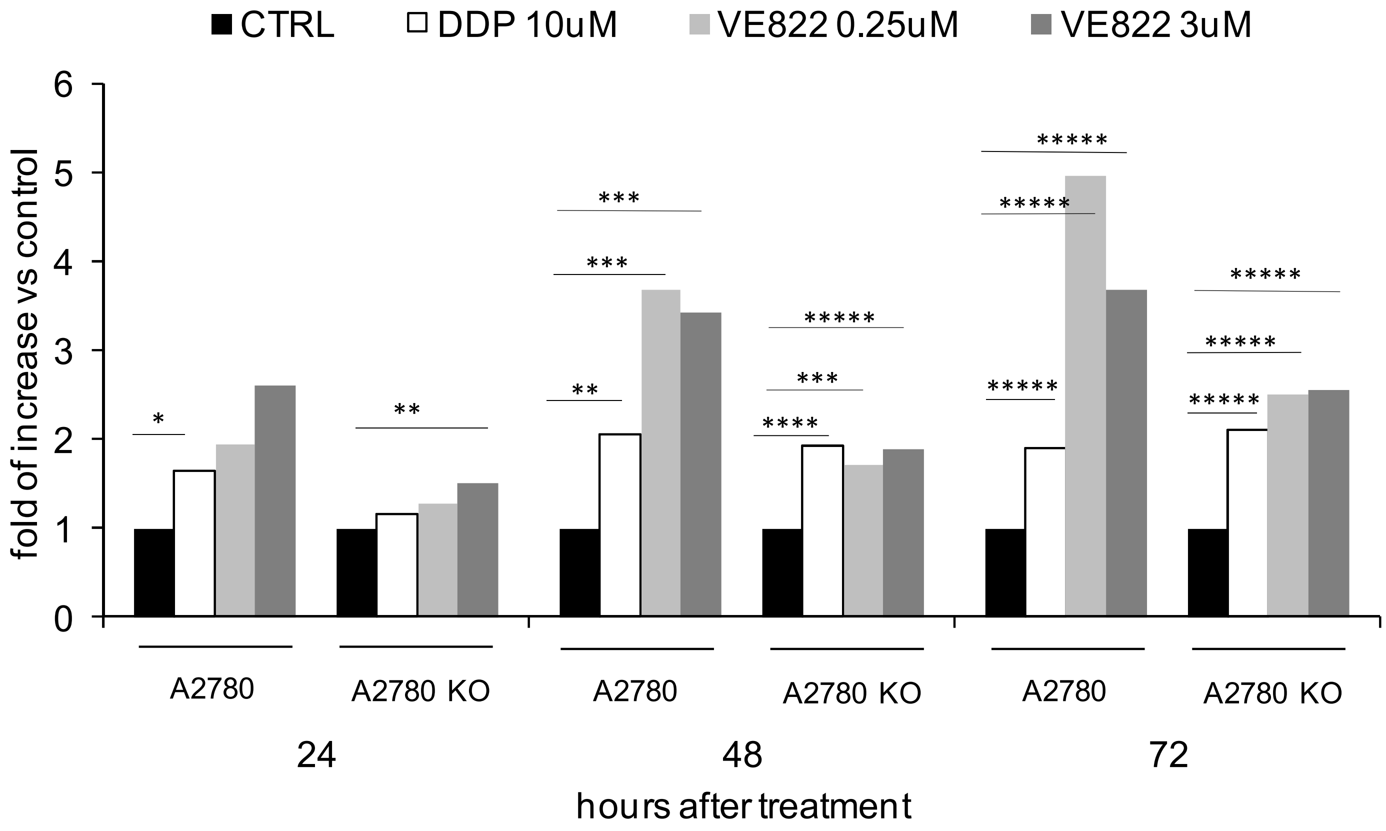

Supplementary Figure 4

Supplementary Table 1. List of primars used

Gene	FORWARD primer	REVERSE primer
**ATM**	CTGGATCGCTGTCTTCTGGG	AAGACGTGAACACCGGACA
**ATR**	TTGGATGTGCTTGGAATTGA	CCTGTTGAGTTTGGCATTGA
**BRCA1**	GCCAGAAAACACCACATCAC	CAGTGTCCGTTCACACACAA
**CDK12 (DNA)**	TCAGGTCAGGGGAAAGGGAA	GGTGGTGACGATGTTTGTCG
**CDK12 (cDNA)**	GTTCCCGGGACTTACTAAAAGCT	CTACTGTAGGGGCTCGGTGA
**CDK12 (cDNA)**	TCACCGAGCCCCTACAGTAG	GAGTCTCTTGTTCCCTGTGCT
**CDK12 (cDNA)**	GCACAGGGAACAAGAGACTCT	GGTTGCTGAGGTGGTGTGAT
**CDK12 (cDNA)**	ATCACACCACCTCAGCAACC	ACGTTCCTCTCCTAGCAGTAGT
**CDK12 (cDNA)**	AGATGTTTGGAGCTGTGGATGT	TGTGCCATTTGAGGGATGCT
**CDK12 (cDNA)**	CCAACCCAGAGATGCAGCA	TCAGGCCCATCAGTGTTTCC
**CDK12 (cDNA)**	ACACTGATGGGCCTGAAACA	AGTGAGCAAGGAATACAACGGA
**CHK1**	GACTGGGACTTGGTGCAAAC	CACTGCGACTGCTTCTTCAG
**FANCD2**	CCCATCTGCTATGATGATGAA	CGTATTTGCTGAGGGGATATG
**RAD51**	CAGATGCAGCTTGAAGCAAA	TTCTTCACATCGTTGGCATT
**PARP1**	AAGAAATGCAGCGAGAGCAT	CCAGTGTGGGACTTTTCCAT
**WEE1**	ACCTCGGATACCACAAGTGC	TACCAGTGCCATTGCTGAAG
**CYCLOPHILLIN**	GACCCAACACAAATGGTTCC	TTTCACTTTGCCAAACACCA

Supplementary Table 2. List of antibodies used

Antibody	Catalog #	Provider
HSP90	sc-69703	Santa Cruz Biotechnology
p53	sc-126	Santa Cruz Biotechnology
CDK9	sc-8338	Santa Cruz Biotechnology
RAN	sc-1156	Santa Cruz Biotechnology
ATR	sc-1887	Santa Cruz Biotechnology
FANCD2	sc-20022	Santa Cruz Biotechnology
BRCA1	sc-642	Santa Cruz Biotechnology
CHK1	sc-8408	Santa Cruz Biotechnology
WEE1	sc-5285	Santa Cruz Biotechnology
actin	sc-1615	Santa Cruz Biotechnology
β tubulin	sc-9104	Santa Cruz Biotechnology
H2AX	sc-10807	Santa Cruz Biotechnology
cyclin K	sc-81842	Santa Cruz Biotechnology
RNA pol II pSer2	#13499	Cell Signaling Technology
RNA pol II	#14958	Cell Signaling Technology
PARP1	#9542S	Cell Signaling Technology
ATM	ab-78	Abcam
RAD51	ab-213	Abcam
CDK12	NB100-87011	Novus Biologicals
CDK13	NB100-68268	Novus Biologicals
LC3	MBL PM036	MBL International Corporation
H2AX p-Ser139	05-636	Millipore

Supplementary Table 3. Fold gene expression in A2780 KO versus A2780 cells

	A2780	A2780	A2780		A2780	A2780	A2780
gene_name	logFC	AveExpr	adj.P.Val	gene_name	logFC	AveExpr	adj.P.Val
LINGO2	7,36725	-1,31023	2,83E-11	RP11-206M11	-10,762	-0,10861	4,24E-11
TSPAN32	8,057195	-1,29625	8,78E-12	MGAT3	-6,88524	0,419664	2,5E-13
RP11-438B23.	6,001025	-2,49575	9,22E-09	MMP10	-7,96528	1,070888	1,81E-16
VSIG8	5,843853	-1,93295	2,26E-08	SLC8A1	-9,70874	0,517452	4,39E-13
NR3C1	7,768343	3,629685	1,1E-12	SPRY1	-10,3069	0,873059	2,72E-15
FN1	0,73781	6,138446	0,000144	MMP1	-7,20892	3,429273	3,14E-20
CADM2	6,253886	0,116284	2,57E-11	CALB1	-1,74509	-1,87362	0,040193
C11orf21	5,581768	-0,76335	2,95E-11	PEG10	-10,8302	2,572322	3,88E-13
USP32P1	6,47586	0,153506	2,88E-09	ID4	-8,94097	1,284367	3,61E-12
ACKR3	1,511795	-2,14993	0,009643	FIGN	-11,6037	3,398889	4,35E-13
BEAN1	5,650005	1,619407	4,16E-12	IGFBP4	-7,67524	-0,91655	3,49E-10
SLIT2	7,027041	5,21663	1,28E-16	TM4SF1	-11,2583	4,884341	4,35E-13
TLR1	4,789684	0,868909	1,16E-11	EYA4	-11,0659	2,960964	1,43E-12
CFAP45	5,85759	1,614299	9,02E-15	ANKRD1	-6,43773	-0,20673	1,33E-08
SMARCA2	6,54459	4,149509	1,15E-15	TM4SF18	-8,69821	-0,97187	1,26E-10
LINC01376	5,294024	-0,645	5,94E-08	AP1M2	-5,7703	-0,28069	7,04E-07
CCDC144B	5,550574	-0,79178	1,9E-08	LUM	-4,85611	-1,2077	5,3E-09
CPEB2	5,708792	3,253608	7,9E-11	GPNMB	-9,00756	1,223951	1,59E-13
CCDC144A	5,302191	0,88222	6,91E-07	SAMD5	-7,62704	1,177299	2,08E-12
AC005083.1	5,032603	-1,40034	3,97E-09	CDH10	-9,10892	-0,51669	1,18E-12
PHYHIP	4,94877	-0,42053	3,12E-10	NEDD9	-5,6882	0,321713	1,49E-09
CRISPLD2	2,950187	0,562757	1,17E-06	MARCKS	-5,98813	0,895938	1,35E-08
RP11-958N24.	1,054255	-0,94407	0,036684	HMCN1	-5,87379	-0,15636	1,19E-06
RP11-219A15.	4,70777	-0,66297	1,84E-07	HOTAIR	-7,0833	-1,13521	3,28E-10
RNLS	4,781784	-0,09324	3,26E-08	TSPAN18	-5,7151	-0,6345	3,95E-09
AC005592.1	2,580749	0,889648	4,52E-11	HOXC11	-8,2123	-2,4794	7,94E-12
RP11-274H2.5	2,189995	-1,0976	0,001447	MMP3	-7,71298	-0,50134	9,07E-16
CYP2E1	0,859721	2,568011	0,000207	SORBS2	-1,93427	-1,93691	0,023063
AC005592.3	2,621166	-1,21321	2,67E-06	LINC01091	-8,06266	-2,79389	3,34E-11
RORC	5,270508	-0,56323	7,13E-10	EPHA7	-7,26461	-0,90912	4,16E-10
NTNG2	2,564531	-0,54552	0,000761	LOXL4	-7,61485	-2,53857	1,76E-10
PLXNB3	2,877311	2,391516	2,97E-12	CYP27C1	-6,34734	0,413207	9,04E-10
ZMYND12	0,828773	-0,76379	0,001478	TGFB2	-8,76473	2,096628	4,03E-12
APOL1	4,574314	3,103576	1,68E-07	DPY19L2	-7,04296	-2,88548	1,7E-11
C4B	1,511127	-0,51933	0,038738	LMO4	-8,55406	4,772373	1,26E-17
VSIG1	1,451076	-1,22559	0,037012	RASGRP3	-7,46663	-1,21549	6,09E-13
ARHGAP31	2,700432	3,692716	5,96E-16	PTGIS	-5,293	0,794725	3,42E-08
MX2	1,835365	-2,45821	0,021371	ROBO2	-7,04226	-2,68694	3,58E-11
SLC25A27	5,12691	2,99608	1,13E-07	RASGRF1	-5,41762	-1,9474	4,5E-08
SIM2	5,336045	3,907251	9,4E-13	TMCC3	-3,17011	-0,63653	1,44E-05
OAS1	3,358535	2,196031	2,08E-09	IGF2BP1	-6,81929	1,83891	7,6E-18
PSD2	2,151916	0,417489	4,03E-07	PDGFB	-8,21425	1,122254	3,63E-10
TRPM8	1,977677	-0,83401	0,00143	ZFHX4	-4,64522	0,285157	8,8E-06
LINC00578	1,27942	0,171214	4,32E-05	DMD	-7,80092	1,119292	4,66E-16
LINC01098	1,682596	-1,82181	0,029416	ADD2	-7,17552	-0,98076	2,32E-11

FBLN7	2,460063	3,640493	2,62E-16	SH3RF2	-3,74006	1,426243	3,82E-10
RGAG1	2,91761	-1,79047	1,31E-05	CCDC144NL-AS	-7,12136	-1,49903	5,79E-11
MST1	1,991321	2,709189	2,17E-08	COL1A2	-0,79277	7,34353	8,3E-09
RP11-91K9.1	2,356171	-2,30974	0,000425	CPM	-5,9229	-0,24972	1,59E-09
KLF12	4,502129	1,245895	4,66E-07	CTD-2006C1.2	-7,43182	1,352103	7,08E-11
PSD3	0,251692	4,491728	0,003728	UCA1	-3,47055	0,696454	0,011657
C1orf204	3,702101	1,165138	9,24E-09	PPP1R14C	-0,63521	-0,23672	0,018857
BCL6	2,80295	3,310293	4,63E-07	FLJ26245	-6,16526	1,009044	5,59E-09
TFPI	3,891548	4,945464	2,48E-13	FLRT2	-7,27181	-3,19429	1,7E-11
DLL1	2,985786	-0,47932	3,17E-06	PSAT1	-6,63242	5,615548	9,81E-11
CREB5	4,359917	3,946344	6,26E-11	TMEM236	-5,65423	0,558024	3,24E-13
ZHX2	4,734922	2,153706	2,09E-07	BMP5	-7,80061	0,611601	1,77E-11
SH3TC1	3,886146	1,381339	8,14E-11	PSG9	-3,61433	-0,47456	9,38E-06
RP11-399B17.	1,862411	2,172258	1,64E-10	PCDH7	-7,28328	1,010291	1,02E-10
TF	1,435921	-0,38154	0,000501	SERPINB9	-7,54506	5,043463	3,02E-17
MROH7	2,388163	-0,90667	1,24E-07	NTS	-6,34573	-2,5246	1,01E-08
ALOX12P2	3,482027	-0,0602	5,62E-05	TM4SF1-AS1	-6,77433	-2,5107	1,57E-10
FAM65C	1,425743	2,0718	1,04E-10	NPTX2	-6,43771	-1,97069	4,66E-10
UBA7	3,143238	3,286939	3,16E-09	GPR110	-4,13954	-1,75848	4,19E-05
WNT11	0,968903	-1,40232	0,00127	MECOM	-2,45994	0,53707	0,003208
LRIG3	4,407348	2,870999	6,61E-10	HDAC9	-1,74019	0,892852	5,53E-11
ANKS1B	1,60759	-0,46044	0,039933	QPRT	-6,86055	4,398585	4,91E-12
GRIP1	2,784597	0,963885	5,47E-05	SEMA3A	-4,18173	-0,02568	2,18E-06
ARRDC3	1,660659	6,168314	3,02E-11	DPYD	-7,39024	2,280942	3,15E-11
MIRLET7BHG	1,208423	4,385534	1,76E-07	ALDH1A3	-5,99545	-0,16383	2,43E-09
PCDHB7	1,918098	1,968215	2,09E-06	SEMA3D	-6,22312	-2,21642	1,89E-09
NLRP1	2,803071	5,04907	1,79E-13	GLS2	-6,95211	-0,33039	1,2E-11
KCNN4	2,865937	2,892798	1,56E-08	CTGF	-5,65768	3,477279	4,19E-11
MERTK	3,085566	4,05939	6,22E-18	ZNF136	-7,16886	1,157519	9,74E-12
FAM109B	1,004423	0,541255	0,011151	CYR61	-5,9431	4,868222	1,36E-11
MKX	3,4372	2,87864	4,28E-06	TRIML2	-5,67148	0,202763	4,32E-15
RGPD8	3,435427	1,151005	3,23E-10	PCDH9	-7,15479	2,072447	2,8E-09
KRT86	2,110063	-2,05906	0,003316	PSG5	-3,80082	0,201098	1,84E-06
NUPR1	3,857569	4,755815	6,85E-07	SPATA18	-6,22252	-2,25561	3,03E-10
SLC13A3	2,234986	1,645457	2,34E-06	C6orf223	-3,48801	-1,09651	1,61E-05
PCDHB8	3,36683	1,235247	5,06E-06	YBX2	-4,82675	1,654271	5,49E-09
RP11-553L6.5	3,781768	2,122372	1,68E-07	MYOF	-7,17604	4,776313	5,93E-14
SLC14A1	2,444109	-0,87379	0,001072	ELOVL2	-5,80464	1,260263	1,91E-08
BDNF-AS	2,490672	1,131046	3,47E-07	PSG4	-4,07228	0,843208	7,4E-07
IL15	3,327127	1,497766	1,74E-05	XXbac-B444P2	-4,34308	-2,57665	4,06E-07
BTBD11	1,035739	0,917177	0,000579	TRIM58	-6,86335	1,033375	5E-11
PARP10	3,174511	4,727342	1,34E-14	ZNF442	-4,86478	0,221195	0,000225
KIAA1683	1,250593	2,085071	1,13E-05	KIAA1644	-1,68861	-2,89526	0,045525
N4BP2L1	3,288394	2,495036	1,88E-09	PCDH17	-3,90417	0,766881	1,4E-10
TSTD1	3,241057	-0,21037	2,24E-05	CEP41	-6,5219	0,948968	3,35E-10
PLOD2	1,043279	6,12792	1,26E-09	UBE2E3	-6,89248	4,223168	4,32E-15
OAS2	2,638622	1,054061	1,66E-06	LTBP1	-3,4034	3,144003	1,77E-15
SCART1	1,488987	3,501519	1,01E-07	RASL11B	-4,80215	-1,07345	7,18E-08
GABRR2	2,119257	1,192026	1,6E-06	AC006273.5	-3,2544	-1,69367	1,35E-05
FGL1	0,79586	0,246494	1,59E-05	FILIP1	-4,72034	-1,56164	7,35E-08

MSH4	2,259671	-0,22014	1,2E-05	NFE2	-4,5811	-0,3892	2,61E-10
STAT5A	2,557297	-0,52905	0,002038	GJA1	-0,2838	4,933896	0,00494
BTBD16	1,724664	-0,93742	0,000787	PRUNE2	-2,00711	-2,26448	0,043688
GRIP2	0,467446	0,040258	0,00517	IL21R	-4,43591	-1,182	5,87E-10
CD72	0,977374	1,69502	0,004538	IL1R1	-0,32129	4,169747	0,000444
TNFRSF14	1,767019	1,403831	0,003908	DUSP6	-5,05343	1,718759	6,81E-08
ITGA10	2,279666	2,578709	1,44E-10	VCAN	-6,21192	6,899135	9,41E-16
TEKT3	1,290213	-1,13396	0,017785	KIAA1549L	-1,62315	0,001959	5,88E-07
QPCT	2,902878	1,070597	1,57E-05	LZTS1	-0,50284	0,659866	0,008153
CD14	0,967776	-0,6952	0,03986	INHBB	-1,3613	-1,16472	0,00353
RAB38	2,634618	0,443499	0,000628	TNNC1	-2,65538	0,909698	5,46E-11
CTD-2619J13.3	1,626072	-0,96099	0,017635	ASB9	-2,90109	-2,61336	0,000436
RP11-153M3.1	1,657102	0,652193	9,91E-06	PLEKHG1	-1,35546	0,511419	0,000349
FCGBP	0,962327	1,975542	1,26E-06	BCHE	-2,74437	-2,57479	0,000238
CCDC162P	1,601387	-0,30193	0,001152	DOCK8	-2,43308	0,406102	7,87E-07
AC093627.10	2,183527	0,156583	2,49E-11	PODXL2	-5,78508	2,578685	1,44E-10
SSH3	2,661511	5,100268	1,94E-14	NID2	-1,70607	2,435956	1,23E-09
MITF	3,499948	4,403308	3,02E-13	NXN	-5,5185	4,193544	9,19E-14
RNF112	1,194884	0,39879	1,18E-05	GCNT2	-4,35537	0,385067	1,41E-06
CYP4F35P	1,294178	-1,69394	0,046254	PDZD2	-3,50122	1,123636	3,83E-09
RP11-1020A11	1,952728	-0,02024	0,000127	HLA-DMB	-2,561	-0,46721	4,61E-05
COL9A2	2,032556	1,837107	7,48E-06	SLC6A17	-1,83412	-1,00875	0,00312
UBL4B	1,071744	-2,07353	0,002219	SERTAD4	-1,65451	-2,35018	0,016759
PDE11A	1,563182	0,981705	1,5E-07	RIMS4	-1,54019	4,326144	2,79E-10
TCP10L	1,290816	0,301466	0,002305	PLAU	-5,87073	2,545319	5,7E-09
RP11-190A12.	1,485411	-0,09431	0,003841	SLC1A3	-1,80643	-0,83753	1,25E-07
APOL3	2,794836	-0,8633	0,000542	FGF18	-1,77457	-0,87283	0,010895
EML5	3,349169	0,454498	2,23E-05	GS1-600G8.5	-0,30732	2,417495	0,003923
SYCE1L	1,54906	-0,27799	0,004772	TSPAN12	-5,19193	0,727649	4,53E-09
CES3	0,653274	3,420326	1,16E-05	SLC47A1	-0,28434	1,646087	0,002828
RP5-1024N4.4	1,676001	-0,24779	6,46E-05	MYEOV	-3,71386	0,928981	4,72E-06
AKR1C3	3,348459	2,541411	5,38E-07	FOLR1	-3,07157	-0,60403	8,05E-05
DNAH7	1,800818	0,710148	0,000633	AFF3	-0,93641	1,272753	0,000116
NFASC	0,436576	3,145788	2,48E-05	TMEM47	-4,76768	3,747211	2,31E-12
YPEL2	1,020901	3,459008	8,13E-06	AMOTL2	-3,56432	5,822738	5,95E-13
ADAMTS10	0,443282	3,660637	0,001416	TCF4	-4,02826	2,230881	1,81E-16
IGFBP6	2,557372	6,03328	5,7E-15	RGCC	-4,09906	-0,61235	1,08E-12
SEPT7-AS1	1,326429	1,024947	4,4E-07	GJC1	-4,24841	5,345106	2,9E-15
TFAP2E	0,553723	1,601161	0,019902	FAM46B	-3,05202	1,015271	6,02E-08
RORA	2,304066	0,296982	4,48E-05	FAM81A	-3,41758	-0,29936	8,76E-07
KALRN	1,042811	2,910479	0,000353	GPR63	-4,83268	0,557365	2,09E-07
BCL2L11	1,579733	4,647632	1,15E-11	ANKRD18B	-5,01825	1,32433	1,06E-07
CTC-490E21.1	0,91963	0,366732	0,02323	LPHN2	-5,26167	1,576253	3,69E-08
VILL	1,110706	-0,28765	0,000143	RASSF9	-1,65451	-2,39619	0,027395
RP11-159G9.5	2,526043	3,464147	3,46E-10	DCLK1	-3,95126	-0,45109	5,19E-07
ROCK1P1	2,14111	1,974382	7,41E-06	XK	-1,06525	0,174923	0,000217
CTC-463A16.1	1,7802	1,002848	7,01E-06	BEND4	-2,98733	1,624036	1,2E-10
PCDHGB7	0,702916	2,113765	0,001036	CLGN	-0,51458	2,231613	0,001329
C1orf162	1,530864	1,894127	3,15E-08	PDE5A	-3,71969	0,209361	3,83E-10
PCDHB15	1,384002	2,337089	0,000277	FERMT2	-5,04662	6,334495	1,85E-20

TRANK1	2,648818	1,959506	6,13E-05	NETO2	-4,29781	1,927477	6,32E-05
SORCS2	2,761239	1,786678	0,000103	PLCB1	-4,69163	-0,02875	1,35E-08
ZNF843	1,104225	0,812457	3,51E-05	WNT5A	-0,3124	3,159371	0,001116
RP11-1391J7.1	1,527376	0,302571	0,000411	ATRNL1	-1,86374	-2,13236	0,019388
CECR6	1,764383	0,051944	2,04E-06	HOXB-AS3	-3,74084	2,33134	2,59E-10
MAPK10	2,50841	1,235863	3,03E-06	S1PR3	-1,67017	0,2757	1,76E-11
SPOCK2	2,779117	2,122419	6,5E-11	MYCL	-2,066	0,663444	2,1E-08
PRICKLE4	1,889558	3,362035	3,2E-08	IL18R1	-1,03992	0,300708	2,32E-05
NDUFA4L2	2,244185	1,125036	2,84E-06	PPFIBP2	-1,37303	1,082593	3,03E-08
C6orf3	2,505792	-1,3572	0,000214	RBM24	-2,67417	-1,63092	0,000318
CASC1	1,86141	-0,19088	0,000582	SAMD4A	-4,85107	4,55891	3,91E-15
ZNF654	2,452404	6,341873	2,3E-15	ZNF844	-4,33794	0,779358	0,000426
PPP1R3G	1,74181	-0,45599	0,034924	PDLIM3	-0,97668	2,608602	4,13E-09
MCC	0,321015	2,684027	0,00957	TNFRSF19	-3,45021	-0,68877	3,63E-07
SPEF2	0,377686	3,515671	0,039837	CCDC112	-4,39324	2,436464	6,17E-10
SHF	2,484701	2,625512	1,67E-11	PELI1	-3,83542	3,545411	1,1E-08
RP11-795F19.	1,390164	1,30094	2,56E-05	HOXB7	-4,56801	2,913884	9,78E-08
DNAAF1	1,358917	0,1903	0,024078	BAI3	-1,4142	-2,87653	0,040859
PCDHGA12	1,13685	1,390283	7,81E-06	TSPAN15	-3,14244	0,8642	2,76E-05
PCDHB10	0,559574	2,424396	0,010823	GLI1	-0,9728	2,83347	0,000186
RP1-102E24.8	2,076433	-1,49026	0,018719	ZNF675	-4,56924	3,236103	7,51E-12
GSTO2	1,70761	1,104528	1,61E-06	CHST2	-3,98487	2,959897	4,4E-12
GNAS-AS1	0,572676	0,298143	0,024792	LIPG	-1,02204	0,204733	7,23E-07
IL17RE	2,286518	0,291361	8,28E-08	RP11-301G19.	-3,63793	-0,21007	8,03E-05
CYP4F11	2,82075	-0,17301	0,000112	TFCP2L1	-0,33865	3,727375	0,001114
BMP1	1,774747	6,595108	1,96E-14	USP53	-3,94872	3,583427	2,67E-08
LCN12	2,353817	0,935147	1,05E-06	COL26A1	-0,74759	1,463158	2,63E-05
MAP1LC3C	1,46638	-1,06828	0,027183	PDE3A	-3,08414	0,711704	1,75E-16
RP11-694I15.7	1,038672	0,152029	0,003275	LINC01224	-2,13025	2,868252	9,39E-07
RP11-875O11.	2,252819	-0,32914	7,01E-06	GSG1	-2,49467	-0,45472	1,6E-10
ST8SIA5	2,162797	1,30597	4,43E-07	STXBP6	-0,68433	-0,53502	0,000443
TBX19	0,575805	2,759452	0,002228	CCNE2	-2,62781	2,057064	0,000178
AC005592.2	0,823481	0,924802	5,14E-05	NKD2	-1,02279	-0,44666	8,78E-06
SYT5	0,6638	2,711361	1,46E-06	DKK1	-1,03238	4,947451	2,41E-10
PHLDB2	2,655507	1,339248	0,001457	EFHD1	-3,94496	0,058774	4,36E-08
FHIT	1,280649	-0,20521	4,87E-06	RP11-10A14.5	-2,92867	-2,31021	7,13E-08
LRRC6	1,420865	1,554926	0,000217	LMO7	-4,24984	3,605806	1,59E-12
CTD-2517M22	1,611317	2,286173	1,19E-05	MIR4728	-2,09836	-1,79346	0,007776
GPR35	0,693545	1,554344	0,003627	HOXA13	-4,04286	0,612443	5,21E-07
GRK5	0,709182	4,005798	4,66E-07	GLI2	-2,78662	2,919311	2,01E-12
NKX3-2	2,204795	0,221286	7,13E-06	OPN3	-3,81335	0,758557	8,32E-07
AC007566.10	1,68617	1,764555	6,84E-07	MARK1	-1,43984	-0,51215	2,64E-05
HTRA1	1,527908	5,922811	6,46E-14	HCP5	-3,43703	0,424103	9,61E-09
FNDC5	2,000975	2,681674	1,68E-10	SULT4A1	-0,6594	1,993597	0,000364
ZNF462	1,822641	5,269922	1,08E-13	MEGF11	-1,89986	-1,71813	3,81E-06
TYMP	0,946226	0,149917	0,045365	SHISA2	-0,7054	-0,0487	8,79E-06
GPER1	1,514209	1,895313	4,86E-11	FGF2	-3,04839	4,809092	3,64E-12
RSAD2	1,568457	1,270028	4,31E-11	EPAS1	-3,84227	5,697589	1,3E-12
HIST2H2BE	2,300757	2,903732	4,98E-09	NUAK2	-1,93429	1,907134	0,002591
PCDHGB3	1,14285	2,736695	2,76E-06	GRAMD1B	-0,66437	1,268394	2,46E-07

LTBR	2,007954	2,650217	1,23E-05	GALNT14	-0,28381	1,557516	0,000509
S100A6	1,82212	6,397879	7,57E-08	LINC00958	-3,62451	0,46352	2,61E-06
RINL	2,201552	1,795908	7,1E-10	NOVA1	-2,93479	0,122958	7,19E-10
CA3	0,958468	-0,34001	0,043789	HOXC13	-0,61625	0,602776	3,7E-06
SMIM14	1,863648	5,257284	1,2E-11	GCA	-2,49567	1,677744	4,26E-08
PCDHGA5	1,052755	2,621916	5,83E-07	SLC30A3	-1,92344	-1,20339	7,89E-07
PCDHB19P	0,862662	-0,26092	0,040459	LINC01270	-3,69201	-0,65026	1,49E-06
CREBRF	1,624506	4,243519	1,82E-06	HES2	-1,31591	2,058949	6,8E-05
SCGB2B2	1,40991	0,505312	0,000189	AJUBA	-2,51583	5,908774	1,19E-10
TMEM87B	2,28656	6,455957	1,81E-16	ANK3	-0,36973	0,293514	0,003592
ST3GAL6	2,320974	-0,17976	3,86E-06	PRR11	-1,4599	5,488716	3,16E-12
HRH1	1,764597	0,769629	0,015925	PHLDA1	-3,81438	5,305864	3,95E-09
PERP	1,716624	5,394017	1,04E-10	FOLH1	-1,89192	-1,43284	2,23E-05
SIAE	0,888708	4,93206	3,4E-09	BCAP29	-3,95977	3,84231	4,32E-12
CD101	0,870803	0,503885	0,003185	LYPD6	-0,25489	1,246977	0,016104
HYAL1	2,354109	1,679325	9,64E-12	DTNA	-2,87757	-1,04776	6,21E-07
LRP2BP	1,280614	1,189532	4,96E-05	SH2D3A	-3,40537	-0,07423	5,32E-06
DDX26B	0,965391	4,100905	3,04E-10	PRRX2	-0,57693	-0,3014	0,000316
MTMR11	0,763804	5,913074	3,78E-08	CABLES1	-3,32725	2,536138	2,27E-09
PLSCR4	1,850517	2,195132	4,61E-07	GCNT4	-1,72707	-0,21744	3,23E-06
EAF1-AS1	1,050363	-0,53352	0,018339	SNX18P7	-3,45546	-1,64696	3,62E-06
POU4F1	1,706336	1,381604	0,002682	CDC20	-1,50705	7,001937	1,26E-11
TLR6	0,407555	2,190719	0,018022	HMGA2	-2,95077	7,288513	7,6E-18
PDLIM4	0,476935	0,148782	0,000253	CYP4F26P	-3,24109	-0,79297	5,03E-05
TSNAXIP1	1,095107	2,929598	5,86E-05	ADAMTS1	-3,75759	5,57334	1,37E-13
CTD-3088G3.8	1,928156	0,801217	2,04E-05	RASGRP1	-2,12465	-0,3508	0,024454
MFSD4	1,86139	1,565814	3,24E-12	SDC2	-3,24764	4,569798	6,84E-11
MYD88	1,606322	2,473039	1,3E-06	LINC01503	-1,52614	1,887021	7,08E-09
TIMP1	1,934073	7,93092	4,6E-16	ANO9	-2,40981	-0,32944	0,000247
CCDC13	1,621038	-0,31361	0,000121	PLIN4	-2,51715	-0,15456	8,89E-06
GDF7	1,446817	2,13884	2,35E-09	DGCR5	-0,64291	0,740566	2,03E-05
PCDHGB8P	1,108047	0,954815	0,000188	FOXD1	-2,74518	0,365682	0,002409
TMC4	1,300128	2,528642	2,22E-08	KLF8	-1,86355	-0,89958	6,8E-06
NRG2	0,79909	1,443387	0,001568	TIAM1	-0,18227	2,697363	0,022838
CA11	0,485974	5,167599	2,27E-05	VASH2	-1,41731	1,718344	4,62E-12
TCP11L2	2,171749	3,288993	2,19E-06	CGNL1	-1,44722	3,064646	2,47E-05
TPBGL	2,041964	0,413473	6,38E-06	PTPLA	-2,81861	4,203544	1,69E-13
PCDHGB9P	1,05496	0,382688	0,000355	IGFBPL1	-0,73988	0,387661	0,000372
AK4	1,831535	5,266528	1,29E-11	NFE2L3	-1,35813	2,20032	4,74E-11
RP11-513I15.6	1,961418	2,064047	1,18E-08	ZNF850	-2,88935	3,455053	8,11E-10
DNAH6	1,135936	0,880102	1,62E-05	SLC16A7	-1,54119	-1,86434	0,034553
SLC23A3	1,639466	-0,20385	0,001725	CRYAB	-0,8055	1,12762	0,021724
MDK	0,320109	6,042205	2,23E-05	TOX	-2,81466	1,393673	6,95E-06
CATSPERG	1,20579	2,528427	9,67E-07	TNS3	-3,23826	3,955134	9,5E-10
NNMT	1,704957	2,424973	0,011988	TWIST2	-0,25863	1,884604	0,018614
DDIT4	1,620436	8,210496	1,05E-07	CDC6	-0,33057	5,608959	0,004931
SPEG	0,344905	4,723694	0,001613	B3GALT5-AS1	-1,5759	-0,72459	0,004191
CMTM3	1,255964	5,611707	1,46E-13	TMEM139	-1,71755	0,144795	0,022949
INO80B	1,920889	0,035924	0,000426	NAV3	-0,21716	2,872665	0,012147
PLTP	0,587472	4,60916	8,09E-09	KCNQ2	-0,55007	1,531646	5,03E-07

PSD	1,770046	2,502109	8,58E-11	C12orf75	-1,90232	6,000152	1,3E-10
RP5-1068E13.	1,653417	0,355124	0,001071	KIAA1211	-1,45198	-0,29354	3,33E-08
KAT2B	2,20936	4,336547	3,18E-13	FBXO43	-0,76671	2,951738	0,000113
PCDHGB1	1,394217	0,560613	0,028877	FNDC1	-1,75441	0,063607	1,08E-05
AVIL	2,415586	2,05754	2,27E-08	RNF165	-0,39711	2,588096	0,033434
LA16c-306E5.:	1,708105	-1,70386	0,01368	DLGAP5	-0,76598	5,750821	7,46E-08
CTC-439O9.1	1,209941	-1,82957	0,00383	DMKN	-0,24884	3,974136	0,001334
ALPK3	1,390145	0,725345	0,001337	FAM53A	-2,67884	0,658207	2,11E-06
BANF1P2	1,278753	-0,46736	2,21E-05	PRDM8	-2,49808	0,136574	0,001625
IL12A	1,113672	0,475835	0,000108	CTSV	-1,863	3,512374	4,49E-13
RP11-182N22.	1,634119	-0,71571	0,011212	DAAM2	-0,53189	1,553052	8,84E-06
TTC34	2,041354	0,754957	4,05E-06	ADAMTS7	-0,68076	1,766642	1,4E-07
SLC16A2	0,708646	3,127189	4,2E-08	NOVA2	-2,95586	1,621345	1,68E-09
CTSF	0,403659	4,79567	0,00321	CACNG4	-1,50539	-0,43168	0,000512
AC074212.5	0,935145	1,102717	0,020105	THBS1	-1,95471	6,455614	2,17E-12
CTB-119C2.1	1,822932	0,329262	2,04E-05	MYL9	-1,64834	1,502275	1,54E-14
TNIK	1,611118	5,440919	3,96E-12	NRIP3	-3,18512	3,742601	1,59E-11
MCOLN3	1,703893	5,085539	2,26E-09	ACTRT3	-2,62741	-0,00131	0,000218
PCDHGB4	0,654566	0,877594	0,019762	SOX4	-2,17044	6,187798	6,25E-15
SHC1	1,257575	9,045409	2,92E-13	MYBL2	-0,33436	6,81341	0,004205
CREM	2,536371	4,698394	5,13E-15	SNX16	-3,05634	4,107619	2,53E-15
LEPR	1,177037	4,458163	1,09E-07	PLEKHH2	-2,21778	3,146582	1,77E-06
PCDHGB2	0,869666	3,271003	1,03E-06	STOX2	-0,15592	3,529297	0,040721
RFX3-AS1	0,942171	1,102722	0,004015	NCAPH	-0,48498	5,356111	2,03E-05
SMPDL3A	1,034599	1,15727	0,000168	SDK1	-0,97278	0,045336	0,00173
KCND3	1,922047	0,531148	4,23E-08	HES1	-2,22256	4,130322	3,28E-10
NIPAL3	1,939661	5,776536	1,09E-15	ATP2B1	-2,84902	4,806674	1,58E-12
LONRF2	2,286892	5,536243	4,68E-13	PTTG1	-1,65865	5,152143	1,59E-10
RP11-631N16.	0,814839	4,052704	0,009669	CCNB2	-0,69793	5,424097	1,53E-08
NREP	0,481348	5,061147	2,77E-05	PITPNC1	-1,75928	2,476507	8,49E-12
COPZ2	1,54368	1,84634	4,61E-05	RP11-7F17.1	-1,4142	-2,37668	0,024886
RP11-389O22.	0,869436	-0,35163	0,036753	TOX2	-0,76086	2,777827	0,006855
P4HTM	1,439051	0,955541	0,03191	RET	-1,89895	1,615578	2,45E-11
WBP1	0,99141	3,700768	0,000857	CENPA	-0,29312	4,643736	0,001951
SLC22A4	1,315909	1,540979	0,000419	KCNJ4	-1,21169	0,294842	2,2E-05
PPP1R32	1,110584	1,35065	0,000358	GADD45B	-2,31861	4,473509	1,62E-08
ADAM20P1	1,78971	-0,69904	0,002293	DISP2	-0,78244	0,562624	0,001515
CDC42BPG	1,353456	3,575311	5,42E-12	AURKA	-0,54514	5,912337	2,88E-07
IFI44	1,71863	-1,44132	0,010987	HOXD8	-3,08324	0,704748	4,26E-05
36951	2,191511	-0,39958	0,003003	BICC1	-2,93746	2,229609	3,6E-05
PCDHB5	0,759059	1,855368	0,041939	RHOBTB3	-2,34026	5,981026	1,06E-14
CDK14	1,589627	4,961963	2,21E-12	NKD1	-2,43066	1,330141	1,06E-09
TSC22D1-AS1	1,596329	0,109302	0,000831	TRIM6	-1,00428	4,348856	8,78E-07
KIAA0825	1,492115	1,07756	8,12E-05	PLK2	-1,43693	7,331139	6,99E-12
PCDHGA2	1,469985	0,000472	0,003662	DLC1	-2,00268	1,139564	0,015253
RP11-66N24.4	1,315343	0,292692	0,004513	ZSWIM5	-1,03215	1,169073	9E-06
ZNF619	2,2033	0,228589	0,000477	ADRB1	-1,49523	-1,41551	0,000122
C16orf86	0,613509	1,204464	0,010982	VANGL2	-0,83057	1,581976	3,96E-09
ABTB1	1,291353	4,975664	6,75E-09	USP2	-1,2387	3,529772	7,4E-10
WDR17	0,953473	3,229448	3,29E-10	E2F8	-1,22652	3,659038	6,86E-09

COLQ	0,723033	2,551497	0,00083	RP11-480I12.5	-0,61968	-0,19903	0,004521
RP11-305L7.7	1,220712	-0,66685	0,011717	SNAI2	-0,36216	3,962293	2,91E-06
KLLN	1,886889	0,281787	4,89E-07	CFAP57	-2,67254	0,921506	5,86E-07
ATXN1	1,276186	1,311648	0,032388	SLC39A4	-2,54638	0,963761	0,00022
CCDC144CP	1,251234	0,005149	0,005006	ZNF670	-0,63026	-0,96372	0,021297
PCDHGA11	0,78338	2,254859	9,03E-05	C2CD4C	-0,63441	1,352354	0,000277
CYP46A1	1,313409	0,928597	0,000624	ERCC6L	-0,52534	4,09444	0,001107
RYR1	1,4987	-0,58431	0,000689	FJX1	-0,78985	0,588477	0,000228
PCDHGA7	0,678577	2,669481	0,0016	ZNF724P	-1,82958	3,184196	3,94E-09
LGALS3BP	2,445285	7,548418	3,29E-17	NRARP	-1,9968	1,87808	7,84E-08
RP11-307B6.3	1,910332	0,229256	2,25E-05	CCNA2	-0,53235	6,51979	1,95E-05
AIFM2	1,931827	2,713165	4,51E-07	KLHL29	-2,92975	2,941507	5,61E-11
S100A3	1,3689	2,762846	0,000128	ATAD5	-0,71071	4,165191	7,14E-07
AC008746.12	1,156396	-0,39229	0,0103	CHAC2	-0,56704	3,279102	2,99E-06
PCDHGA6	0,894571	4,598787	8,09E-09	KIAA0101	-0,36398	5,002652	0,004568
SGMS1-AS1	1,0581	2,874772	1,52E-05	CORO1A	-1,71901	1,801571	4,35E-08
NGF	1,315438	1,095822	0,001214	HES4	-2,43572	1,190961	4,61E-06
PINLYP	1,730344	-0,36484	0,010183	RP11-830F9.7	-1,52681	-1,32229	0,012388
RP11-210M15	1,665484	1,983091	4,15E-07	CENPI	-0,75953	4,063504	1,31E-05
FAM198B	1,112946	1,974872	0,042124	TEX15	-0,96988	1,095111	3,83E-09
CALCOCO1	0,92672	5,606411	4,75E-08	RP11-142E9.1	-0,709	1,592724	0,000283
PCDHGA4	1,534497	1,800251	0,000315	CPPED1	-2,32138	3,754651	1,11E-09
GLMP	0,471588	3,353636	4,65E-06	CCDC69	-0,87745	1,232312	0,000106
SATB2	2,271218	3,433426	1,1E-10	KHDRBS3	-2,5099	0,802381	0,000115
PARD6G-AS1	0,645806	0,893825	0,0136	NCAPG	-0,2382	6,208837	0,00154
SDAD1P1	1,067583	1,400034	0,007423	EAF2	-1,62457	0,124944	1,49E-05
CUZD1	1,708105	-1,2345	0,010987	LHFP	-2,72704	3,911552	1,31E-13
PHYKPL	0,803702	5,452566	1,32E-06	PBK	-0,24554	5,914543	0,027008
CASC2	1,206257	2,35607	2,84E-06	CUBN	-2,49827	4,429328	3,02E-17
SH3BGRL2	1,366651	2,974223	2,46E-07	AP000251.3	-0,76092	-1,70601	0,008139
CRIP2	2,014454	6,40444	2E-12	DUSP9	-0,32927	1,291755	0,028228
RP11-66N24.3	1,180453	3,319892	0,000733	STARD8	-1,63166	0,033142	0,001169
PCDHGA10	1,369951	4,263306	2,51E-10	CREB3L1	-0,34366	1,827195	0,004538
HTR7P1	1,825719	4,006003	4,09E-14	METRNL	-1,76598	3,376422	2,14E-07
DPH1	0,627279	4,559866	2,04E-07	KIRREL	-2,42945	8,374762	5,63E-17
DHDDS	2,342726	5,821681	1,29E-16	AFAP1L1	-1,58867	2,212361	1,72E-06
KIAA1324	1,744421	1,384933	1,83E-06	TMOD1	-1,90471	0,206625	0,007064
RAPGEF4	0,633391	1,651758	0,022934	PAG1	-1,29461	-0,48277	1,23E-07
PCDHGB6	0,499381	3,805916	4,45E-05	POLE2	-1,42948	3,578884	3,51E-11
BMF	0,844053	1,484579	0,006306	ARL4D	-1,47602	2,845615	1,11E-06
JUN	1,437988	5,832294	1,24E-10	RHBDL3	-1,60496	2,788508	5,76E-08
IDUA	0,811309	4,705131	1,76E-06	PLK4	-0,59492	5,00307	5,71E-06
PLD3	0,552869	7,137203	5E-08	PIF1	-0,46336	4,114869	0,00025
PCDHGA9	0,911782	1,590246	0,003361	ATAD2	-0,5154	6,739612	1,7E-06
ZMYND10	0,872415	1,346547	0,012905	SAP30	-2,13265	2,166053	2,77E-07
NFKBIZ	0,708048	5,282808	0,001739	LPHN3	-2,16407	-0,34917	0,000269
ADCY5	1,021676	0,574676	4,3E-05	EPHA2	-2,21895	6,083145	1,05E-13
NEK11	1,480322	4,083706	1,61E-10	ATP2A1-AS1	-0,61451	1,183283	0,004419
SEZ6L2	0,269646	5,988719	0,000133	EMILIN2	-2,3727	1,832597	4,31E-05
ZNF596	1,420666	3,367005	4,25E-09	ERBB2	-0,36082	8,04896	2,09E-05

CALCRL	1,516033	3,899379	8,13E-13	CHEK2	-1,48832	3,513354	2,17E-13
C1S	0,732874	4,144652	1,42E-05	GINS4	-0,42383	5,157813	0,000891
KLHL31	1,435881	0,681362	0,000142	ERI1	-1,87166	6,166208	1,47E-15
DNAH1	0,889213	4,21771	1,22E-07	RGL1	-1,92521	1,792738	2,31E-09
MST1P2	1,418903	1,64533	9,13E-07	SHROOM3	-2,07034	4,857996	3,13E-13
MX1	1,04588	2,437718	0,001358	AURKB	-0,93355	5,494527	1,77E-08
PITRM1-AS1	0,911753	0,096932	0,023018	CDCA8	-0,1766	5,694262	0,039118
NEAT1	0,950378	9,579888	3,7E-06	SPARC	-0,47532	6,497022	2E-07
TTC23L	1,294337	0,529649	0,000943	BIRC5	-0,28542	6,64117	0,004046
PBXIP1	0,976749	6,122531	3,8E-10	EFCAB1	-1,06279	-1,31588	0,000214
PTOV1-AS1	1,692118	1,478593	2,87E-06	SCARA3	-0,64556	2,477146	5,7E-06
RP11-344N10.	1,668174	1,280322	2,48E-06	PTRF	-2,19718	7,10197	7,32E-13
DNM1	0,676422	6,954665	3,16E-08	MMP16	-1,32275	1,172521	2,19E-05
MEIS2	0,912514	5,512034	1,39E-10	TTK	-0,34795	5,487946	0,000321
EVA1A	2,147714	1,287906	0,006567	COLGALT2	-0,45953	0,910423	2,91E-05
SPATA20	0,458321	6,575181	4,15E-06	PHOSPHO1	-0,90701	-0,26842	0,002766
NEIL1	1,023504	4,266748	2,76E-08	RRM2	-0,38431	7,68189	0,000975
LRRC66	1,46584	0,884056	0,00016	TBX1	-0,84597	3,585794	1,38E-08
RP11-33O4.2	1,204966	-0,4185	0,016751	ZFP69	-2,16661	2,441454	3,28E-07
SYNE1	1,399815	5,74275	1,76E-09	E2F2	-0,46186	4,549297	1,88E-05
RP11-266K4.1	1,480366	0,108667	2,2E-05	PLEKHA7	-2,04735	2,245876	0,003476
ISYNA1	0,989653	6,712601	1,09E-10	TUBB2B	-1,58397	-2,33425	0,025744
LINC00893	0,636921	2,0147	0,048253	UHRF1	-0,38595	5,064992	0,000576
RP11-54O7.3	0,780872	5,117778	2,57E-05	NTN1	-0,52321	3,211781	7,16E-06
KIAA1244	1,374293	2,773929	0,000184	ARHGEF39	-0,37666	4,225972	0,008479
ULBP2	1,68577	0,520646	1,39E-06	LINGO1	-1,29183	0,591728	4,65E-08
CTD-2026K11.	0,900402	0,380459	0,014636	RP11-462L8.1	-1,57896	-1,2618	0,022254
RP11-83A24.2	1,584162	2,979368	3,49E-08	MYBL1	-0,55029	3,570484	0,049713
AC000120.7	1,152738	-0,39536	0,003723	LA16c-380H5.5	-1,31084	0,556353	0,000955
RP11-1212A22	1,02378	1,477165	0,002299	ASPM	-0,32864	6,642658	0,001986
RP11-432J24.3	1,21503	1,338855	0,000807	SLC35F3	-1,67818	1,402927	3,12E-07
RP1-179N16.6	1,076871	0,841404	0,003729	HES6	-1,84593	2,764665	9,59E-10
ING4	0,66691	4,096039	5,39E-05	RP11-1055B8.4	-1,54674	0,711721	0,000872
FAM49A	2,086298	1,345156	0,001427	BUB1B	-0,46003	6,200117	6,91E-05
RP11-49C9.2	0,768622	0,386887	0,02019	GTSE1	-0,26526	5,268273	0,012742
RHPN1	0,972723	5,740516	5,05E-09	LINC01138	-1,65451	-1,41013	0,034659
BHLHB9	0,448755	3,215344	0,000278	BUB1	-0,52801	6,098354	8,29E-06
CYB561D2	0,911707	-0,05212	0,001158	CHODL	-0,99675	-1,35279	0,005397
VAMP5	1,200088	1,087343	2,85E-05	PLIN2	-0,87997	1,273189	1,58E-07
FBXO41	0,305753	5,319628	0,001134	CDCA3	-0,3553	4,817305	0,000189
DENND6B	0,783569	3,428563	5,55E-06	C16orf59	-0,31419	3,740672	0,0097
LURAP1	1,38862	3,050187	5,94E-08	FMNL1	-2,11953	4,266998	2,46E-13
RP11-49I11.1	0,93981	1,142778	0,004938	STK32B	-1,54863	-0,71686	8,28E-10
PROC	1,71863	-1,70017	0,020846	P2RY1	-0,81213	-0,16801	5,17E-05
SLC38A3	0,301968	1,370405	0,028622	RGS16	-0,38273	0,147269	0,039976
PRRT1	0,411857	3,420036	0,000553	CCND1	-1,02792	7,099922	6,11E-12
NPDC1	0,904903	5,746179	7,4E-10	RIMKLA	-2,42004	2,46124	6,85E-11
RP3-368A4.6	0,570774	3,787475	0,013776	FAM95C	-2,18713	-0,66798	0,01063
PLEKHG5	0,638847	5,819908	1,44E-07	KIFC1	-0,22674	5,877097	0,010943
ANKRD24	1,021307	0,4846	0,002219	RAD51AP1	-0,31759	4,139643	0,002049

DUSP8P5	0,932321	1,722185	0,001586	AC016831.7	-1,91144	-0,86903	0,00094
LRRC16B	0,547623	0,902369	8,44E-05	CRLF1	-0,85106	-0,47673	7,86E-06
ZNF233	0,571615	2,176337	0,006606	CTD-2523D13.	-0,89213	-1,62559	0,021008
MAP3K12	0,457933	6,223994	4E-06	RP11-757F18.5	-1,96976	-1,42184	0,009525
ARHGAP26	1,363285	4,650853	4,72E-11	HLF	-1,09167	-0,04393	0,000458
FERMT3	1,305241	-0,25005	0,010481	UBE2C	-0,587	5,602227	1,3E-05
CXCL16	1,330696	5,089747	3,75E-14	KCNN1	-1,59505	-1,06174	2,91E-08
CTC-559E9.1	0,616674	2,123154	0,004014	ZNF273	-2,09331	2,600248	4,86E-11
RP11-244O19.	1,399985	0,699379	0,000345	CISH	-0,99509	3,433098	8,59E-06
PRRG2	0,826283	0,85973	0,000259	SDK2	-1,81722	0,779049	1,52E-09
AC005154.6	0,430116	5,356649	0,004559	C3orf52	-1,9009	1,465863	5,96E-06
CBLN3	0,572376	1,735422	5,04E-05	MCM10	-0,58987	4,668113	0,000192
SIL1	1,003402	4,898795	4,97E-09	HMMR	-0,1856	5,22083	0,003954
PCDHGC5	0,801457	1,704121	0,002544	ANKRD34B	-1,39103	3,385972	1,42E-09
RP11-631N16.	1,129578	0,892591	0,002758	SDC1	-1,41736	6,330194	1,85E-10
KIAA1875	1,733073	1,630569	4,14E-05	STAC2	-0,88231	0,341958	5,85E-05
VLDLR	1,303644	5,18331	4,18E-10	RP1-193H18.2	-1,06195	1,152194	5,95E-06
KIAA1107	0,594035	2,858909	0,000331	TACC3	-0,3401	6,6133	0,000211
FTX	0,662923	1,955862	0,039666	TROAP	-0,26925	5,869182	0,001321
FGD4	1,611055	4,681061	3,82E-06	ANLN	-0,32346	6,9734	0,013799
RUSC1-AS1	1,067016	1,938948	0,002322	RAB42	-1,14509	-1,64634	0,000157
AQPEP	1,241874	-0,50508	0,00838	CDC45	-0,31649	4,520284	0,015408
CTC-241N9.1	1,293774	1,543473	3,9E-06	PSMC3IP	-0,38367	4,388131	0,006091
37135	0,986808	1,067056	0,001179	CLSPN	-0,36322	5,097829	0,005346
TMPRSS9	1,46638	-1,80231	0,047105	CENPK	-0,25529	4,500662	0,007489
RNF208	0,797309	1,74955	0,001025	RAB32	-1,11468	5,067536	3,22E-08
FAM214A	1,308031	4,480252	2,47E-10	SHCBP1	-0,24863	5,635585	0,022293
PSMD6-AS2	0,676018	1,854904	0,00175	TNFAIP3	-0,5535	1,393565	0,044468
KCND1	1,738903	3,310963	1,95E-09	OSR2	-1,57487	1,879157	2,05E-09
ARHGAP4	1,320966	4,409572	1,91E-12	DMC1	-0,40768	0,109835	0,01053
RIMKLB	1,235162	5,759591	4,32E-11	APC2	-1,60132	1,833529	2,89E-12
ABAT	0,720248	1,964927	1,47E-06	KIF23	-0,21382	6,540557	0,001198
SV2A	0,140997	6,082722	0,01168	LMNB2	-0,69487	7,790151	4,1E-06
KNDC1	1,539161	4,443417	1,24E-11	PLK1	-0,17966	6,405479	0,025939
GPR146	0,891954	0,356149	0,016099	CA2	-0,54749	2,270162	0,00021
OSER1-AS1	0,830357	1,784621	0,000832	CKS2	-0,32199	6,220654	0,010072
PLAC8L1	0,989521	0,287685	0,017234	ESPL1	-0,19153	6,145206	0,024926
LINC01139	0,581422	3,719097	0,001289	ARHGAP19	-0,93345	4,17698	1,85E-10
NICN1	0,651842	4,276952	1,57E-05	RP11-1094M1	-0,44163	3,605827	0,002028
LMNTD2	0,732908	2,638174	2,03E-05	BLM	-0,4208	4,332078	5,75E-05
RP11-545I5.3	1,321434	0,814757	0,000664	ACAT2	-0,42015	6,3748	0,000126
RP5-894A10.2	1,285636	-0,04916	0,007553	NET1	-0,26587	5,344765	0,003675
TFAP2A-AS1	1,527118	1,802889	2,86E-05	KNSTRN	-0,43869	5,460357	1,03E-05
JMJD1C-AS1	1,539619	2,250788	1,89E-10	DBF4B	-0,21554	5,103354	0,011603
CRELD1	0,487409	4,546703	0,002358	TBC1D30	-1,78202	2,555855	2,45E-08
GPR137B	0,765224	3,927846	1,72E-08	CTD-2666L21.	-2,05324	-1,19866	0,007877
KB-1460A1.5	0,78516	2,929997	0,000151	SLC18B1	-1,19605	4,124852	3,8E-10
ACADVL	0,272289	8,872132	0,000389	PDCD6IPP2	-0,5137	0,328839	0,000166
TRIM66	0,969686	5,070929	3,32E-06	GINS1	-0,64091	5,458667	0,000221
RP11-600F24.	1,394584	0,757688	0,000146	HDX	-0,3808	-0,52845	0,009238

RP11-958N24.	0,726385	2,127419	0,001823	CA14	-1,10836	0,49402	1,04E-09
AMT	0,429322	3,908685	0,003925	BAMBI	-0,87499	1,614272	0,023304
GDPD3	0,476721	2,438585	0,024482	ZNF718	-2,04979	2,80379	6,61E-10
LINC01301	1,236368	0,030502	0,018804	JDP2	-1,12252	4,042169	7,56E-09
ITGA7	0,828484	2,724408	0,00106	UBE2SP1	-0,56074	0,203992	0,040612
VASN	0,907844	2,537799	0,002319	CDK1	-0,61581	6,844889	2,66E-08
SNED1	0,519288	4,67612	0,000954	CIT	-0,30282	6,1104	0,004278
MGAT4A	1,102739	3,933031	1,17E-05	KPNA2	-0,41693	8,003207	0,001829
VPS13C	1,386533	5,961116	5,34E-10	NUAK1	-1,69819	4,125702	1,97E-09
BCL2L15	1,129507	1,043109	9,54E-06	MAD2L1	-0,39324	6,163566	0,000394
SLC27A1	0,753885	4,864597	7,87E-07	SGOL2	-0,56394	5,626231	8,07E-08
RP4-694B14.8	1,031984	0,742966	0,005549	APOBEC3B	-0,75369	4,190905	7,96E-08
ABCA17P	0,546346	0,364261	0,036587	SPDL1	-0,15106	6,083268	0,035353
RP11-268G12.	1,695388	0,667957	3E-05	KIF21B	-0,76581	2,683629	0,000237
AMPD2	0,459271	7,506754	1,37E-07	ABCC8	-1,106	-0,07424	1,47E-05
C18orf32	1,380424	2,521535	6,62E-08	MYZAP	-1,58949	-0,51849	0,021576
IL17RC	0,444698	4,876862	0,000148	PLCE1	-0,6095	4,048398	7,13E-05
BACE1	0,982763	5,086178	9,13E-11	CHAF1A	-0,24076	5,816921	0,0292
RP11-48B3.5	1,188062	0,316836	0,009613	CTD-2035E11.	-1,66481	-1,39021	0,027089
LAMA5	0,786167	8,582129	1,24E-09	DEPDC1	-0,25043	5,458239	0,000341
PCDHB17P	0,403348	0,330315	0,033848	GNPNAT1	-1,59592	6,521225	1,36E-15
TGFB1	0,635844	6,732588	7,28E-08	UBE2T	-0,18945	4,861794	0,041953
CFAP70	1,423296	3,361963	8,34E-06	ARHGAP11A	-0,32193	6,344187	0,000136
KCNAB2	0,539059	3,531531	3,71E-06	E2F1	-0,35612	5,403276	0,012857
GSTM2	0,299308	5,281722	0,002321	SPATA5	-1,06653	3,85117	4,55E-09
RP11-531A24.	0,668223	1,360269	0,003331	STRIP2	-1,10385	2,476048	4,89E-06
SIDT2	0,935442	5,816586	7,45E-11	ZNF563	-1,58397	-0,79823	0,036698
JAK3	0,55366	3,438396	1,65E-05	PARPBP	-0,53391	4,648133	1,51E-05
LRRC24	0,545284	2,009678	0,002516	RP11-25K19.1	-1,86274	-1,68634	0,005752
SPRY4-IT1	0,560715	1,067721	0,000102	HELLS	-0,60234	6,028189	4,57E-06
GTF2IRD2B	1,023886	0,374507	0,008156	FAM132B	-0,59698	3,116414	1,14E-05
RSPH1	0,974355	1,765954	0,00018	ORC6	-0,18033	5,020868	0,020714
WEE2-AS1	1,457516	1,067939	0,000171	CASC5	-0,20646	6,334635	0,031605
LENG8	0,253026	8,889691	0,004645	CASZ1	-1,50432	2,188841	7,77E-06
SLC9A7	0,781597	4,744004	8,39E-07	NAGLU	-0,67486	3,902062	9,04E-07
AC156455.1	0,82502	0,36993	0,031786	MAP3K14	-1,65188	5,325839	1,46E-13
ZNF223	0,711985	0,610199	0,012888	RAB34	-0,35929	5,050877	1,57E-05
AMN	1,355049	-0,84049	0,016104	PHF21B	-0,31941	0,119905	0,030852
NEURL2	1,273616	0,94896	6,5E-05	KIF15	-0,16497	5,383572	0,024252
HSF4	0,355353	3,798115	0,037205	HAUS8	-0,41658	4,241507	0,00042
GSDMB	0,4672	5,329422	0,002561	MMD	-1,37566	3,86414	5,61E-11
AC007383.3	1,359498	1,911486	5,1E-07	CTNNAL1	-0,9363	6,125374	2,32E-09
CFAP69	0,693616	2,762774	0,005777	KIAA1551	-0,32259	6,519175	0,001935
GS1-124K5.3	1,06785	-0,0176	0,006744	PKN3	-1,24234	4,723213	1,09E-10
VPS13A	0,397762	7,490324	0,000269	CTB-193M12.5	-0,67522	2,422693	2,61E-05
RP5-1021I20.1	1,558496	0,882441	0,018848	RP11-160O5.1	-0,86683	0,18952	0,001548
RP11-774O3.3	0,684846	1,950119	0,001313	SKA2	-0,38742	6,475661	5,6E-05
TPBG	0,688282	6,131367	1,51E-08	SAPCD2	-0,34603	5,944576	0,002722
RP11-380G5.3	1,0361	-0,15624	0,012239	SPAG5	-0,26174	6,038866	0,002802
PIP5KL1	0,735213	3,133775	1,82E-05	RFC3	-0,19557	5,398663	0,007102

DCBLD2	0,749549	7,491047	1,33E-07	RAD51	-0,38977	4,189209	0,001699
RP5-894A10.6	1,410157	-0,27853	0,00839	DDIAS	-0,39217	4,435197	8,28E-05
AC083843.1	0,979612	2,480131	5,81E-05	RADIL	-1,00427	2,173267	1,46E-07
BCO2	0,667435	1,127263	0,03211	CHMP4C	-1,77703	4,037785	3,18E-13
PLXNA3	0,424812	6,185086	0,000143	ZNF367	-0,3043	4,896522	0,006989
C5orf56	1,448862	1,959553	1,97E-06	PAX8	-1,88951	2,584795	0,016592
PCDHB9	0,388082	2,561398	0,02014	HMGCS1	-0,33522	7,187069	0,005041
ZNF469	0,240105	0,635183	0,023145	NPAS2	-1,25363	4,50549	2,87E-11
MFI2	0,726933	4,344038	8,24E-09	MMP15	-1,68637	4,303713	6,51E-11
FAAH	1,272505	3,337468	4,51E-05	EHD4	-0,65976	5,345658	9,68E-07
UNC5CL	0,41683	2,467205	0,01791	RTN1	-0,26755	1,459095	0,016262
HEBP1	1,713563	6,086158	1,45E-15	POLA2	-0,28003	5,140413	0,007388
FGFRL1	0,235905	5,998387	0,009436	IFFO1	-0,38713	1,139745	0,004953
LRRK2	0,965637	2,209901	0,0034	RAD54L	-0,37746	5,081174	0,000383
NBPF2P	0,607208	0,637919	0,013123	ZNF695	-0,47668	2,427459	0,000107
IFITM10	0,760495	3,208522	6,7E-05	LINC00116	-1,88882	-0,71761	0,017822
NUCB1	0,340456	7,190305	2,44E-05	SOX18	-0,74587	-0,0807	0,000428
VMAC	1,047816	2,783141	0,000115	GDPD1	-1,87143	2,200475	7,64E-10
RSPH3	1,122714	4,742087	1,15E-08	FZD2	-0,36405	4,795042	0,000956
CARF	1,072197	4,267486	4,03E-05	GMNN	-0,61768	5,449205	3,62E-06
DPYSL2	1,45489	7,266538	1,31E-13	HOXC-AS3	-0,5439	-1,21896	0,035756
NIPAL2	0,752891	2,495042	9,82E-06	LRR1	-0,29785	4,292841	0,000373
MESP1	0,803949	-0,28372	0,016065	CEP152	-0,51158	4,751966	1,31E-05
MXD1	1,501343	2,966318	1,29E-07	PDGFC	-1,90047	0,864209	0,004531
FAM227B	0,703233	1,811762	0,00097	SEMA3C	-1,83455	4,400497	1,25E-07
PCDHGA1	0,815355	1,319895	0,020342	HMGB3	-0,56056	6,426304	1,79E-07
AL023806.1	1,034447	-0,29159	0,006677	HS6ST2	-0,40333	0,435801	3,6E-05
ZBTB20	1,308308	1,255615	0,000835	CDKN2D	-0,66316	2,250175	0,028218
VPS9D1	0,576295	3,730105	0,012541	NCAPG2	-0,42655	6,257748	8,12E-05
GLRX	0,469905	3,241543	0,006944	KIF2C	-0,20338	6,51782	0,017753
CCDC114	0,573005	1,508377	0,004264	TYMS	-0,50277	7,326497	9,41E-05
HESX1	1,073025	1,382084	8,48E-06	CENPO	-0,17886	5,586414	0,022697
GOLGA8N	1,140237	0,298182	0,002473	MTBP	-0,48725	3,780236	8,91E-05
MYL5	1,193811	4,103417	1,27E-08	CCRN4L	-0,4397	2,945927	0,004095
AC005618.6	1,152168	0,853744	0,004565	AP4E1	-1,47584	5,167605	3,9E-13
PCNXL2	0,548719	5,117215	0,000563	KIF22	-0,22281	6,55667	0,003372
TAPT1-AS1	1,138699	0,005378	0,011576	NES	-0,70571	6,468323	3,14E-06
FCHSD1	0,275999	5,9737	0,002551	IL18	-1,8527	1,295125	0,018672
ZSWIM8	0,486483	7,034711	9,7E-07	ZWILCH	-0,19122	5,587587	0,03692
TMEM143	0,226196	4,032491	0,018682	DANCR	-0,38879	4,219383	0,000368
PACSIN1	0,858192	-0,37951	0,007776	MYO18A	-0,15206	5,761078	0,047918
ZNF546	0,449754	3,383481	0,002718	CRLF3	-0,63807	4,185204	1,63E-07
AC006946.17	1,000317	0,590964	0,003163	CRY1	-0,9075	5,809875	2,77E-10
MZF1-AS1	0,690382	1,875386	0,011061	MURC	-1,45817	1,087318	6,33E-05
FAM229A	0,50342	4,748219	0,002558	CBFA2T3	-0,96052	2,303505	6,53E-07
RP11-795F19.	0,691023	2,274369	0,000169	ZNF43	-1,79577	1,059339	0,025017
ZNF454	1,022523	0,246344	0,009699	CABYR	-1,84195	2,939762	4,83E-10
PPAP2B	0,649075	4,522697	0,000384	NUDT15	-0,32176	5,006682	0,000622
LINC00174	1,074015	3,806327	1,67E-08	ZC3H15	-1,58333	7,868802	4,69E-15
ADAMTS3	0,406852	3,782337	1,16E-05	NT5C2	-1,32845	6,568568	8,54E-14

PLA2G6	0,620895	4,546445	0,001007	XRCC3	-0,25757	4,317829	0,025745
RP11-196G18.	1,129729	3,604788	4,41E-08	ZNF800	-1,88245	4,062633	7,33E-11
FAM63A	0,761978	4,325118	2,58E-07	MAP2K6	-0,52084	1,876549	0,000522
GPR158	1,521752	2,412479	0,021445	HMHA1	-1,65521	2,929546	7,03E-10
PTEN	1,597776	6,439861	4,79E-14	FADS1	-0,76422	7,339908	6,26E-10
LRRC39	0,647228	0,96247	0,031953	DNAH10	-0,47701	1,298214	0,039086
HBP1	0,726567	5,436197	1,45E-06	ELFN2	-1,72799	2,734771	0,000906
RPS4XP16	0,986924	-0,14206	0,013615	IPO5P1	-1,85306	3,726963	1,91E-10
U47924.31	1,246111	0,162668	0,000351	PSME3	-0,23521	7,262879	0,001406
PLCD1	0,327455	3,530467	0,001819	H2AFZ	-0,5506	8,297869	1,61E-05
FAM174A	1,301529	2,843666	1,34E-08	CGN	-1,07129	3,536954	1,6E-06
MR1	0,820576	3,845174	9,53E-10	RP11-253E3.3	-1,26194	2,876764	5,85E-08
ZNF862	0,907281	4,446048	2,4E-07	GBX2	-0,68255	1,662865	4,32E-05
REEP2	0,377806	5,45149	4,34E-05	TLCD1	-0,46185	2,808294	3,55E-05
CTD-3193O13	0,799664	0,522858	0,005283	HMGB1P5	-0,26708	1,994691	0,018721
HPX	0,528359	1,15401	0,048899	WDR62	-0,39103	5,79099	0,000598
ZBTB8B	1,135115	1,147028	2,17E-07	CHEK1	-0,44798	5,287462	0,000135
CLK1	0,328071	6,038869	0,000162	BORA	-0,29398	4,48281	0,000956
ZNF461	0,699962	3,866838	0,000527	ZBTB18	-1,50833	1,314497	0,007209
TBKBP1	0,460226	5,776323	2,98E-05	MESDC1	-0,90924	4,246387	3,68E-07
CERCAM	0,377386	5,936522	1,02E-05	TNFRSF12A	-0,73281	6,15273	9,27E-07
RP11-15A1.7	0,789385	0,154469	0,013229	PROSER2	-0,75966	3,316779	0,002092
RP11-338N10.	1,281621	-0,2236	0,045523	EZH2	-0,70738	5,747023	4,52E-09
ATP2B4	0,723018	7,09608	1,55E-08	SYP	-0,93613	1,40646	3,43E-06
RALGDS	0,292531	6,35841	0,000679	CDK2	-0,66303	5,990691	6,56E-06
LINC-PINT	0,614968	2,457596	0,000833	NUDT1	-0,3696	3,674004	0,004943
ZNF585B	1,178325	2,795193	0,003203	TPK1	-0,46523	2,986732	0,007063
CBLB	1,05456	6,345512	3,59E-10	CENPW	-0,58999	3,550176	0,009217
BAZ2B	0,820819	6,096514	2,1E-09	CDCA4	-0,28815	4,91107	0,024618
TSPYL2	1,224959	5,743609	2,14E-12	NAGS	-1,07535	0,231769	0,001223
ZNF337-AS1	0,798312	2,990774	0,001536	SAPCD1	-0,54134	0,891818	0,037699
CLK4	0,626876	4,540944	6,91E-06	FOXO1	-1,13132	4,817752	5,55E-11
SH3BP5-AS1	0,684663	4,308396	6,83E-06	SPSB1	-1,32685	3,011747	1,51E-07
PDLIM1	1,250816	4,929112	1,1E-10	CCDC18	-0,82233	4,222948	1,44E-08
RP11-174G6.5	1,331152	2,348718	3,57E-06	OLMALINC	-0,63591	2,810936	8,66E-05
NBPF10	0,715044	1,696619	0,001534	MASTL	-0,32295	5,417072	0,000119
C5orf45	0,423565	5,866242	0,000176	PSRC1	-0,33837	6,330951	0,000817
GPX1	1,437196	5,668012	1,6E-11	FAM60A	-0,8527	5,94897	7,56E-09
PSAP	0,746205	9,952532	2,4E-10	CHORDC1	-1,54525	5,586402	1,72E-12
ABCA2	0,663622	7,068531	7,34E-08	ELOVL1	-1,53521	6,463591	3,83E-13
STC2	0,876768	5,165293	0,000272	CCDC121	-0,42967	2,687864	0,00061
SEC31B	0,932493	4,580768	1,42E-06	HABP4	-1,3172	5,3369	3,11E-10
LENG8-AS1	0,742662	2,000071	0,012579	MT1X	-0,5163	2,851183	0,004439
CCDC30	0,896199	2,452013	0,019489	HOXA1	-1,43224	-0,96364	0,022652
CBR3-AS1	1,075241	0,711322	0,026974	TIMELESS	-0,23695	6,924015	0,017279
LINC00240	1,250706	0,384292	0,001217	CDH18	-1,65451	-0,83175	0,012922
XXbac-BPGBP(	0,642207	0,944481	0,034494	C15orf59	-0,48245	0,444223	0,010987
LRRC56	0,600682	3,648786	0,002974	TIMP2	-1,65465	6,740851	4,82E-13
FAM114A1	0,923755	6,248908	6,7E-10	HYLS1	-0,70451	3,324785	0,000268
TNRC18	1,101011	5,473675	1,2E-09	EBNA1BP2	-1,66821	6,957006	3,43E-14

GATS	0,340053	3,789037	0,006306	BBS7	-1,57761	4,509395	5,69E-12
GBP3	1,250854	3,209613	9,41E-08	DIRAS1	-1,13889	3,625437	1,26E-11
ARMC12	0,545723	0,393135	0,010154	RPP25	-0,34768	3,774028	0,000271
TMPRSS5	0,987333	0,677971	0,003815	RP11-342K6.1	-1,39912	3,593522	3,13E-09
CLSTN3	0,504839	5,832678	6,92E-07	TIPIN	-0,71773	3,835954	3,33E-07
PROS1	0,310924	6,11319	0,001169	ATP8B1	-1,44006	3,236704	0,000107
C5orf66	0,791326	0,205949	0,031034	TUSC1	-1,4142	-0,89098	0,041326
PAXIP1-AS2	1,056721	1,558547	1,34E-06	CYB5B	-0,91723	6,918103	5,1E-09
GET4	0,679609	2,347304	0,004514	NEDD1	-0,87071	5,548657	2,78E-10
KIAA1407	0,747791	4,196683	3,33E-05	RAB3IP	-1,33777	3,605397	0,000189
SPSB3	0,802155	4,459747	1,53E-05	CASC10	-0,77632	4,786272	1,31E-06
RP11-708J19.1	0,890841	0,989926	0,001297	WNT3A	-0,59559	1,160059	0,000111
PCDHB16	0,908287	3,572449	2,1E-06	PLCG2	-1,32335	3,417154	0,000126
RP11-45M22.2	0,737515	1,653092	0,00216	SLC25A19	-0,29912	3,85728	0,003208
GLTSCR2	0,542147	6,876709	3,11E-06	HNRNPC	-0,37217	8,191298	1,66E-05
OGT	0,486957	8,665062	0,000657	CCNH	-0,70167	4,887781	2,49E-06
NNT-AS1	0,579241	3,748915	2,67E-06	FHL2	-0,76854	4,895229	2,76E-05
EVA1B	0,564383	3,322803	0,000714	DNMT1	-0,18277	7,591345	0,048118
TOLLIP-AS1	0,853894	0,144481	0,006284	RP11-293M10	-0,74464	0,049523	0,023442
KCNC4	1,044447	5,579821	4,72E-11	CNN2	-0,62877	7,064468	2,48E-08
PSORS1C1	1,191012	1,154301	0,004536	IDI1	-0,46444	6,656468	0,000209
PNRC1	0,331504	6,08277	0,001019	ZNF669	-0,89714	2,943049	1,12E-05
CAMTA2	0,425127	5,842681	7,63E-05	MIS18A	-0,23583	4,877545	0,010383
RP11-250B2.5	1,017373	-0,22979	0,014419	ZBTB33	-1,67054	5,302685	1,21E-12
CCDC176	0,527897	2,956373	0,02169	UGT2B7	-1,42471	-0,52362	0,043385
KIF9-AS1	0,691443	2,407199	0,00323	PRIM2	-0,30611	4,607773	0,007133
LCA5	0,723345	3,120092	3,03E-05	ARHGAP11B	-0,609	4,866415	1,23E-06
MRC2	0,383833	7,597016	1,57E-06	SH2D5	-1,21979	1,370616	1,23E-07
CTD-2173L22.	1,067602	0,113673	0,003058	TBX2	-0,47593	4,382946	6,3E-07
ZFHX2	0,618925	2,737826	0,000498	AXIN2	-1,30652	1,562576	1,14E-06
DDN	0,807931	2,637949	1,69E-06	APBA1	-0,90138	1,78164	0,022391
ZNF17	0,37352	2,88441	0,015879	CYCS	-0,40474	6,633358	1,24E-05
GPCPD1	0,827373	5,064577	9,49E-05	CDC42EP2	-0,70233	3,325604	8,67E-06
RP11-46A10.5	0,763722	1,269626	0,000623	JUP	-1,27136	5,313661	6,55E-11
STAT2	0,247498	6,570451	0,005326	RNASEH2A	-0,26191	5,490548	0,023442
IRF2BP2	1,457968	7,058455	1,31E-11	PTPRK	-1,4142	0,981454	0,035083
BTG1	1,323709	4,032464	1,6E-06	GNE	-0,91525	6,151391	2,34E-10
PNPLA2	1,1305	6,477419	1,64E-11	RP11-443B20.1	-0,78126	1,929499	6,35E-05
MAPRE3	0,442548	4,717675	0,0003	FAM171A2	-1,24454	3,117724	4,51E-07
ALDH3A2	0,21267	6,924534	0,001198	APBA2	-0,29242	4,5361	0,000128
ATF7IP	1,032357	6,521688	4,3E-10	PRSS16	-0,25108	2,98475	0,045094
AC083843.4	0,990567	0,172575	0,012011	SPRTN	-0,44538	4,23038	2,27E-05
PFKL	0,314463	7,996539	2,55E-05	MICB	-1,09313	4,060409	1,63E-10
ZNF23	0,526709	1,25	0,043699	GALNT12	-1,17782	4,352323	4,72E-12
GULP1	1,467647	6,83869	4,7E-13	SLC35G1	-0,91834	3,646553	1,13E-09
PTPRE	0,787435	4,498043	1,11E-06	TMPO	-0,17129	8,108019	0,012209
TESK2	0,937848	1,719186	0,000242	COTL1	-1,21523	6,446001	2,26E-09
ZBTB4	0,379037	6,501163	1,85E-05	NT5DC1	-1,5178	4,875494	7,79E-11
MDGA2	0,414141	2,315505	3,53E-05	TTC9	-1,18609	2,797233	3,4E-05
NUS1P2	1,147366	2,702214	0,031953	SUZ12	-0,18685	6,609012	0,003012

RP11-159N11.	1,033055	-0,28266	0,020012	MFSD2A	-0,47063	2,000228	0,000293
CH17-472G23	0,697846	1,401431	0,020324	FSTL3	-0,48049	4,990578	0,000916
FUOM	0,941537	3,792064	5,15E-09	COQ2	-0,72469	4,491184	9,48E-06
MAMDC4	0,74828	4,471083	1,8E-06	TUBB2A	-0,91765	3,045135	0,001656
FAM89B	1,039058	2,910698	8,89E-07	SLC2A12	-1,27207	0,798412	0,000421
ARRDC2	0,710453	4,711904	1,56E-05	NUDCD1	-0,80851	5,310466	6,94E-09
CSAD	1,131956	5,559026	2,03E-08	PSMD11	-0,49548	7,06702	6,15E-06
ACP6	0,835784	4,759212	8,34E-10	CCDC167	-0,82304	3,245043	0,000458
CECR7	0,858857	3,909104	1,41E-08	ABCG4	-1,30592	1,911356	4,59E-07
EIF4E3	1,225418	3,079401	1,42E-07	ALDH1B1	-0,25106	5,818679	0,001792
CFAP53	1,057585	1,581799	0,000318	PLCH1	-0,84377	2,063118	0,00682
SLC25A37	0,255252	7,201513	0,0017	RP11-73E17.2	-0,7638	1,136378	0,000535
PTMAP4	1,283928	3,803613	3,49E-09	SLC4A8	-0,53901	3,610208	0,014255
ENOSF1	0,341128	6,703709	0,002569	RANGAP1	-0,312	6,482376	0,000448
ARHGEF1	0,319579	5,972286	0,000447	IDH2	-0,46049	5,469865	1,46E-05
PPAPDC3	0,350238	-0,26611	0,005383	MYO19	-0,27368	6,651879	0,00161
RP4-800G7.2	0,647293	2,975055	0,001058	EXOSC3	-0,49504	5,01842	3,72E-06
RP11-66D17.5	0,660593	0,410016	0,029101	CKS1B	-0,30131	4,888481	0,001626
SLCO3A1	1,199321	3,730542	1,49E-07	PKDCC	-0,83977	3,18258	1,32E-06
DNAJC12	0,639081	2,316879	0,001838	RP11-298C3.2	-0,52962	0,45674	0,042792
APOL6	1,244327	2,598337	0,007748	CYTH3	-0,84099	4,92926	2,5E-07
ZNF226	0,342833	5,104359	0,002469	PIR	-0,37831	4,147914	0,000665
TNFRSF25	0,265327	4,663891	0,007262	ENC1	-0,87602	4,213843	1,96E-05
PTK2B	0,711387	3,27639	4,29E-05	TUBB	-0,3379	10,11779	0,011494
ZNF497	0,714086	1,878643	0,007056	TMEM99	-0,76068	3,329232	7,85E-05
CCPG1	1,008225	3,830977	7,1E-06	CPE	-0,19209	3,124858	0,021445
TMEM59	0,481969	6,754172	7,93E-08	RPIA	-0,65204	5,081449	1,54E-08
WDR6	0,190828	7,50495	0,013384	COASY	-0,39529	6,012224	3,96E-05
CROCC	0,391828	5,686713	0,000144	COA7	-0,37061	5,789668	0,000194
ZNF425	0,470191	2,350832	0,00311	HMGA1	-0,81151	8,136461	8,03E-10
FER	1,071267	5,810858	4,83E-10	FDPS	-0,52323	7,436361	2,17E-06
RP11-157P1.4	1,226348	1,889449	0,001738	RAB11FIP1	-0,24898	6,248234	0,001437
STMN3	1,166593	3,845049	4,25E-11	TNFRSF21	-0,63201	4,311813	5,3E-05
KIAA0355	0,861003	6,200871	1,73E-09	DUSP3	-0,43455	6,048662	3,49E-06
FAM13A	0,154724	4,146315	0,033045	ZIC5	-1,42471	-0,03563	0,018414
SSBP2	0,795184	5,098952	1,1E-08	GGCT	-0,33184	4,51425	0,000145
CFP	0,693969	1,399619	0,00453	C15orf39	-0,36346	4,607143	0,001679
RBM5	0,418125	7,04158	2,26E-06	LYAR	-0,22772	5,034351	0,007432
METTL20	1,354842	2,702029	6,73E-07	S1PR2	-0,82496	2,952129	2,93E-07
DZANK1	0,34037	3,294759	0,040986	HIP1	-0,59343	5,395206	1,26E-07
EPHA10	0,84708	3,665606	0,001383	MTND1P23	-0,35344	4,126878	0,000939
PC	0,582155	4,55066	0,000124	WWC2	-0,90856	6,629144	3,57E-10
MAPK8IP3	0,181919	7,118468	0,045488	SP6	-0,36824	2,500042	0,001521
PPM1M	0,806941	3,672698	3,34E-08	LSM12	-0,55201	3,422357	3,28E-05
CMTM1	1,180978	1,022892	3,09E-05	GPC2	-0,64433	2,082751	3,44E-05
IFT140	0,25534	5,326805	0,021324	KLHL23	-1,37584	5,545826	5,05E-13
C16orf96	0,501133	0,738372	0,048992	MAFF	-0,89468	3,337454	0,000108
RP11-609N14.	0,912785	1,337311	0,000314	IQCD	-0,70457	1,552473	0,014259
ZNF211	0,271391	4,604137	0,006193	TUBA1C	-0,28958	7,770062	0,00898
CTC-360G5.9	0,625692	1,859133	0,016124	SENP1	-0,72947	5,417359	1,93E-08

HSPA1L	0,694196	0,515667	0,045849	TBC1D31	-0,344	4,62942	0,00058
TMEM134	0,455418	4,503833	0,000464	BPGM	-1,12223	3,403505	3,1E-09
GPI	0,245131	8,684243	0,00381	EIF2B3	-0,84239	4,472388	1,55E-08
NDFIP1	1,024915	7,159832	1,16E-12	HN1	-0,30008	7,383761	0,000269
LSMEM1	0,949153	1,110912	0,001929	TAF1B	-1,14661	4,492076	7,71E-12
C3orf62	0,740429	4,356843	7,44E-07	MTFR1	-0,36543	5,557907	9,64E-06
SHISA5	0,74402	7,157189	3,88E-10	DCPS	-0,66129	4,611946	8,51E-08
PNPLA4	0,679109	3,965418	4,05E-05	PGAM5	-0,30897	6,139608	0,000409
GLUD1P3	0,707681	2,007426	0,00497	CCDC43	-0,38288	5,410356	4,07E-06
RIBC1	0,886445	1,836307	5,85E-05	ARG2	-1,19391	2,73655	1,55E-08
BSDC1	0,689085	6,540493	5,04E-08	NDC1	-0,24217	6,702586	0,004618
SAT1	0,574514	4,773495	0,000394	CDK5R1	-0,51654	3,166337	0,001636
ADRBK1	0,496944	6,853102	2,57E-07	EMC8	-1,03453	5,038561	1,72E-09
AC006946.16	0,67089	3,084814	2,24E-05	AKNA	-0,7623	3,799204	0,000584
MYOM1	0,832665	1,504521	0,00015	NUP35	-0,64067	4,429225	1,27E-07
RAP1GAP2	0,362524	5,345152	6,45E-05	PPIL1	-0,59499	5,561506	7,46E-08
GLB1L2	0,6877	4,939545	2,28E-07	RNF157	-0,53054	4,860117	9,5E-07
OVGP1	0,449109	3,279117	0,005081	SORL1	-0,35234	5,626321	0,000624
MAP4K1	0,781174	1,322078	0,000993	SOWAHC	-0,92618	5,643568	1,54E-10
ASPRV1	0,725955	1,639861	0,000955	C9orf40	-0,2415	4,257185	0,029363
GPLD1	0,43675	2,565878	0,012734	LASP1	-0,2622	8,30387	0,001331
SP140L	1,060702	4,969343	4,61E-10	PAQR4	-0,20472	5,226434	0,018498
LINC01355	0,668934	3,765367	0,001928	POLE4	-0,6087	4,053148	9,54E-05
TSHZ1	0,73127	5,532724	6,8E-09	SNX10	-1,00255	4,815108	6,85E-11
RGL3	0,298689	5,403308	0,007673	TLE1	-0,29877	5,732097	0,000431
IQSEC2	0,259917	5,496527	0,001454	RCOR2	-1,10737	3,702714	1,59E-11
PCBP4	0,457619	5,349995	1,6E-06	MRPL1	-0,24338	4,767741	0,008802
CAPS	0,775171	3,861609	0,001325	GKAP1	-1,32339	3,363687	7,43E-05
CTD-2020K17.	0,695499	2,483657	0,00133	TLN2	-0,39204	4,670422	8,63E-05
SLC35E2	0,687667	1,356124	0,027986	MAGIX	-0,85305	0,284244	0,006875
ZNF302	0,670468	6,249592	8,91E-08	PKP4	-0,70257	6,794674	2,08E-09
PABPC1L	0,542438	6,73755	0,000478	SWAP70	-1,16251	6,317192	1,07E-12
LRGUK	0,915812	0,594142	0,010725	SRF	-0,85928	5,298725	1,69E-09
KATNAL2	0,804079	1,665759	8,77E-06	RP11-498C9.1	-0,80071	3,2418	9,63E-05
ZBTB47	0,894359	4,432249	4,04E-08	HDAC7	-1,22725	7,574457	8,06E-13
INHA	1,061019	1,476966	9,81E-06	GRIK4	-0,60736	0,145667	0,013488
RGL2	0,534197	6,700433	7,8E-07	SMIM13	-0,65486	5,296751	2,3E-08
MAP1A	1,015662	4,022089	2,11E-09	FBXO27	-0,90744	1,876149	9,46E-08
TCTN1	0,789642	5,332487	1,53E-08	DCK	-0,38407	5,236942	0,002207
KIF9	0,757496	2,89352	1,95E-05	FAM57B	-0,32402	1,752466	0,026041
AC009120.6	0,463272	4,383934	0,001992	TUBD1	-1,08634	4,132684	4,31E-09
OSBPL7	0,501907	5,214782	6,49E-05	PLGRKT	-0,40283	3,870098	0,000689
ANKAR	0,498727	2,542931	0,001929	PLEKHF2	-1,02801	3,426609	0,000427
ALDH1L2	0,259015	4,10804	0,002152	SNRPG	-0,63501	5,635443	3,45E-06
CCDC150P1	0,51583	0,461838	0,021948	RP11-159D12.	-1,00491	6,333962	3,49E-08
KIAA1147	1,157349	4,538765	9,12E-08	TNFAIP1	-0,17283	5,283996	0,021553
PCDHGC3	0,821651	2,495868	0,048422	ABHD15	-0,32041	3,360245	0,003843
HEMK1	0,532555	5,288105	8,16E-05	DYRK2	-0,28259	6,185576	0,000866
PINK1	0,735529	5,791211	1,2E-07	MCM6	-0,24279	7,320464	0,041645
USP4	0,71641	5,431816	4,55E-09	YWHAH	-0,49432	6,653471	1,1E-05

LMBRD1	0,941683	4,443026	4,26E-06	DTYMK	-0,33229	5,867587	0,001768
SLU7	0,8864	5,860989	3,59E-10	CD83	-0,29372	2,630749	0,005339
RP11-967K21.	1,16347	1,528466	0,000796	MLXIPL	-0,64172	3,368878	1,68E-06
LPP-AS2	1,035511	2,370769	3,46E-05	BEND3	-0,46516	3,709511	0,000229
ZNF197	1,07944	4,326326	2,57E-08	PRIM1	-0,20631	4,622798	0,030848
TMEM151A	1,146953	1,630193	0,000317	ZBTB5	-0,99885	5,485286	5,46E-11
CASP9	0,561276	4,80342	9,91E-06	SNRNP40	-0,64508	6,193343	3,15E-07
IGF1R	1,259377	6,026562	7,97E-12	AC005682.5	-0,94185	1,031775	0,000258
INTS6-AS1	0,802842	1,51846	0,004764	POLR1E	-0,27357	5,326099	0,000352
RSRP1	0,410046	6,832114	0,000906	PHF5A	-0,41368	4,956157	3,77E-05
LYST	0,808318	4,792409	1,58E-07	RHOF	-0,39792	5,456026	7,82E-05
LINC01534	0,994751	0,621774	0,00911	LIN52	-0,38678	3,1563	0,018525
CHCHD10	0,283667	2,568639	0,002099	DCTPP1	-0,4085	5,486664	7,47E-05
TRIM52	1,122316	5,12971	5,69E-08	SRGAP1	-0,82451	5,374729	1,54E-06
PBLD	1,169939	3,159888	6,67E-06	ZNF267	-0,89722	4,068409	7,24E-10
FBXL8	0,41142	2,773371	0,009221	NUP107	-0,34974	6,712998	2,92E-05
TENM3	0,513473	6,483024	5,75E-06	AFAP1	-1,21488	5,197867	3,07E-08
ZBED8	0,798846	2,817942	0,000645	ABHD11	-0,29127	4,232985	0,002341
RP11-98D18.9	0,793886	0,14832	0,012476	IFI35	-0,45732	4,133377	0,000349
CD151	0,506916	7,559051	5,44E-07	ATP6V1E2	-0,38054	4,040449	0,000147
PTGFRN	0,426271	7,144894	1,85E-05	TP53I11	-0,25116	4,269975	0,002231
ZNF446	0,708699	3,667852	6,56E-05	PCNA	-0,35137	7,463832	0,024049
CRYL1	0,671319	4,304301	2,41E-05	RHOV	-0,99874	1,813231	0,000247
UBXN11	0,635441	6,219447	2,09E-07	RP11-696N14.	-0,58477	1,29112	0,015946
MYO6	1,324261	6,082228	2,97E-12	HAT1	-0,90025	6,9728	8,03E-08
GS1-124K5.2	0,716319	0,662459	0,012943	UCHL3	-0,94201	2,274847	1,87E-05
NBPF12	0,635315	4,327037	4,51E-06	ZNF618	-1,06025	5,856424	2,86E-10
RP5-902P8.10	0,726826	0,478515	0,011513	AMN1	-1,19342	3,992433	1,05E-07
TTLL11	0,755656	1,65921	0,000769	CBX5	-0,55571	8,058308	1,12E-05
ALDH6A1	1,156285	3,575465	2,12E-07	ANKS6	-0,8646	6,552446	1,91E-10
PARD6B	0,366444	4,980242	0,001731	RANBP1	-0,32918	6,537122	0,000946
ZNF571	0,539569	3,80667	0,007913	FEM1A	-0,12569	5,418755	0,048495
FOS	0,526858	5,603705	0,00848	IRS1	-0,27035	4,811455	0,003675
GPRASP2	0,534614	3,499707	4,06E-05	XRCC6P2	-0,70654	0,087479	0,020179
MEGF8	0,622834	6,24766	6,81E-07	MEX3D	-0,28849	5,342823	0,038257
VAMP1	0,634367	4,364628	5,86E-05	RUNDC1	-0,52367	5,018362	1,73E-06
ARNTL	1,211347	3,3888	5,4E-06	GYG1	-0,94057	5,751763	4,86E-11
SPRY4	0,242711	6,886945	0,006112	SNRPF	-0,35097	6,339443	0,000311
UBE2L6	0,550909	5,180909	5,2E-06	FBXL16	-0,69483	4,021471	1,34E-06
ZNF580	0,805703	4,787571	1,51E-06	SLC25A30	-0,41283	5,26167	1,52E-05
TMEM198B	0,589355	4,791987	0,0004	CORO2A	-1,17966	4,640683	4,44E-10
PRSS27	0,7057	1,63387	0,00955	TYRO3	-0,55793	4,696211	4,81E-06
MOK	0,34419	4,358362	0,000928	ABHD3	-0,42683	4,915233	5,03E-05
C9orf172	0,676976	3,387323	0,000102	SPPL2A	-0,78173	6,236976	1,16E-09
BBX	1,285255	7,17329	2,97E-12	EXOSC8	-0,52564	5,710744	1,11E-06
ZER1	0,733553	5,785013	7,33E-09	CDR2	-0,88935	5,212941	8,72E-09
IL18BP	0,543444	3,88445	0,000716	MITD1	-0,9352	5,214336	1,05E-10
NEK10	0,683713	0,919954	0,007756	KNOP1	-0,98982	5,406291	2,31E-11
FAM102A	0,968037	5,563992	2,89E-10	CEP78	-0,19562	6,700274	0,015957
PPP3CA	0,855008	5,53957	6,39E-09	CDCA7	-0,53651	6,363369	2,62E-06

ECHS1	1,253625	7,252064	7,09E-13	CHCHD3	-0,28108	5,889619	0,000473
CTD-2619J13.1	0,581066	3,697953	0,000226	PET117	-0,90931	1,006225	0,001373
TLE3	0,395485	5,239773	6,53E-05	NRM	-0,40857	4,580815	0,001507
TFEB	0,501358	3,593268	0,010789	PINX1	-0,30545	2,972838	0,021233
ITGB8	0,696544	7,109851	1,7E-08	C18orf54	-0,72292	4,847663	1,22E-07
ZNF621	0,505303	6,431875	0,000159	KDM4D	-0,61047	1,417539	0,003785
PJA2	0,604714	7,220423	1,06E-08	LMX1B	-0,87201	2,719207	1,1E-08
INPP5K	0,707602	4,061634	2,17E-07	RP11-617F23.1	-1,01828	1,790002	5,52E-05
ENGASE	0,497261	5,888342	8,86E-06	PDE7A	-0,33327	5,275717	0,002343
MIR647	0,859616	0,316033	0,043235	PTPRJ	-0,76109	5,366028	2E-08
CYFIP2	1,118402	5,468447	4,19E-10	E2F5	-0,40028	4,559933	0,000159
VAMP2	0,248111	4,994989	0,034975	LSM8	-0,29674	5,005285	0,000689
TRIM38	1,113371	6,235959	6,96E-12	MMS22L	-0,28894	5,52133	0,002072
LINC00852	0,691804	1,171692	0,006404	TEF	-1,12855	4,019467	3,53E-07
B3GALNT1	0,755745	4,77998	3,78E-08	BASP1	-1,0686	4,266755	0,000682
DNAJC18	0,341556	3,764746	0,020236	DLGAP1-AS2	-0,4753	3,645598	0,024704
JUNB	0,476311	6,495997	4,82E-05	SKP2	-0,2693	5,750807	0,03822
WASH2P	0,359053	1,978516	0,038024	PAICS	-0,42589	8,02325	5,85E-05
LRSAM1	0,209621	5,212534	0,020729	FANCC	-0,53837	5,205117	3,93E-07
ACADS	0,421713	3,519841	0,007475	MIR621	-0,57278	0,683834	0,007327
ZFYVE27	0,990197	5,16616	1,89E-09	CBX2	-0,32884	4,85574	0,00131
PTPN13	0,777096	6,872412	4,51E-08	ANP32E	-0,38448	7,958652	4,19E-05
CCDC80	0,239668	5,521625	0,01535	DNMT3B	-1,00078	5,783381	1,61E-08
ZNF445	0,599595	6,09175	3,23E-06	AP3B1	-0,55959	6,154542	2,17E-08
NEK7	0,367002	6,716874	1,33E-05	CNTRL	-0,60306	5,133852	7,09E-07
GBAP1	0,354814	3,581118	0,005775	STRBP	-0,47155	5,773356	4,21E-06
INTS3	0,234641	7,758938	0,006092	SLC2A6	-0,69578	3,590881	2,15E-05
ITPR1	0,405619	3,346808	0,000491	MLX	-0,47135	5,429629	4,21E-06
MT-RNR1	0,293786	10,33569	0,002595	ELOVL5	-0,27105	7,671824	0,001069
DALRD3	0,476162	5,183925	4,01E-05	RP11-298I3.4	-0,93929	0,279103	0,025084
WDR19	0,481843	5,632548	2,55E-05	RAB5C	-0,24176	5,606227	0,028143
TPRG1L	0,544842	5,205844	8,96E-06	LSM6	-0,55878	4,504874	5,25E-06
FUCA1	0,462117	4,504499	3,38E-05	TAX1BP1	-1,01278	6,740777	3,4E-12
BTAF1	0,184524	7,242257	0,049113	CHAF1B	-0,55522	5,4586	4,27E-05
KDM3A	0,237953	6,850949	0,001005	NPR3	-1,08578	2,590586	0,002199
FAM210B	0,362197	5,880747	1,57E-05	B3GNT2	-0,84493	4,682243	9,43E-10
LAG3	0,490449	1,166643	0,043169	POLR3K	-0,47727	4,686014	0,001555
GRIK5	0,454238	0,469204	0,042497	SMC6	-0,53783	6,436101	1,64E-08
RP11-5C23.1	0,460553	0,88083	0,025538	RUSC2	-0,97751	5,68304	5,92E-08
STX16	0,318154	7,066947	0,004754	MVD	-0,5386	5,380077	0,000179
C1orf56	0,607204	2,027437	0,000917	LIG3	-0,50098	5,318917	5,61E-07
ZMIZ1	0,825469	7,183372	5,66E-10	SGTA	-0,47387	6,442022	3,88E-06
ZDHHC2	1,092015	6,088629	1,1E-10	IRAK1	-0,27596	7,341237	0,001306
KANK2	0,908345	7,702211	2,67E-10	SPIRE2	-0,44635	3,849121	0,000104
ZNF224	0,318629	5,737767	0,004961	ENY2	-0,66941	6,101195	3,38E-07
MFSD6	0,929317	3,080946	0,0008	LRRC8C	-1,12082	4,207138	1,52E-09
DLGAP4	0,755766	6,187567	4,19E-09	TDG	-0,46813	4,835795	3,17E-06
KLHL24	0,988526	5,399294	4,59E-07	TXNDC9	-0,64615	5,088442	1,96E-08
APBB3	0,737535	5,111836	0,000177	HDGFRP2	-0,71227	6,034603	1,02E-07
PPL	0,873896	3,290025	0,000296	PPP3R1	-0,6372	6,46042	3,93E-09

ERCC1	0,206132	6,564609	0,004067	DEPDC7	-0,76537	3,160502	4,18E-05
SLC38A2	0,529278	9,612929	5,9E-07	POLR3G	-0,99479	3,278947	1,24E-07
MMAA	0,855305	3,305608	6,69E-07	SLC20A1	-0,31694	7,215907	0,000591
ROM1	0,802508	1,672438	0,001012	RBBP8	-0,39551	6,45112	2,37E-05
ZNF222	0,496847	2,684409	0,00592	CKAP5	-0,20741	7,859	0,004407
MIB2	0,375755	5,393156	0,002715	SNHG3	-0,31232	6,642208	0,000821
APLP2	1,148393	9,334252	2,1E-13	MREG	-0,43858	3,539718	0,000396
RP11-480A16.	0,843073	1,883535	0,001676	HNRNPUL2	-0,35722	6,011167	0,000214
TSNARE1	0,509666	3,555019	0,000753	SUPT16H	-0,28834	7,730944	0,00078
USP51	0,792594	1,582788	1,46E-05	SGTB	-0,9666	4,224598	9,45E-07
PLXNB1	1,120883	6,689361	5,42E-10	MRPL54	-0,37215	4,191076	0,000721
MBOAT7	0,326904	7,232102	0,000116	ACTL6A	-0,20915	6,667729	0,006989
SLC12A9	0,923277	5,190883	3,11E-10	ERMP1	-1,21429	3,984584	2,53E-07
RP11-196G18.	0,876679	1,73215	0,000289	HOMEZ	-0,36118	3,098709	0,002333
FAM8A1	0,787424	5,767229	1,03E-09	TMEM177	-0,49068	4,398132	1,31E-05
U73166.2	0,693446	1,044613	0,001253	APPL2	-0,94812	5,717503	6,33E-11
ZFYVE1	0,470805	4,536994	0,000182	RFK	-0,3138	5,556097	0,000107
SASH1	0,607792	5,458146	6,44E-06	PDCD10	-0,51802	5,762523	2,78E-07
ZNF837	0,510679	1,046317	0,041399	RAB3IL1	-0,45161	3,465619	0,002056
PCDHB14	0,395521	4,318245	0,001996	ACYP1	-0,42251	3,003225	0,037974
EHBP1L1	0,661875	7,361483	2,94E-09	TMEM169	-0,96609	1,868251	3,57E-06
SPATA7	0,759699	2,406212	0,011485	HIF1A	-1,04509	7,906585	1,69E-11
AP001062.7	0,728578	1,775185	0,011551	NUP93	-0,5463	6,477162	3,01E-07
ZNF805	0,665914	2,200707	0,00254	HPS3	-0,78503	6,226128	7,4E-10
H1F0	0,134893	5,644154	0,016653	FAM107B	-0,42877	4,417684	0,000212
DPY19L3	0,249094	6,100331	0,004079	NEURL1B	-0,39021	3,235213	0,002542
GSTM4	0,527464	5,775858	2,23E-08	TUBGCP3	-0,56762	5,222588	3,41E-07
MVP	0,572198	6,124734	2,5E-05	SLC44A1	-1,0064	7,797333	3,43E-12
ZNF549	0,618604	5,051793	7,92E-06	DEK	-0,24029	8,411901	0,001502
ZNF514	0,473875	5,238693	0,004379	TAX1BP3	-0,83487	2,46693	4,16E-05
HYPK	0,489613	2,350211	0,015281	COMMD2	-0,4836	5,554937	1,17E-06
TBC1D17	0,218952	5,844109	0,012142	PPAT	-0,49666	5,655819	1,64E-07
SH3BGRL3	0,520153	7,082094	3,02E-06	DHCR7	-0,3505	6,233543	0,000302
SUV420H1	0,245239	6,121554	0,022783	CCDC94	-0,41089	3,881544	0,001168
PTPRM	0,31582	5,672455	0,003208	FAM105A	-0,76714	1,524939	0,016419
PRRT3	0,679819	2,999023	1,51E-06	AADAT	-0,95101	4,623215	3,57E-09
RP11-299J3.8	0,808926	1,5737	0,006067	TEAD4	-0,69027	4,766951	7,15E-08
SH3YL1	0,32328	4,47826	0,003756	WHSC1	-0,5102	7,710212	1,2E-06
TTLL3	0,558737	6,999417	6,18E-06	SLC25A51	-0,40207	3,806695	0,000778
PNPLA7	0,519164	2,886406	0,005286	COA3	-0,27885	4,860112	0,010964
CTD-2270P14.	0,483723	4,371998	0,005214	SERPINB1	-0,91974	5,393138	1,53E-08
INTU	0,540686	4,654819	0,000372	MFHAS1	-0,41858	5,671143	1,94E-05
NOS3	0,328623	2,749231	0,000132	LRRC59	-0,21417	8,031028	0,030656
TMEM175	0,825192	4,168543	4,4E-09	NOP58	-0,28397	7,188437	0,000184
ZSCAN30	0,235633	5,727609	0,016495	LIMK2	-0,37334	4,575724	0,000345
ICAM5	0,470352	4,68122	0,000357	POC1B	-0,78012	4,186267	1,54E-06
ORAI3	0,711749	3,030234	0,000112	MTL5	-0,24899	2,539189	0,030803
RP11-226L15.	0,907845	2,184758	0,001111	SRSF3	-0,29616	8,492733	3,48E-05
ALOX12-AS1	0,407818	2,136413	0,024309	SDC4	-1,13632	5,624213	2,81E-09
SP100	0,464831	5,391577	2E-05	ELAVL1	-0,33291	6,899845	0,000271

RAD9B	0,905828	0,966569	0,003365	ADAM9	-0,77894	7,832983	5,39E-10
FOXO4	0,643331	3,33247	0,001221	ATF5	-0,67586	5,549379	8,44E-08
PIDD1	0,949075	5,670159	9,48E-09	LATS2	-0,90189	5,502362	3,67E-09
C9orf9	0,739963	3,042277	0,001348	NIP7	-0,50454	5,551609	7,61E-07
ACAP3	0,411609	6,145262	0,000558	ARHGAP27	-0,46948	4,797803	1,34E-05
IFNGR2	0,507489	6,121515	5,92E-08	ORC3	-0,3498	5,463021	2,35E-05
FAM160A2	0,342186	5,141274	0,005286	STARD4	-0,33724	6,181002	3,13E-05
ZFHX3	0,950783	6,766536	2,79E-10	PPCDC	-0,37861	2,751063	0,01343
BTN3A3	0,478273	3,555946	0,010459	NFKBIE	-0,74653	2,827712	9,36E-05
HCFC2	0,530953	4,063055	0,000311	TAF9	-0,16937	6,138956	0,030148
DFNB59	0,916187	2,494208	0,000125	CNTLN	-0,99174	4,857709	9,61E-10
RP11-440L14.	0,491688	2,123416	0,028455	MRPS17	-0,32272	2,368121	0,031844
TMEM50B	0,601276	6,192406	8,04E-08	ZNF77	-0,68695	3,166516	7,69E-05
LYRM9	0,446387	2,445066	0,014637	RBL1	-0,32204	4,953146	0,003247
RP5-1074L1.4	1,017001	4,975529	4,21E-09	LINC00152	-0,84318	4,089899	2,85E-08
ACBD4	0,710089	4,103559	1,86E-05	RPF2	-0,3692	5,23847	8,35E-05
ZNF419	0,565368	4,954965	0,003372	TMEM106C	-0,34862	6,06199	0,000266
CYLD	0,950146	5,281931	1,44E-08	SAAL1	-0,36886	4,69543	0,000543
SDHAP3	0,692265	2,017687	0,000385	MPLKIP	-0,79257	4,545944	3,54E-07
NBPF3	0,602177	4,175093	2,7E-05	PRPS1	-0,32532	6,145645	0,000868
RP11-87H9.4	0,536443	1,665534	0,037111	CHRNA5	-0,39601	3,599206	0,001254
NBPF11	0,326811	4,216241	0,004324	MCUR1	-0,37644	5,804671	1,45E-05
BHLHE40	0,497027	6,939713	1,59E-06	KIAA0020	-0,31675	5,198565	0,000116
ZNF283	0,549097	4,864912	0,00056	AMD1	-0,48186	6,405663	1,24E-06
ADCK3	1,017558	5,211468	6,33E-11	NMT1	-0,18612	7,135854	0,021268
PGAP1	0,191237	5,110378	0,040995	FSTL1	-0,8828	7,804253	3,73E-09
HNRNPU-AS1	0,939568	6,019061	3,96E-09	WDR91	-0,23468	5,03878	0,020656
RIN1	0,665907	5,323591	1,15E-08	EFR3B	-0,65024	3,605506	1,28E-07
ANKRD13D	0,163328	5,900564	0,021804	PMEPA1	-0,24976	4,407175	0,029958
CCNDBP1	0,356832	5,357902	0,000318	FRMD6	-0,39454	4,203918	0,00059
C1QTNF3	0,630587	1,982518	0,008137	TPRKB	-0,38259	5,199803	8,84E-06
ATN1	0,597537	7,828727	1,93E-07	MTCL1	-0,80614	5,362034	1,96E-07
MSL3	0,78167	5,262097	1,55E-08	MRPS23	-0,59504	6,32248	7,42E-09
RP5-1125A11.	0,766837	1,391811	0,000473	TSSC1	-0,44112	3,916219	9,4E-05
LAMC1	0,768842	8,46782	5,3E-10	GPN3	-0,2271	4,521501	0,020763
ACAA1	0,246794	5,198371	0,001726	RGS3	-0,41756	5,888507	1,49E-05
ADAM8	0,700158	3,706837	9,04E-06	BCL2L1	-0,51005	6,424485	6,47E-07
RP5-1065J22.8	0,822641	3,210312	6,51E-08	WDR43	-0,33908	6,923086	4,58E-05
SESN1	0,78025	4,063007	1,07E-06	RCC1	-0,2804	7,065811	0,00252
C14orf79	0,885293	3,567844	0,001043	C12orf29	-1,01908	4,544507	1,51E-07
CCDC40	0,77189	4,333714	4,14E-05	EXOSC9	-0,30179	5,86385	5,35E-05
SLC6A8	0,337838	6,587962	5,61E-05	CDKAL1	-0,43172	4,528523	3,49E-05
RAB13	0,377707	6,731377	8,83E-05	CDK4	-0,20729	7,126799	0,003066
DENND4C	0,680788	5,640872	4,58E-07	GPN1	-0,64634	5,611341	7,12E-09
ANKHD1-EIF4E	0,543282	1,624467	0,033687	NUP37	-0,61921	5,389041	6,36E-06
ZNF548	0,448158	3,936199	0,001919	C1orf112	-0,28501	4,733264	0,001861
CCDC186	0,390667	4,965547	0,000972	RP11-400F19.6	-0,30518	4,238736	0,000769
PDLIM7	0,244428	6,973214	0,00267	C16orf74	-0,86445	2,910231	9,24E-06
HS3ST3B1	0,978842	2,097269	0,024586	ABHD13	-1,06564	3,991049	7,61E-07
CDK18	0,871604	4,073727	1,1E-10	CCDC138	-0,43928	5,24008	2,54E-06

IER5L	0,775369	5,854012	2,74E-05	MSRB1	-0,55046	4,136456	0,000151
ENTHD2	0,498931	5,496821	1,17E-05	STOX1	-0,58181	3,702176	0,00023
SLC2A11	0,438804	4,028583	0,000245	CMC2	-0,3247	5,476199	0,00059
KLHL28	0,47255	4,172805	0,0003	MEIS1	-0,83356	4,022397	0,000158
ZNF354B	0,449446	4,81231	3,81E-05	NSDHL	-0,41149	4,724334	7,13E-05
DNASE2	0,683377	5,672825	6,24E-08	MAD2L1BP	-0,28475	4,652542	0,00145
ROGDI	0,339115	4,871228	0,000139	KTN1-AS1	-0,45719	2,029772	0,003231
RAB30	0,659081	3,448985	1,6E-05	AGGF1	-0,50431	5,842635	8,64E-07
PPOX	0,204003	4,759943	0,035618	YRDC	-0,27087	4,34799	0,009612
ZNF599	0,481633	3,555316	0,004742	GTPBP4	-0,54881	7,085215	8E-08
RP11-458F8.4	0,643481	3,622886	0,000284	SEMA6B	-0,52804	5,04395	0,000104
ZNF324	0,576958	3,519699	0,000195	ORC5	-0,33715	4,579468	0,001356
WDR45	0,487033	4,421371	7,57E-05	MRPL27	-0,35618	5,160848	0,000165
SRGAP3	0,657676	3,176445	0,000232	TRIM65	-0,24032	5,836872	0,002063
PKD1	0,284063	7,402794	0,000258	NUP205	-0,39417	6,837436	3,26E-05
RP11-715J22.6	0,761238	1,10426	0,016188	ARL6IP6	-0,90408	5,102613	2,57E-07
ZNF717	0,846006	3,239689	5,06E-05	C2CD3	-0,95983	5,378681	2,36E-09
RP11-706O15.	0,555779	0,772912	0,049111	ENOPH1	-0,39634	5,523078	1,42E-05
IFT46	0,417117	3,896296	0,00171	HAUS1	-0,59429	6,021877	1,1E-08
HSPG2	1,040091	7,648652	1,23E-09	SMARCE1	-0,24489	6,244684	0,001156
CST3	0,545332	6,755227	2,56E-07	PSMD1	-0,40504	7,821842	0,000136
QSOX1	0,470211	7,427369	1,18E-06	CEP57	-0,69021	6,054403	4,65E-08
GAMT	0,152817	4,67926	0,023969	ZNF709	-0,57469	0,267185	0,031762
ITPKC	0,272134	3,99757	0,016104	EFTUD2	-0,17825	7,304109	0,029286
NPRL2	0,334037	4,569734	0,000641	C4orf27	-0,44135	4,881529	8,72E-05
CTD-2368P22.	0,343025	3,411602	0,026655	SMARCB1	-0,49382	5,932118	3,15E-07
ZNF767P	0,711102	4,38093	1,45E-06	PPP2R1B	-0,6559	5,621089	6,65E-08
RP11-6N17.4	0,401131	2,03617	0,03107	KRT10	-0,29029	4,318474	0,008565
LONP2	0,335796	6,967795	0,000121	MRPS7	-0,26852	6,291585	0,000917
RP11-352M15	0,779941	4,287107	7,43E-08	SMC3	-0,53537	7,431723	4,62E-08
RP11-334C17.	0,806609	4,16144	4,79E-05	RTN4IP1	-0,35103	4,020612	0,000479
EPHX2	0,391294	2,001746	0,001835	ZC3HC1	-0,80578	4,659144	2,07E-09
KDM7A	0,892796	4,387436	3,26E-06	SUV39H2	-0,30866	5,088408	0,001912
USF2	0,32665	6,296897	0,000375	C8orf76	-0,55555	2,13598	0,006402
RP11-159D12.	0,823839	4,968335	1,9E-08	RHNO1	-0,21731	4,750695	0,006679
THTPA	0,524553	2,504593	0,006289	STOML2	-0,27892	7,166351	0,000118
DDX60L	0,299908	4,656024	0,006792	MPHOSPH6	-0,43283	4,704416	5,91E-05
ZNF33B	0,755224	5,592435	1,03E-06	RDH11	-0,55795	6,380264	6,84E-07
ZNF529	0,305416	5,680518	0,008496	PGP	-0,2811	4,931727	0,016998
PPP1R12B	0,688518	4,943641	1,32E-06	SPAG1	-0,46569	4,735116	1,8E-05
NR1H3	0,580021	3,862263	4,55E-05	NFKBIB	-0,62327	4,143647	0,00011
PCMTD1	0,593598	5,620622	3,06E-05	PSMD14	-0,40642	7,441071	1,32E-05
LXN	0,447472	3,685302	0,010928	RP11-582E3.6	-0,63692	3,760663	4,48E-05
TNFRSF1A	0,401628	7,016693	1,18E-06	FRK	-0,89273	0,918692	0,032257
ICA1	0,459363	3,160922	0,016074	YARS2	-0,41588	5,457985	1,83E-05
SH3BP5	0,378103	4,681144	8,29E-05	NXT1	-0,43985	4,2705	0,000162
C2orf27A	0,45776	2,290104	0,012495	ATP11B	-0,89108	5,84106	3,86E-09
ZNF37BP	0,335896	6,930987	0,000527	TMEM60	-0,58783	3,531764	0,00073
IKZF5	0,56619	4,608391	3,37E-05	MRPS35	-0,12634	6,490422	0,038959
OPLAH	0,153857	4,034435	0,048937	RAD50	-0,56106	6,774252	4,09E-08

PRNP	0,51256	6,992765	3,29E-08	CCDC59	-0,2275	5,240717	0,023261
XIST	0,642023	9,720647	4,75E-05	RRP15	-0,24066	5,197302	0,003439
ZNF234	0,258478	4,34849	0,036216	PSMA3	-0,54888	6,592846	5,45E-06
TMEM80	0,484687	4,161522	2,47E-05	IWS1	-0,66039	6,570163	7,65E-10
DPCD	0,263868	3,872839	0,02524	NEBL	-0,56003	5,700597	2,03E-05
PEX11B	1,01041	5,335316	1,9E-10	MRPL33	-0,24981	5,970086	0,001843
SSPO	0,404659	1,70827	0,000912	ARF6	-0,51903	7,183164	1,94E-08
TMEM42	0,472102	3,710191	0,000773	YWHAZ	-0,51181	9,131386	2,18E-06
SLC25A45	0,507651	3,342908	3,78E-05	VBP1	-0,46143	6,231914	4,13E-06
NAGK	0,538257	5,506865	3E-07	CCDC85C	-0,24595	5,224548	0,014811
ZDBF2	0,431146	5,553072	0,00186	ZNF385A	-0,39859	4,888421	4,63E-05
HEG1	0,705497	5,779938	1,99E-07	FAM49B	-0,37371	6,696756	3,49E-06
SNX33	0,464805	5,376328	6,96E-06	CISD1	-0,75938	4,283401	2,24E-07
HIPK2	0,889995	5,354587	1,03E-06	ATG5	-0,39955	5,598556	1,97E-05
MNT	0,382634	5,601089	7,03E-05	ZBTB42	-0,91974	3,051417	2,2E-05
HERC2P2	0,454872	2,123971	0,00662	ARMC7	-0,43671	4,564372	0,000742
SHROOM1	0,627175	5,28425	1,36E-07	NDUFB9	-0,26661	6,531522	0,000585
EHD3	0,468682	3,714547	1,97E-06	GEMIN5	-0,94379	5,372575	2,41E-09
FAM193B	0,406236	5,800792	0,000371	ATP5G1	-0,23464	6,112384	0,041326
RAP2C	0,726149	6,287924	1,4E-09	CNP	-0,41244	6,283193	6,48E-06
ZNF550	0,556265	4,674729	1,99E-05	DOT1L	-0,19214	6,390596	0,022265
NADSYN1	0,186087	5,694908	0,034036	C14orf1	-0,37883	4,621065	0,001254
AHR	0,596732	4,050726	0,013167	PSMB2	-0,29308	7,405098	0,002017
FBXL2	0,342132	3,870336	0,018905	TXNRD1	-0,18233	7,571287	0,021436
SZT2	0,308056	6,38771	0,001856	SLIRP	-0,46875	4,978471	0,000206
FBXL17	0,528756	4,86273	0,000176	POP7	-0,44702	4,187139	0,000298
IGSF8	0,362441	5,400396	2,98E-05	FAM98B	-0,60945	5,489646	1,16E-06
PARP3	0,527117	4,993117	2E-06	KANK1	-0,34925	5,879936	2,7E-05
SSR4P1	0,671002	0,816999	0,016205	MRPL19	-0,35628	6,353631	8,86E-06
AKAP9	0,332558	7,549145	0,000297	MRPL13	-0,44061	5,104743	2,51E-05
GRAMD1A	0,357116	6,261259	1,97E-05	40422	-0,98219	7,054352	1,97E-11
DBNL	0,254152	6,684865	0,000871	BAG2	-0,40909	5,582329	4,03E-06
MOAP1	0,748777	5,328502	4,9E-06	WIBG	-0,51437	4,583736	5,89E-06
MCF2L	0,939118	3,456153	0,000141	PRMT3	-0,35429	5,368901	5,45E-05
SERINC3	0,65017	7,139885	2,35E-08	AIMP1	-0,55932	6,070623	9,61E-08
CHD6	0,238242	6,593834	0,006495	PLP2	-0,40457	6,296894	0,000656
MOV10	0,267872	8,265864	0,000343	CYC1	-0,41398	6,645445	6,03E-06
ANTXR1	0,183899	6,33394	0,026688	C14orf80	-0,29427	3,992063	0,039748
CFAP221	0,507888	2,609399	0,008877	XRCC5	-0,47256	9,049927	1,69E-06
PDCD4	0,254714	5,367639	0,012164	RAD54B	-0,45309	2,859033	0,001183
HIVEP2	0,654671	4,655204	0,00016	PAQR3	-0,48947	4,549994	2,28E-05
CD27-AS1	0,586304	3,145127	1,89E-05	40787	-0,54167	6,962258	2,3E-07
ARHGAP17	1,031427	6,627411	4,62E-12	MED21	-0,22194	5,091774	0,009891
ALDH5A1	0,895268	5,583116	5,51E-10	CKB	-0,62361	6,14318	1,12E-06
GHRLOS	0,565199	1,676848	0,009793	PSMC3	-0,3868	6,931159	0,00013
PPDPF	0,722328	6,382365	6,86E-06	ITGA6	-0,46165	6,095047	8,23E-06
CMTM8	0,304006	2,44244	0,007501	ZNF107	-0,39606	4,960066	4,89E-05
CCDC78	0,314135	3,120845	0,019208	TIMM13	-0,36095	5,440894	0,000114
CD59	0,306348	6,679777	0,000114	FLNA	-0,96468	10,04597	4,9E-11
H1FX-AS1	0,690833	2,073535	0,00394	RAPGEF3	-0,55374	4,38742	0,040387

SARAF	0,760198	7,258478	1,9E-10	ATP1B3	-0,42814	6,688772	4,95E-05
TBC1D8	0,597408	5,817609	2,31E-07	FGFR3	-0,73688	4,327607	1,08E-06
HLA-H	0,415656	2,188515	0,029035	XRCC6	-0,40136	8,537375	1,17E-05
ZNF155	0,242823	4,274868	0,023516	NABP2	-0,30626	5,403873	0,000746
VPS51	0,233101	5,68277	0,004659	UTP18	-0,44529	6,474192	2,68E-07
PDE8A	0,720538	6,008744	1,22E-09	IMPA1	-0,71221	5,349045	4,02E-08
MALT1	0,270464	6,436539	0,00414	MRPL42	-0,49303	6,649112	1,43E-07
GHDC	0,465606	4,197282	1,94E-05	SBK1	-0,20989	2,323888	0,049063
ACAD8	0,294568	5,224628	0,001269	RPS23P8	-0,55984	1,739101	0,002281
ZNF417	0,398775	4,091512	0,03337	FAM168B	-0,53663	8,025194	1,06E-07
SENP7	0,676563	5,145459	5,03E-06	PNO1	-0,16812	5,35593	0,032822
ABCC5	0,449562	6,297603	1,07E-05	ZNF664	-0,92321	7,588366	1,15E-11
LINC00839	0,528966	4,688525	2,14E-05	PTRH2	-0,31828	4,953409	0,001414
TMEM43	0,44127	6,09864	1,71E-06	THOC6	-0,62685	5,531843	1,13E-06
LIMD1	0,342866	5,978093	2,25E-05	NAA15	-0,22872	6,660609	0,00188
TRIM23	0,738561	4,48871	2,92E-06	TOMM40	-0,59681	6,489227	6,37E-07
SLC35G2	0,785567	4,150236	1,71E-08	TCF3	-0,3353	7,091798	0,00036
FAM66C	0,597868	1,165694	0,006909	MAPKAPK5-AS	-0,53352	3,956515	8,68E-05
KIAA0319L	0,445974	6,119319	4,16E-06	UTP20	-0,34298	5,942732	9,24E-05
ZNF780B	0,34705	5,672178	0,007722	ABCF1	-0,28565	6,710939	0,000567
PTPN4	0,889893	5,943953	1,49E-09	ATP5G3	-0,1609	7,577072	0,039602
SCAP	0,596261	6,630101	2,28E-09	LSM4	-0,49829	6,305048	5,92E-05
RPS6KA5	0,703965	3,61401	0,000733	ITGB3BP	-0,46659	5,116766	3,03E-05
SSBP4	0,443488	6,210572	2,01E-05	AGPS	-0,18551	7,414153	0,005538
PAN2	0,398854	5,727195	0,002023	SEPHS1	-0,45633	6,713245	2,62E-06
TK2	0,741666	5,326757	9,78E-08	SH3BP4	-0,67903	5,558696	7,95E-07
ZSWIM6	0,367152	4,939421	0,001583	TCP1	-0,34087	8,30641	0,000209
SERINC1	0,479005	6,948662	5,95E-07	RNPS1	-0,26041	7,268463	0,000155
KIZ	0,296791	4,418441	0,044558	CCT6A	-0,18696	8,198074	0,007372
WDR48	0,596587	5,766528	1,01E-06	RRP36	-0,2696	5,238673	0,004341
PPP1R3F	0,553252	1,73836	0,002323	ZFP82	-0,50383	4,71838	0,019174
RP11-295P9.3	0,518949	4,699883	0,003767	STAM	-0,8092	5,375952	7,75E-10
ZNF316	0,210399	5,345627	0,029465	IDH1	-0,51837	7,004531	5,07E-08
HARS2	0,426566	5,836834	7,03E-07	SMUG1	-0,65983	4,161986	4,76E-06
FRS3	0,452421	2,963923	0,012943	CBX1	-0,23544	7,801086	0,027975
POMGNT1	0,207618	6,596105	0,003034	NEK3	-0,48642	4,595646	1,82E-05
LRRC75A-AS1	0,691004	7,888989	1,33E-08	DOLPP1	-0,39919	3,864431	0,001237
KIAA1468	0,411459	5,567961	1,48E-05	SNAPC5	-0,56277	4,220268	1,1E-06
RP3-329A5.8	0,514796	2,05054	0,041054	NTHL1	-0,51587	4,217079	8,92E-06
WDR55	0,47995	6,108016	2,53E-07	FAM136A	-0,38824	6,80152	1,43E-05
RPL13A	0,204135	9,98272	0,003439	BAZ1A	-0,87068	6,258118	5,01E-11
SLC12A7	0,405969	5,646034	1,28E-05	GAR1	-0,17019	5,061501	0,040147
RP11-884K10.	0,760457	2,983648	2,78E-05	FOXK2	-0,30628	6,896931	3,73E-05
ZNF181	0,585658	3,848706	0,001606	NCBP1	-0,23381	6,443707	0,001492
UBL3	0,928225	5,424976	6,61E-10	HNRNPA1	-0,15084	9,606602	0,027182
C2orf81	0,666939	3,261933	8,21E-05	AC133528.2	-0,45917	1,909794	0,016209
MAN1A2	0,624453	7,360421	7,36E-09	NBL1	-0,87264	3,770183	0,000101
NR1H2	0,432716	6,115459	1,45E-06	CDK12	-0,21714	6,916256	0,012131
GLI4	0,352756	4,003306	0,006573	SNRPA1	-0,42632	5,480237	6,42E-06
NUMA1	0,336361	8,332584	5,34E-06	PECR	-0,61114	4,131193	4,15E-07

MAU2	0,257941	6,083683	0,001696	SLBP	-0,41339	6,210892	3,25E-05
ZNF227	0,525548	4,881164	3,07E-05	C7orf49	-0,19967	5,381502	0,010287
PNKP	0,359468	6,489641	6,25E-05	KTN1	-0,59683	8,530819	2,67E-09
RP11-1277A3.	0,657712	1,836965	0,006814	PCYT2	-0,27269	5,838534	0,001273
ETV5	0,350549	6,297195	0,001175	NIPA2	-0,32635	6,253938	0,000201
NCOA1	0,655786	5,749872	1,15E-07	MED18	-0,41428	4,442406	0,001071
ERGIC1	0,146724	7,174609	0,022212	PSMD3	-0,22615	7,593664	0,004111
FBXO25	0,694855	4,587001	4,1E-05	GLO1	-0,31171	7,832213	0,000555
THNSL1	0,648739	4,572855	1,32E-05	MRPL11	-0,27418	5,313036	0,009905
VRK3	0,312603	4,954801	0,000636	MRTO4	-0,27814	5,921431	0,000819
STX4	0,437248	5,980243	2,23E-06	TMEM120B	-0,38887	5,906939	0,000426
MARVELD1	0,581242	5,763413	3,34E-07	GTF2A2	-0,26772	5,704996	0,000878
ALPK1	0,852314	3,848914	1,4E-05	CCNJ	-0,22452	4,241923	0,006318
IL6ST	0,878947	7,661224	2,14E-09	HNRNPD	-0,36541	8,845129	0,000151
TOM1	0,445802	4,580113	0,000227	BUD13	-0,43299	4,692304	2,52E-05
TBC1D32	0,361758	4,792831	0,001445	HDGFRP3	-0,42949	6,354272	1,97E-06
PAM16	0,682079	3,082281	0,001016	BIRC3	-0,70458	2,313686	0,000395
GSE1	0,434994	6,599903	1,9E-05	KIF5B	-0,55262	7,933449	5,21E-08
ZNF304	0,26892	4,627787	0,017323	PIK3C2A	-0,57619	6,835574	6,28E-07
BRD8	0,734101	6,851635	4E-09	ILF3	-0,22034	9,00088	0,003334
DNAJC28	0,679879	1,701799	0,009706	TDP1	-0,30342	4,357337	0,001899
RUNX1	0,431201	4,46932	0,03538	TAF5L	-0,20354	5,468434	0,007622
CHPF	0,464016	6,089105	2,47E-06	SSRP1	-0,27747	8,174032	0,000192
TEP1	0,345599	5,551644	0,000845	CHRAC1	-0,48861	5,297222	1,71E-05
EXD2	0,646813	5,435088	3,43E-07	KIAA0319	-0,68142	1,447419	0,047882
ANKRD45	0,318842	2,432623	0,000583	MTA2	-0,40729	7,525546	1,07E-06
KRT18	0,796083	7,060955	1,02E-09	TTC27	-0,55671	5,395949	5,14E-08
HLCS	0,818845	5,646431	3,21E-09	RPS12	-0,77206	8,576508	7,43E-10
CFAP44	0,719751	4,621078	0,000278	ADH5	-0,58383	7,448468	2,63E-09
PAN3	0,556494	6,138707	1,21E-06	ACTR3	-0,20592	8,262133	0,000805
JMJD1C	0,602399	7,086558	6,53E-07	RPS6KL1	-0,26115	3,496301	0,011396
C19orf57	0,780178	2,983661	1,15E-05	TCF7	-0,61431	3,231366	2,61E-05
RABEP2	0,404455	5,063671	9,91E-06	VMA21	-0,51403	6,18616	3,57E-06
EDEM3	0,686824	5,797562	4,55E-09	PAK1IP1	-0,17495	5,073327	0,014551
USP20	0,463743	5,004307	8,77E-05	RWDD4	-0,46964	3,56455	0,000253
SCNN1D	0,546689	3,653823	0,000257	DNM3	-0,72368	5,51821	4,23E-10
DGKQ	0,293761	5,357629	0,002461	MAGOH	-0,19459	5,520046	0,031844
PRKAB2	0,491025	5,916363	2,6E-06	EEF2KMT	-0,36377	3,548286	0,001042
SEC24C	0,369123	7,80408	2,69E-05	OMA1	-0,78057	3,883626	5,39E-06
RNF32	0,453791	2,315553	0,001434	RAB3D	-0,51256	4,974291	8,36E-06
LRTOMT	0,596616	2,739942	0,001575	ZFAND1	-0,48507	5,69679	9,6E-05
ICK	0,378034	4,750221	0,000256	WDR4	-0,29473	4,572709	0,002393
GLG1	0,317156	7,928562	2,97E-05	PUS1	-0,3523	5,212524	0,000167
KLHL7-AS1	0,472718	1,650617	0,040103	RHOT1	-0,27541	5,591362	0,000772
ZCCHC11	0,212702	6,986414	0,009878	FOSL2	-0,72741	6,126243	4,03E-07
CCDC120	0,313763	3,928602	0,000412	ANKRD13B	-0,16697	4,413868	0,031378
KLHL20	0,486936	4,88211	3,92E-06	ARID5A	-0,47109	2,976036	0,00118
AC024560.3	0,560278	4,595085	0,000109	TIMM10B	-0,23737	5,107161	0,003101
SEMA3B	0,621393	4,879575	8,63E-05	RGS19	-0,37944	3,645528	0,003845
FAM154B	0,421122	1,372488	0,027523	USP39	-0,18176	6,549469	0,017306

MGEA5	0,333815	7,773277	0,000443	NASP	-0,18672	8,137583	0,012938
ANKRD44	0,429226	4,120284	3,57E-05	MRPS10	-0,1343	5,734142	0,049442
TXNDC15	0,403422	5,791636	4,08E-06	CDC34	-0,2525	5,419813	0,002726
GAA	0,168122	6,92243	0,032892	NTMT1	-0,30437	5,253923	0,002318
DOPEY1	0,811096	4,635937	1,11E-05	CCT4	-0,19831	8,334621	0,002248
RAB24	0,704628	2,39464	0,001159	PDZD4	-0,64627	4,164552	8,93E-08
PAIP2B	0,554434	1,892962	0,003215	FAM118B	-0,56072	4,056908	1,45E-05
ZNF532	0,255731	4,982559	0,003094	NOL11	-0,26024	6,76447	0,000226
CYB561D1	0,711239	5,744615	2,99E-09	QRSL1	-0,23912	5,215269	0,004867
KDM2A	0,260183	7,208027	0,001179	NUDT9	-0,56002	4,859936	8,55E-06
FGGY	0,284748	3,339231	0,038633	CDK5RAP2	-0,17255	6,75272	0,021436
39508	0,674266	5,524508	4,19E-07	SNRPD1	-0,29326	6,604635	0,00169
RSBN1	0,633031	5,903092	1,16E-08	MATK	-0,81037	4,836058	3,53E-09
SWT1	0,38367	2,82615	0,042233	COMMD8	-0,51365	4,760398	3,02E-06
EVC	0,337124	5,536153	0,001807	GLMN	-0,6342	3,954745	4,9E-07
DDX60	0,788223	3,967642	1,66E-06	CTDSPL2	-0,37902	6,13705	4,07E-05
CTD-2574D22	0,454011	2,563012	0,013524	SLC25A15	-0,33969	4,313122	0,000812
C3orf67	0,416953	2,020116	0,009286	NUP54	-0,60794	6,296044	9,28E-09
IFT172	0,200895	5,799104	0,035431	TMA16	-0,24613	4,820444	0,002634
NARFL	0,687469	5,046037	2,49E-07	C11orf84	-0,55221	5,416733	3,36E-07
FDXR	0,542205	4,894894	1,24E-07	SMARCD1	-0,3272	6,625249	2,17E-05
NAB1	0,775839	6,284477	3,67E-09	PREX1	-0,70201	4,834481	3,23E-06
ACOT8	0,408594	3,698044	0,001902	NUP133	-0,32067	6,166048	5,99E-05
RP11-481C4.2	0,408831	2,461317	0,032001	KCMF1	-0,26384	6,656957	0,000849
UBBP4	0,315224	5,182549	0,001525	LSM5	-0,17898	5,417449	0,014109
MAP3K5	0,423291	3,944695	0,002799	PROSER1	-0,40144	6,232898	2,38E-05
NBEAL2	0,227064	5,98424	0,005229	DCUN1D5	-0,3412	6,00803	0,000388
IQCH	0,525199	2,35334	0,008242	PTGES3	-0,36938	8,697244	4,48E-05
AHCYL2	0,405135	4,800081	0,000121	PTGR1	-0,4324	6,196339	5,46E-06
NNT	0,400256	5,917191	3,31E-06	HAUS3	-0,60836	4,707904	6,91E-07
CIC	0,603601	7,140385	1,15E-07	IPMK	-0,78379	4,320438	2,04E-07
LRP10	0,566569	6,883041	8,43E-09	ITGA5	-0,81651	5,461247	4,59E-10
TMEM254	0,339166	4,604598	0,004992	TTC7B	-0,70609	4,283831	6,16E-06
GNAI2	0,300893	7,561938	0,000748	NDUFA6	-0,36456	5,756157	9,11E-05
IDNK	0,365968	3,475135	0,030611	ING1	-0,27272	4,202971	0,022657
TTC14	0,260093	6,193104	0,009445	KRAS	-0,17398	6,446916	0,011895
XRN1	0,761952	6,184937	1,44E-07	SRPK1	-0,42466	7,222743	8,39E-07
LPCAT1	0,546903	7,39903	1,65E-05	VPS33A	-0,22572	3,980438	0,020142
ZNF879	0,621517	3,48005	0,000334	TBPL1	-0,67554	4,174906	2,62E-06
NAB2	0,382393	6,206301	1,73E-05	RRAS2	-0,34386	5,437843	0,00046
GOLGB1	0,571889	7,772741	3,17E-07	MSH6	-0,28668	7,395159	0,000298
PRKCZ	0,523545	5,060213	1,96E-06	TAF1A	-0,31039	3,958829	0,001049
KCNC3	0,634778	5,868771	3,15E-07	DUS4L	-0,49642	3,120326	0,001184
ASH1L	0,354389	7,260743	0,000478	ATP5J2	-0,68783	5,403163	1,94E-05
RBM6	0,126976	6,858132	0,037738	TCEB1	-0,30861	5,976619	0,000494
ENDOV	0,277076	4,671834	0,003568	CCDC107	-0,40764	3,561298	0,006551
GOLGA1	0,518217	5,632757	1,8E-06	SRP9	-0,20417	7,730483	0,002062
TEX9	0,38404	3,221314	0,047536	RRP1B	-0,29817	7,029638	8,56E-05
DFNB31	0,628469	4,694718	2,14E-05	DENND5B	-0,3193	5,832334	0,000468
AC096772.6	0,524308	1,684021	0,021864	MSH5	-0,3821	3,347248	0,007302

CCDC24	0,335331	4,533754	0,010641	EXOC6	-0,49886	3,805398	0,000313
OS9	0,306796	7,253452	2,13E-05	EIF3G	-0,31339	6,900484	0,000121
ZNF783	0,409307	4,204959	0,001111	CTD-2562J17.6	-0,43551	3,336537	0,001963
RHOC	0,544577	7,949084	5,34E-07	ARRB1	-0,33199	3,891566	0,011074
RNF123	0,350178	5,570862	3,33E-05	ZNRD1	-0,24599	3,749547	0,015617
EXOSC5	0,185705	4,218012	0,042102	PRKAR2B	-0,75416	4,236917	7,09E-07
RP11-337C18.	0,329557	2,167782	0,006793	NME1-NME2	-0,40992	1,878294	0,014679
OBSCN	0,384951	7,070128	0,000214	ARL3	-0,50011	5,410545	4,08E-06
TPT1	0,433537	10,52903	1,34E-06	PTMA	-0,34459	9,735658	0,002225
SPHK2	0,528271	4,616874	0,000108	UBL5	-0,33591	6,386442	0,000185
SUCLG2	0,309026	6,204877	0,00011	NUPL1	-0,18538	6,946949	0,003404
KAT6B	0,33834	5,572212	0,002105	RHPN2	-0,32287	4,161582	0,003441
ERCC6	0,491532	3,757583	0,00063	MCM7	-0,19933	7,455591	0,035397
HMGCL	0,516067	4,719244	0,000898	SRSF7	-0,23081	7,821095	0,001865
GAS6	0,430943	4,875783	0,004111	CPEB1	-0,57786	2,59764	0,000227
APP	0,145446	9,263287	0,011439	DNA2	-0,38782	5,732083	0,000247
UACA	0,169364	6,583251	0,031947	DHX15	-0,14801	7,822084	0,025085
PARP9	0,48297	5,043881	5,53E-05	ZNF101	-0,40801	4,211042	5,48E-05
GLB1	0,375715	5,828524	4,63E-05	CSE1L	-0,21291	8,24411	0,006956
DBP	0,193078	4,212626	0,044439	FXN	-0,2455	4,249302	0,002786
CRAT	0,462435	6,189333	1,21E-06	HMBS	-0,63423	5,071564	5,07E-07
ZADH2	0,512697	6,140015	7,87E-08	ABHD17C	-0,19413	4,065452	0,025085
IL17RA	0,452737	5,353946	3,63E-06	MYRF	-0,44595	4,959973	0,000236
SPATA2L	0,4627	3,948188	0,000295	HNRNPR	-0,22434	8,073191	0,005971
ATG2A	0,619898	5,383012	1,61E-07	FARP1	-0,56086	6,166659	5,88E-07
WDPCP	0,33808	3,106879	0,041555	MRPS5	-0,57516	6,60701	2,89E-08
CTD-2574D22.	0,295125	2,44424	0,038013	NT5DC3	-0,25625	4,891267	0,012407
BMP2K	0,786559	5,487975	6,57E-07	MNAT1	-0,26252	4,545473	0,002005
ACVR2A	0,496174	4,323625	3,7E-05	RPF1	-0,51135	5,138365	2,07E-06
TNK2	0,259936	6,343213	0,002459	TTPAL	-0,34657	5,450728	0,002139
NR2C2	0,279632	6,049402	0,001244	DAXX	-0,19683	6,161297	0,003055
FYCO1	0,410434	5,521756	6,64E-05	MTMR9	-0,706	5,351361	2,63E-08
SYVN1	0,533423	6,120795	1,5E-07	PSMD12	-0,23091	7,051279	0,003213
NUDT17	0,582845	1,940769	0,01226	TMX2	-0,21159	5,711547	0,004957
C1orf216	0,382485	5,00814	4,66E-05	WDR45B	-0,40011	7,241656	3,57E-06
GDPD5	0,685175	3,654375	5,05E-05	LIF	-0,44135	5,154165	0,003901
IK	0,349835	7,009428	9,58E-06	ACLY	-0,35528	8,398665	0,000187
ERO1LB	0,649489	3,955563	2,81E-06	POLR2D	-0,19912	6,242003	0,00635
RPL15	0,232953	8,679066	0,000932	SNX8	-0,57109	4,746032	7,14E-06
MPPE1	0,198645	4,670345	0,014667	CALM2	-0,39198	9,072024	7,09E-06
PURA	0,419497	5,879771	6,76E-05	ADRBK2	-0,44451	3,331236	5,76E-05
ALDH3B1	0,331653	5,277185	0,004643	NDE1	-0,27984	5,043728	0,000866
AP2A1	0,22763	7,333682	0,001791	HS6ST1	-0,4546	4,547393	2,11E-05
ARID4A	0,379463	5,363412	5,47E-05	HSPA14	-0,38854	5,875888	7,92E-06
EXT2	0,198102	6,125671	0,004557	STK26	-0,21146	5,607857	0,006545
NCOA3	0,507442	6,416928	4,42E-06	OTUD6B-AS1	-0,4655	4,635196	7,58E-05
KMT2C	0,378637	6,308221	0,000185	AAGAB	-0,25933	6,304297	0,000316
RFNG	0,385305	5,652769	0,000168	ZCCHC9	-0,31198	5,197377	5,05E-05
USP19	0,391361	6,196923	1,02E-06	UBAP2	-0,19031	6,228146	0,016701
TRMT13	0,241533	5,128371	0,004797	CEACAM19	-0,40784	4,466297	0,022575

MICU1	0,711304	6,505523	3,22E-09	PSMG1	-0,2273	6,168399	0,003431
CLPTM1	0,403004	6,914652	2,61E-05	DUT	-0,38006	6,314546	7,08E-05
LINC01578	0,223772	5,434419	0,016086	DARS2	-0,30117	5,473507	0,000523
KIAA0226	0,536191	5,526731	1,46E-06	POT1	-0,50192	3,864414	0,000897
TAF1C	0,250267	6,427295	0,002435	LSM2	-0,41007	5,266469	0,000228
NTPCR	0,309024	5,677597	0,000201	SNRPC	-0,17679	6,091254	0,028643
ZNHIT1	0,164212	5,62295	0,03325	ATIC	-0,24019	7,102502	0,000512
PTPN23	0,217112	6,364	0,00321	NFYB	-0,23559	5,51354	0,004444
MGAT4B	0,399094	6,904395	1,97E-05	PSMC5	-0,19541	7,442429	0,011099
RASA1	0,458015	5,840752	1,07E-06	TOMM22	-0,21984	6,236702	0,016849
IFNAR1	0,475	6,291868	1,31E-06	CEBPZ	-0,47412	6,737589	9,51E-08
CDK5RAP3	0,249284	7,291889	0,001625	ZCCHC17	-0,45633	5,525898	8,52E-05
SYNJ1	0,348703	4,369147	0,008139	ABT1	-0,33035	5,241669	0,000163
ARAP1	0,265848	6,427327	0,000316	WDYHV1	-0,41414	3,99578	0,000491
D2HGDH	0,722104	5,461199	2,35E-05	TAF2	-0,21119	6,123516	0,002519
NGLY1	0,349444	5,595754	1,82E-05	IFITM2	-0,37323	6,381037	2,33E-05
C16orf93	0,296853	3,374819	0,018961	CFL2	-0,50309	6,431309	9,38E-07
EPB41L4A-AS1	0,686121	4,507105	3,99E-06	METTL2B	-0,52848	4,575134	1,27E-06
ZNF589	0,200023	4,68774	0,022924	FOPNL	-0,22137	5,883614	0,002569
RNMT	0,478564	6,767057	1,15E-05	ASUN	-0,19127	6,080532	0,005789
CDIPT	0,194839	5,683407	0,010259	HAGLR	-0,47722	3,751361	0,000326
ZBED9	0,372757	2,996924	0,012279	PNPT1	-0,24266	6,185657	0,000425
RPL28	0,277095	8,71232	0,000303	MTF2	-0,61987	5,600097	1,43E-08
TNRC6B	0,254947	6,551132	0,007354	SCFD2	-0,49198	3,210928	0,001626
TUBGCP2	0,577223	6,557517	1,78E-08	SH3RF1	-0,29851	4,060491	0,013841
CAPN7	0,371255	5,882515	4,15E-05	KLHL12	-0,26824	5,73566	0,000404
ATP9B	0,504216	4,909361	1,73E-05	SLC25A3	-0,28676	8,729787	0,000111
RNF44	0,383484	6,042126	9,25E-05	ADNP2	-0,59553	6,459566	2,49E-08
PRR7	0,331395	3,199794	0,034869	DDX46	-0,15509	7,440838	0,044484
TRIM21	0,238853	3,918704	0,022187	HDHD1	-0,53041	4,736318	5,63E-05
RP11-567M16	0,473386	2,371643	0,021597	CWC22	-0,66835	5,467215	1,03E-08
CCNG2	0,652443	5,033848	0,000109	BCL7A	-0,28514	4,767359	0,002634
MROH1	0,370763	5,833343	0,000513	HAUS2	-0,30156	5,137811	0,001295
RP11-574K11.	0,28519	4,546386	0,040446	STIP1	-0,2878	7,945037	0,005181
LRP3	0,381287	5,230552	0,000809	PSMA4	-0,42537	7,491077	1,38E-05
RXRB	0,195575	5,990418	0,033492	TNPO3	-0,42886	6,573945	1,49E-06
KDSR	0,386948	6,163023	3,72E-05	CCDC137	-0,24888	6,13449	0,00226
NOMO2	0,516701	3,090155	0,00041	EIF5A2	-0,48068	5,149671	1,26E-06
CFLAR	0,374382	5,961766	0,001595	PALM	-0,67741	5,526586	1,2E-06
HEATR5B	0,50387	5,870266	4,59E-06	LYSMD3	-0,68934	4,574442	1,67E-06
CRBN	0,466239	5,2552	0,000197	NIF3L1	-0,19219	5,164006	0,018402
PIGT	0,216721	6,390255	0,002036	TMEM14B	-0,20523	5,944799	0,017283
ETFB	0,423786	4,730071	0,000277	C22orf29	-0,55017	5,014362	1,26E-06
MTHFSD	0,309006	3,992866	0,014762	SNRPD3	-0,18804	6,244907	0,034284
SMPD1	0,26675	3,837241	0,019519	TYW3	-0,33383	5,231399	6,88E-05
S100A13	0,308813	5,926421	0,000882	ATXN10	-0,18051	6,866294	0,004474
KRBA1	0,442347	4,491746	0,000114	HDGF	-0,36135	9,225308	4,04E-05
MRPS25	0,562787	6,289062	9,05E-08	DLAT	-0,40354	6,408189	1,46E-05
C6orf48	0,31104	5,612943	0,005638	GBAS	-0,72381	5,856266	1,4E-09
RNF145	0,532026	7,063589	1,46E-07	EMG1	-0,19496	4,167813	0,013013

FNTB	0,598224	2,921584	0,002756	TMEM223	-0,27103	4,096117	0,009452
RP11-1023L17	0,466288	2,049496	0,027027	SMNDC1	-0,3877	5,976803	8,19E-06
ZNF324B	0,507276	3,316937	0,00059	FUNDC1	-0,44985	4,532192	1,91E-05
MAN1B1	0,239764	6,565173	0,000931	SLC29A2	-0,5323	5,326997	6,78E-07
MBD5	0,218354	5,062007	0,011937	ALS2	-0,69779	5,877827	6,34E-09
PIK3C3	0,249678	5,656339	0,01196	CSTF2	-0,40781	4,947765	8,83E-05
TUBGCP6	0,451073	6,158595	0,000106	ORMDL2	-0,41717	4,312872	0,000855
CPEB4	0,391114	4,135057	0,005286	MRPS18C	-0,29043	4,787523	0,000421
RPL14	0,440983	8,528388	1,93E-05	CCNE1	-0,40758	4,388301	8,09E-05
FBXO4	0,591335	3,925466	2,06E-05	UTP3	-0,28239	5,401119	0,00098
TTC12	0,437305	4,608954	0,000303	TFDP1	-0,36868	6,86547	0,000627
C16orf58	0,285143	6,343691	0,000869	BAIAP2L1	-0,69184	5,446064	1,23E-09
WDR44	0,305113	4,351625	0,002489	AGBL5	-0,40421	5,962731	1,36E-05
DMTF1	0,485799	7,095658	1,9E-05	TRAF2	-0,65618	4,672689	7,62E-07
SHISA4	0,466976	3,75795	0,000298	ACTB	-0,37141	10,6836	0,002668
FAM213B	0,611847	5,482385	4,18E-06	XPO5	-0,32914	6,884917	4,7E-05
USP30	0,413512	4,145587	0,000681	OPA3	-0,66661	4,861682	2,15E-07
KCTD13	0,365996	4,951116	0,000121	MTERF3	-0,30182	4,754105	0,000515
HADHB	0,586706	7,485322	4,05E-09	EEF1E1	-0,46582	4,436807	0,000105
AP001372.2	0,4311	2,234518	0,023111	CCNG1	-0,14529	6,908061	0,030148
MANBA	0,17357	5,01943	0,048342	CCHCR1	-0,46859	5,807605	3,7E-06
CEP350	0,433725	6,567895	5,16E-05	PDCL3	-0,20203	4,759305	0,027165
ZMAT3	0,237916	5,120106	0,00438	MTSS1L	-0,4468	6,122738	4,16E-05
ERAP1	0,403941	5,044567	3,38E-05	PPIG	-0,47325	7,406322	9,84E-08
DAK	0,458764	5,63585	6,53E-07	CCT7	-0,16841	8,760268	0,021106
CREG1	0,446584	6,76759	5,64E-07	DDX18	-0,30176	7,238524	4,37E-05
DNAJC4	0,450854	3,811779	0,002068	GPATCH8	-0,17303	6,573065	0,04334
VAMP4	0,495152	4,800635	2,76E-05	PPID	-0,37604	5,791353	5,27E-06
MON2	0,194578	6,453763	0,02226	PPA2	-0,35727	6,619746	2,57E-05
CAST	0,426491	7,254598	4E-06	MRPS6	-0,33078	5,621398	0,00282
RBL2	0,331523	6,857838	0,000118	ASPHD2	-0,4833	3,393455	0,000948
DTX4	0,170984	4,403286	0,044136	RPS6KA3	-0,58136	6,267718	4,07E-07
TSC1	0,172911	6,323926	0,032736	PUS7	-0,58179	5,607224	8,14E-09
ITFG1	0,470492	5,564618	8,22E-06	APOA1BP	-0,44229	6,595782	1,71E-06
TERF2IP	0,570952	5,743449	2,35E-06	ZBED4	-0,38824	5,467177	1,62E-05
KIFAP3	0,132454	5,340401	0,041608	CARD10	-0,31596	5,016874	0,000829
CREBL2	0,367364	5,138782	0,000179	CAMSAP2	-0,2265	6,641156	0,003247
CBX8	0,344377	4,84901	0,000448	PKN2	-0,68604	6,474928	1,53E-08
FAM53C	0,42426	6,445587	6,63E-06	PTPN11	-0,48806	7,726001	4,13E-06
SLC25A36	0,340678	7,506859	0,000751	MTFMT	-0,49597	4,554741	2,88E-05
RPL26	0,204784	6,977649	0,011242	GNA13	-0,1894	6,516982	0,004193
RBCK1	0,560741	7,020157	0,00021	NDUFA12	-0,26637	5,624425	0,004309
NPHP4	0,205669	5,293647	0,03221	NPC1	-0,25415	6,26994	0,001448
KLF6	0,286178	6,521328	0,000807	CCDC58	-0,23674	4,479509	0,005574
TRAK1	0,351745	6,380895	1,12E-05	CEBPZOS	-0,45841	6,604777	2,27E-07
HIPK3	0,565083	6,592251	6,45E-07	PSMB7	-0,23063	7,217558	0,010924
EIF3J-AS1	0,476567	3,158516	0,003929	SRRM3	-0,44094	2,973053	0,000705
RECQL5	0,677263	5,478074	4,9E-07	PIM2	-0,40138	3,616508	0,008995
INAFM1	0,479424	3,194994	0,001763	MED27	-0,35047	4,083336	0,000538
KDM5B	0,582211	6,714306	1,87E-07	HSPE1	-0,3129	6,184923	0,005582

PINK1-AS	0,510121	4,246688	3,15E-05	VTA1	-0,46709	5,826674	6,24E-06
C19orf54	0,330695	5,133263	0,002167	MRPL52	-0,30662	4,780617	0,000985
MLYCD	0,563721	3,56049	0,000139	GNPDA2	-0,34131	3,982358	0,001342
UVSSA	0,346078	5,006552	0,020349	PARP2	-0,36994	5,559796	2,7E-05
ZNF581	0,229886	4,590554	0,003428	BANF1	-0,15148	6,53572	0,043494
SEL1L	0,600681	5,912997	2,99E-06	SLC19A2	-0,36811	4,522978	1,11E-05
STRIP1	0,240627	6,4258	0,000404	NUP85	-0,36006	6,517596	7,65E-05
IQSEC1	0,221427	6,274673	0,004563	HN1L	-0,60713	7,378037	1,62E-07
LMLN	0,49784	4,547865	0,001962	COPG2	-0,55929	5,760689	1,57E-07
TCF25	0,207313	6,941193	0,002684	LPAR3	-0,62238	3,464108	2,41E-06
EPDR1	0,539636	4,875149	6,78E-05	APIP	-0,40804	4,850445	9,66E-05
TMEM106B	0,592861	6,269873	9,17E-07	VDAC3	-0,26933	7,102208	0,006112
RPL14P1	0,409015	2,429645	0,003552	UQCC2	-0,33896	5,205683	0,000222
ARL15	0,550199	4,52525	6,18E-06	ATP5L	-0,26492	7,118912	0,002558
EMC10	0,329501	6,209658	9,98E-05	RAB23	-0,34016	5,354573	0,000136
ARL6IP5	0,282237	6,013525	0,00053	RNF216P1	-0,29668	4,037787	0,003362
COMMD3	0,459331	3,99523	0,000591	MCPH1	-0,38411	4,929397	0,000705
LIX1L	0,374849	6,852822	5,04E-05	BID	-0,60786	4,210953	2,66E-06
PIAS2	0,157464	6,158828	0,022633	RNASEH1	-0,18261	4,727128	0,028661
ACAT1	0,445861	6,69473	6,6E-07	ADSL	-0,45549	5,979831	1,98E-07
CTDSPL	0,435059	6,00333	9,89E-07	INAFM2	-0,47955	2,656678	0,022203
LRIG2	0,303043	6,197185	0,000372	WEE1	-0,2078	5,88824	0,006551
ZCWPW1	0,489929	2,858293	0,00142	GTPBP2	-0,4342	6,413668	0,000629
QARS	0,249606	7,27089	0,000838	C1GALT1	-0,59111	4,608086	3,78E-07
PRR12	0,194733	6,385256	0,024426	PLEKHA8	-0,42444	5,2093	6,35E-05
IQCK	0,45879	3,902998	0,000858	CASP8AP2	-0,292	5,726415	0,000681
FBXW5	0,505668	6,603897	3,81E-06	EIF4A3	-0,1493	7,068884	0,037495
TP53TG1	0,299521	3,013656	0,002757	CDC123	-0,54553	6,862493	1,44E-06
PFDN5	0,40203	7,442298	1,13E-05	API5	-0,22681	7,370063	0,002064
TRIM41	0,467893	6,536669	1,21E-06	COX7B	-0,39023	6,619898	0,000134
STT3B	0,569709	7,740662	1,03E-08	MPP5	-0,23658	6,254121	0,002062
WDR47	0,300254	5,739409	0,000225	RAE1	-0,18258	5,875296	0,013609
STK19	0,394729	3,744698	0,004798	MCRS1	-0,27649	5,831839	0,00341
RPS27	0,203474	8,11622	0,008639	CIAPIN1	-0,31748	5,721907	9,27E-05
ABCF3	0,556032	5,985728	6,66E-08	COIL	-0,39386	5,419266	9,66E-06
PPP3CB-AS1	0,55872	2,817211	0,00818	ANAPC11	-0,40413	6,243062	1,9E-05
APEH	0,457591	7,135117	1,77E-06	ARL5B	-0,665	6,540896	8,19E-08
RBSN	0,414841	5,759696	0,000618	GNPAT	-0,46955	6,120514	1,17E-06
BTRC	0,221124	4,940375	0,002769	LARP1B	-0,68845	5,203397	4,86E-08
RBM4	0,34593	3,066952	0,004551	RPP38	-0,404	4,327405	0,001056
SPIDR	0,187659	6,038261	0,013124	C3orf33	-0,50308	3,15304	0,000689
RBM43	0,605047	4,881374	1,64E-06	PRKAG2	-0,63274	4,686275	1,04E-05
GOLPH3L	0,568534	5,461291	2,35E-08	CDC27	-0,19297	7,564128	0,004341
NCOA2	0,417654	6,120058	0,000131	BRMS1L	-0,44461	4,217787	4,43E-05
ELOVL4	0,575471	4,483714	1,18E-07	USP13	-0,17914	5,460804	0,019433
TCEANC2	0,424186	4,523346	0,000146	RPS6KA4	-0,45507	5,621657	0,000679
TBC1D9B	0,351916	7,774463	9,15E-06	DOLK	-0,32727	4,423261	0,003116
CEP170B	0,585223	6,29261	1,24E-06	FAM96A	-0,25572	5,446275	0,006538
SLC25A16	0,392026	5,309246	8,68E-05	C12orf49	-0,39323	5,755361	0,000273
RPL13P12	0,334752	5,026436	0,004932	CCDC86	-0,2531	5,146026	0,004754

TRPC4AP	0,238856	7,074063	0,000346	UBQLN1	-0,23207	8,050279	0,000515
CCDC50	0,681461	6,72877	7,3E-09	CYP2S1	-0,31051	1,735064	0,023582
FNDC3A	0,301776	7,022711	0,000319	CCDC23	-0,62649	3,732432	2,52E-05
ZNF18	0,320172	3,149328	0,011919	RNF4	-0,22839	6,619961	0,000976
KLC1	0,264088	5,121	0,013559	MTCH2	-0,37513	6,84934	0,000242
IP6K2	0,232753	6,077398	0,001377	GMEB1	-0,53929	5,14569	2,15E-07
ERVK3-1	0,392712	4,55772	0,002085	RAI14	-0,66386	6,784505	4,18E-08
GATAD2B	0,495746	7,076645	5,86E-07	MRPS15	-0,40722	6,611351	1,2E-05
CRELD2	0,505799	4,786007	6,27E-06	DAZAP2	-0,50795	7,434291	2,81E-06
TWF2	0,379605	5,013704	9,57E-05	MORC2	-0,34524	5,953603	5,94E-05
REEP3	0,473707	7,914447	6,05E-06	RCBTB1	-0,25374	5,692014	0,000483
BMPR2	0,355842	7,153635	3,09E-05	SCAMP5	-0,24677	3,549346	0,002702
OSMR	0,276889	5,826477	0,007278	PFDN6	-0,20802	5,45486	0,038952
CHD2	0,185021	7,13756	0,012397	ROBO1	-0,23825	4,981408	0,003044
METTL6	0,317158	4,438736	0,000841	UBE2N	-0,31912	6,693254	0,000519
FAM179B	0,584005	4,830154	1,81E-05	CRKL	-0,43526	6,644412	3,51E-06
ANKRD46	0,540996	4,105604	1,97E-05	PREB	-0,33904	6,427374	0,000136
BOD1L1	0,433466	6,60047	7,96E-06	PRIMPOL	-0,22172	4,623457	0,008249
OGG1	0,587606	5,026278	2,52E-08	MCTS1	-0,2805	4,924041	0,002109
RP11-504P24.	0,479856	2,738811	0,006439	TMEM206	-0,44144	4,390995	2,3E-05
ERF	0,514699	6,822484	1,15E-06	XPO1	-0,33858	9,133906	3,58E-05
NFKBID	0,401439	3,232571	0,000509	TAP2	-0,57158	4,612491	1,34E-06
BAIAP2-AS1	0,469661	5,887414	4,72E-06	WWC3	-0,30925	5,463541	0,002832
CTNNBIP1	0,209863	5,300236	0,006466	SART3	-0,27038	6,694129	7,88E-05
CES2	0,337091	6,525334	1,27E-05	BAG4	-0,17813	5,145173	0,045743
ITM2B	0,25448	7,968766	0,000245	POMP	-0,22381	6,773829	0,01161
TSPO	0,218321	5,806247	0,022299	C17orf58	-0,34312	4,673522	0,000213
GANC	0,343274	4,036372	0,002084	SCOC	-0,40764	6,01816	2,39E-06
MED12	0,293126	5,904574	0,003407	VWA9	-0,16011	5,693783	0,020797
JMJD8	0,553322	3,99694	0,02091	PSMD7	-0,39187	6,815906	3,57E-05
FMNL2	0,196861	7,143906	0,007097	MAPK6	-0,38441	6,293567	4,39E-06
TMEM63B	0,37744	5,826632	9,86E-05	PIP5K1A	-0,54303	7,616567	1,82E-06
CD81	0,36401	7,988999	3,48E-06	NSL1	-0,19229	5,088868	0,003069
UBE4A	0,373808	6,667467	8,99E-05	CARM1	-0,1963	6,826632	0,004858
POMGNT2	0,296623	4,554919	0,005245	NDUFA9	-0,42812	3,376059	0,001439
FHL3	0,576563	5,50873	1,35E-07	DNAJC2	-0,25597	5,940789	0,000162
CDKN1A	0,35324	4,367889	0,001764	CETN3	-0,39674	4,114045	0,000298
DGCR6L	0,474234	4,621093	0,000248	RNF34	-0,35616	5,258852	8,22E-05
SLC25A26	0,441826	4,350664	8,32E-05	LRP11	-0,4243	5,625555	3,97E-06
SPCS3	0,36017	7,693757	0,000239	PPP1CC	-0,30626	7,808015	2,64E-05
RC3H1	0,224127	5,826584	0,008498	BLOC1S4	-0,26588	3,808089	0,027211
MGST3	0,182386	6,498255	0,048253	C2orf69	-0,31716	5,598791	0,000895
MYCBP2	0,445331	6,283854	5,85E-05	MSL1	-0,20785	7,212002	0,001567
EPM2A	0,435777	2,271546	0,016634	EIF5B	-0,30973	8,32691	0,000314
SDF4	0,329782	7,060888	0,000156	PDCL	-0,58221	4,815539	2,08E-06
BLCAP	0,505552	5,669224	3,83E-06	MEPCE	-0,34128	5,543396	0,000173
PARP4	0,447458	7,242812	1,9E-06	RNF8	-0,30878	5,303546	0,000132
P4HB	0,294007	9,467233	0,003008	GSKIP	-0,29389	4,361629	0,004603
HECTD2	0,569032	5,02074	2,42E-06	NAP1L1	-0,33664	9,375424	8,35E-06
C1orf123	0,205433	4,782432	0,029444	HSPB1	-0,33731	7,214784	0,012547

COQ4	0,296641	5,760427	0,000768	SRSF1	-0,29713	8,449779	0,000361
ANKMY2	0,203708	5,012461	0,007858	C12orf43	-0,20255	4,277583	0,025393
ATP6AP2	0,279199	6,888492	0,0003	POLR2E	-0,28195	7,184008	0,000815
SH3D19	0,584835	5,75209	1,51E-06	BTBD2	-0,42888	6,510098	3,23E-06
CHMP1B	0,583153	5,88924	2,35E-07	REPIN1	-0,22009	6,795306	0,008835
HARS	0,347816	6,758368	2,45E-06	SNX25	-0,41064	4,185867	0,000664
ZNF276	0,218948	5,909835	0,008705	MAP7D3	-0,38698	5,134794	3,23E-05
KCTD15	0,230115	6,003186	0,028065	G2E3	-0,27466	5,815451	0,0004
NXF1	0,148256	6,985456	0,038633	DHX37	-0,22494	5,535957	0,00381
SCAMP1	0,588096	6,226224	1,31E-07	SART1	-0,25253	6,258137	0,001055
EPN1	0,260794	7,056768	0,003441	C6orf47	-0,24746	4,750987	0,01157
TCEA2	0,441902	5,53135	6,35E-05	CCDC88A	-0,45797	7,418456	1,12E-06
NCEH1	0,474226	4,564058	0,002666	ZNF281	-0,30527	5,761513	0,000305
RBM48	0,396138	4,231676	4,54E-05	AGPAT5	-0,56967	6,174258	6,73E-09
INO80D	0,358708	5,268342	0,001291	FEZ2	-0,56203	6,031014	4,04E-08
DYNC1LI2	0,229445	8,130691	0,002589	PSME4	-0,20346	7,328955	0,007493
HECTD4	0,178568	6,295326	0,039356	NUDT5	-0,41759	6,712273	1,38E-05
PLOD3	0,144267	5,720364	0,033125	SSB	-0,58769	8,098024	7,14E-09
AHI1	0,36792	5,696068	0,000589	DZIP3	-0,51966	5,159069	3,9E-05
RSAD1	0,190052	6,037168	0,009813	TMEM18	-0,24733	4,678184	0,001632
GAS8	0,238192	5,608509	0,003015	MIDN	-0,3261	6,527034	0,000237
SECISBP2L	0,321965	5,986333	0,000245	RABL6	-0,18114	7,333284	0,030404
PFKP	0,148814	7,962627	0,044393	UBE2L3	-0,30416	6,30879	0,004829
FAM149B1	0,197202	5,060908	0,036178	ICE2	-0,30336	5,962453	0,000371
CEPT1	0,565692	6,440389	4,76E-08	PDAP1	-0,50072	6,551109	3,39E-07
PHC3	0,51163	6,912786	3,64E-05	HSF2	-0,2263	4,852643	0,018422
STOML1	0,499755	2,579683	0,003765	USP16	-0,53728	6,281622	3,15E-07
AAK1	0,164128	6,949573	0,018211	ACP1	-0,1806	6,96755	0,004005
PRX	0,463198	4,170366	0,000339	MRPL50	-0,26483	5,151214	0,000673
NEK9	0,519051	6,659605	2,67E-07	SCAMP4	-0,15952	5,976752	0,025459
TXNRD3	0,350487	3,842058	0,001959	PRMT5	-0,12982	6,554647	0,040105
ZSCAN5A	0,359395	3,239978	0,02723	ATF1	-0,57526	5,174938	4,2E-06
TMEM167B	0,456503	6,913244	7,77E-06	ORC2	-0,43735	6,17356	2,9E-06
ITGB5	0,172937	6,66336	0,016074	IPO5	-0,29564	7,945565	5,05E-05
YIPF3	0,313097	6,302475	0,000183	NQO2	-0,19667	6,293213	0,001933
PI4KA	0,189158	6,953371	0,017232	PFN2	-0,31603	8,095203	3,53E-05
ARL1	0,25526	6,687683	0,000217	VPS37C	-0,2854	4,808178	0,001778
INPP5B	0,392214	5,395061	0,000632	HDDC2	-0,44241	5,214851	6,69E-05
RP11-87H9.2	0,420606	3,46784	0,002469	PCGF6	-0,17475	4,339092	0,02393
ACAD10	0,219856	4,754556	0,015218	SKIL	-0,5584	6,941287	3,78E-06
MBTPS1	0,216119	7,280116	0,001274	CSNK1G2	-0,22129	6,443899	0,026479
TAF7	0,295963	7,578703	2,73E-05	USB1	-0,4988	6,331084	3,42E-05
RNF115	0,406994	6,383157	1,09E-05	LYN	-0,45265	5,464505	3,69E-06
ANKRD28	0,303863	6,510558	0,00016	SLC25A24	-0,45851	7,373201	9,9E-08
ADORA1	0,365847	3,616266	0,006582	IPO9	-0,17491	7,653355	0,037779
CCDC127	0,29886	4,666526	0,002011	ING2	-0,26236	3,78502	0,012741
LPPR2	0,436313	5,302045	3,23E-06	CCNK	-0,33394	5,845721	0,000373
TOLLIP	0,511225	5,235189	1,36E-06	HHEX	-0,31346	3,190602	0,007716
SNX13	0,342552	5,580802	0,000379	CHSY1	-0,51253	5,597067	5,63E-07
GNPTG	0,327067	5,112839	0,000494	EBP	-0,27549	4,856527	0,003514

FAM126B	0,462173	4,58873	0,000102	RTCB	-0,18834	6,514296	0,006486
HSPA13	0,409328	6,486251	7,03E-05	TPD52L2	-0,31602	7,174212	9,41E-05
AKAP13	0,303673	6,060472	0,000682	AC092066.1	-0,44538	4,303106	0,00451
AC009948.5	0,21959	3,567488	0,039666	IDE	-0,32481	6,114412	0,000172
DECR1	0,591855	6,246612	1,19E-08	STAT5B	-0,39976	6,011408	6,65E-06
ABCB9	0,211519	3,924787	0,034212	BCOR	-0,31151	6,024147	0,000217
ZNF517	0,41373	2,286641	0,022166	LARP7	-0,34262	5,737217	0,000162
MBD6	0,398177	6,465671	0,001703	R3HDM4	-0,24257	5,566819	0,005111
CTSA	0,127867	6,822854	0,048711	STAG3L4	-0,44922	3,196677	0,002323
ACYP2	0,306668	3,001196	0,025618	PSMB5	-0,25539	6,344957	0,003869
MT-CYB	0,281535	12,50142	0,027366	THAP1	-0,32605	4,029169	0,003745
KDM3B	0,272805	7,078318	0,000159	RBM14	-0,18009	6,179099	0,011016
FAM160B1	0,264867	5,97368	0,002029	MAD1L1	-0,23954	4,705254	0,011787
LMNA	0,30368	8,71268	0,000199	RPAP3	-0,35094	6,312305	9,31E-06
PARP6	0,272164	5,81492	0,006939	HSD17B7	-0,33959	3,969507	0,000908
RERE	0,309799	7,116951	6,85E-05	PCBP1	-0,173	8,086828	0,014408
NCKIPSD	0,227948	5,153923	0,004136	FUT10	-0,29237	3,805606	0,007565
CHD9	0,289675	6,756738	0,002865	GTF3C6	-0,296	5,354119	0,000267
NDUFV1	0,208385	7,267981	0,001659	UBE2K	-0,28427	7,338815	0,000442
ATXN7	0,537298	5,857287	3,24E-06	PRR13	-0,21553	5,269575	0,002653
PRKAR1B	0,285009	4,328024	0,000719	CPSF3	-0,14328	6,387316	0,042043
MECR	0,425299	4,553369	0,000159	TUBGCP4	-0,46379	6,325305	3,04E-06
PCMTD2	0,452823	6,026284	3,14E-05	PPP2R5D	-0,33562	6,445304	8,32E-05
C17orf70	0,430674	6,431685	9,54E-07	ZNF823	-0,30232	3,818827	0,013839
PGLS	0,456742	5,069092	2,65E-05	GTF2E2	-0,2682	5,477598	0,003602
TSC22D1	0,284287	6,420218	6,27E-05	PGGT1B	-0,33813	5,134534	0,000124
MBTD1	0,188445	5,786982	0,037549	C22orf39	-0,22222	4,162243	0,038024
VGLL4	0,378174	5,995257	2,72E-05	CCT2	-0,21457	8,153767	0,000886
DNAJB14	0,404062	6,462025	4,47E-05	C12orf5	-0,16945	3,981895	0,020363
TP53I3	0,378895	4,761502	5,79E-06	PTP4A2	-0,47251	8,028939	3,06E-07
PDLIM5	0,487519	5,832282	3,08E-06	CPSF4	-0,3491	5,157145	5,89E-05
AP4B1	0,301732	5,637762	0,000154	SULF2	-0,23093	4,879703	0,015981
LEPROT	0,211441	6,223811	0,004266	CAD	-0,20087	7,955946	0,009558
OTUD7B	0,439422	6,310641	2,22E-06	IBTK	-0,21233	7,252785	0,002042
XRRA1	0,209057	4,303578	0,04281	PRPF40A	-0,41531	8,136063	1,7E-07
DOCK1	0,186084	7,316998	0,008533	ROCK2	-0,35774	7,010189	6,05E-05
MAP2K3	0,378987	5,169966	0,000134	MTHFD1L	-0,16684	6,30493	0,00955
VPS13D	0,242595	6,179594	0,007423	PTCD3	-0,23774	7,000506	0,000289
PIGQ	0,363	6,114945	0,000596	RBMS2	-0,49388	5,466093	6,85E-06
EPS15	0,24891	6,741078	0,002557	TRMT61B	-0,31295	4,511331	0,000302
AFF1	0,354541	6,294348	0,004197	PIGH	-0,43296	4,120649	3,03E-05
CORO1B	0,308304	6,440847	4,75E-05	DDX23	-0,35551	6,749546	0,000985
CERS6	0,353764	5,704145	9,21E-06	PSMC6	-0,52761	6,294391	1,2E-07
ZBTB48	0,171616	4,850148	0,032883	SRSF2	-0,25792	8,45774	0,002471
PITPNM1	0,2013	6,545408	0,002489	IMMT	-0,13903	7,470715	0,018685
SETDB1	0,3363	6,464826	5,79E-05	LYSMD4	-0,38496	2,679181	0,011863
PCYOX1L	0,341965	4,327399	0,00128	GALM	-0,25507	3,885642	0,030926
TEAD2	0,502408	4,765348	5,01E-05	WRNIP1	-0,21822	6,074963	0,000932
DDRGK1	0,307357	5,562834	0,000656	FAM161A	-0,16815	4,346771	0,026686
CXXC1	0,2915	6,488576	8,57E-05	STRADB	-0,51622	4,319754	0,000321

TCF7L2	0,392004	5,555024	0,00023	PCTP	-0,27577	4,422432	0,003178
NCKAP5L	0,355416	5,477271	0,001339	FAM104B	-0,22831	3,581642	0,047003
SRSF8	0,212226	5,249177	0,002045	RWDD1	-0,4889	5,213789	1,36E-05
GOPC	0,177597	5,985476	0,037293	PAXIP1	-0,47376	5,338176	1,71E-06
TPCN1	0,207832	6,053537	0,045628	SMG8	-0,18215	4,864726	0,024894
WDR11	0,357479	6,729817	1,12E-05	LLPH	-0,21105	4,939194	0,009338
RPL29	0,518206	7,927811	2,28E-07	DONSON	-0,16862	5,590495	0,020902
FMNL3	0,476205	5,160477	2,92E-06	GRB2	-0,20172	7,164794	0,010826
RNF103	0,387348	5,643337	9,4E-05	C12orf65	-0,20735	4,61844	0,020492
LPHN1	0,294471	7,340256	3,39E-05	C2orf47	-0,3195	4,210007	0,001065
ATP13A1	0,473718	6,672054	1,19E-06	YBX1	-0,15477	8,86318	0,04628
SLC25A38	0,421175	5,121349	2,6E-06	BIRC2	-0,41094	6,870583	9,31E-05
TTC8	0,330852	4,308585	0,017886	GIPC1	-0,34864	6,543905	0,000278
CIR1	0,206479	5,302297	0,018711	CCT8	-0,40328	8,440591	2,55E-06
ATG13	0,443942	6,259342	1,11E-06	GMFB	-0,39752	6,799788	1,26E-05
MEF2A	0,481375	6,288635	7,46E-06	SLC39A14	-0,14478	7,152362	0,028938
CRAMP1L	0,296718	4,852748	0,001559	FASTKD2	-0,41095	5,30416	6,81E-05
ERMARD	0,405125	4,871242	0,002725	WDR5	-0,24817	6,596199	0,003982
GNS	0,43527	7,526168	6,92E-07	RABEPK	-0,26305	4,915696	0,00151
ZKSCAN8	0,414747	6,61538	0,000372	NOM1	-0,17046	5,25895	0,009228
MAP4	0,161281	8,425839	0,023176	ERH	-0,34565	7,438335	0,005157
XPC	0,199523	5,964118	0,005507	PPP1R18	-0,34226	6,338653	1,14E-05
TSPYL4	0,339608	4,924488	0,00196	ANKRD9	-0,36369	4,317159	0,035929
CFAP36	0,419788	5,748153	1,68E-05	PTMAP5	-0,37604	4,479992	4,58E-05
GNPTAB	0,477886	5,893265	7,55E-06	FOXN3	-0,4626	4,723945	8,6E-05
REXO2	0,264544	5,726941	0,000753	MTA3	-0,55878	6,086355	1,01E-07
TPP1	0,180735	6,317911	0,023863	SRP68	-0,17401	7,181235	0,006289
STK10	0,244566	5,46768	0,00128	OARD1	-0,21362	4,611083	0,036164
ZZEF1	0,211745	6,276577	0,007697	TIMM23	-0,14796	6,347699	0,037254
FAM129B	0,476489	7,695199	3,81E-06	DRG1	-0,20172	5,653482	0,006253
PAPSS1	0,274234	6,723217	0,000464	FLYWCH2	-0,40834	4,613844	0,000657
TMEM260	0,293724	4,181355	0,018344	PRPF3	-0,21571	6,510431	0,00228
BAG3	0,406317	6,11148	0,000116	FTSJ3	-0,26964	6,977197	0,003114
ZNF335	0,165365	5,603116	0,01961	GTF2F2	-0,32307	5,364763	0,000182
TCEAL1	0,271852	3,974877	0,022871	FAM58A	-0,4126	4,122327	0,000911
TBC1D5	0,475573	6,595874	1,09E-06	NOP56	-0,38388	7,83058	1,3E-05
SIK3	0,319822	5,728905	0,001073	HIATL1	-0,15066	6,70288	0,045431
GAN	0,280424	5,041433	0,004718	FBXO34	-0,28128	5,029487	0,000518
KIAA1598	0,388387	6,947313	6,69E-06	ABL2	-0,20035	6,19555	0,010413
FAM118A	0,464074	5,222863	1,52E-06	ADRA2C	-0,32822	3,787101	0,007089
IGBP1	0,362739	5,518694	0,000115	HDAC2	-0,16749	7,247267	0,012241
ACADSB	0,234275	5,445378	0,003936	CEP83	-0,36338	4,997346	0,001721
ITFG3	0,30045	5,008556	0,006904	COX6C	-0,2105	6,760691	0,008259
PLEKHM1P	0,313242	4,373077	0,035274	TNIP2	-0,41305	5,27912	1,76E-05
SDR39U1	0,255228	4,851374	0,009839	SPATS2	-0,25051	6,271274	0,000472
NDUFA2	0,345777	5,133605	0,001168	PSMC1	-0,23781	5,217289	0,006615
FAM73B	0,35583	5,290255	0,000444	SMARCD2	-0,20279	6,886368	0,003553
TSC2	0,465136	6,922926	1,44E-06	CREB3	-0,20251	5,711304	0,022581
MADD	0,31322	6,285888	0,000316	ING3	-0,29407	3,113784	0,038566
BRK1	0,272807	7,017661	0,002015	NUP153	-0,2852	6,957771	0,000478

HSD17B11	0,219844	5,154342	0,00313	TSN	-0,13662	7,476254	0,033492
ZNF707	0,217744	4,15312	0,043016	DKC1	-0,12451	7,188111	0,030141
WBP1L	0,377037	4,928047	7,21E-05	GREB1L	-0,3089	3,804808	0,005349
KIAA2018	0,371107	5,288685	0,002401	NAMPT	-0,3215	6,036478	0,000123
EAPP	0,42427	5,192189	6,22E-05	NDNL2	-0,18745	4,345607	0,02169
COPA	0,155922	8,460939	0,011464	CWC27	-0,14954	5,437638	0,030148
NMRK1	0,206619	4,268036	0,045938	EIF1AD	-0,36296	5,241054	0,000242
OTUD5	0,337142	6,025402	3,74E-05	TNPO2	-0,27781	7,209398	0,00033
FAM21C	0,253633	5,395498	0,002637	WBP4	-0,47764	4,934384	2,23E-05
LINC01278	0,414828	4,999962	0,001225	PRPF6	-0,14115	6,890175	0,036721
RCAN3	0,228898	5,561349	0,005568	TMED1	-0,29115	4,181718	0,005373
TMEM115	0,310736	5,116438	0,000452	VCP	-0,24227	8,977146	0,026669
CLUHP3	0,25875	4,467218	0,021237	ANKLE2	-0,45784	6,886651	1,08E-07
APPL1	0,473032	6,871005	3,77E-07	DAPK3	-0,21337	5,474138	0,005543
EBLN3	0,358991	7,065179	1,99E-05	RP11-488L18.1	-0,22771	3,773504	0,042798
RASAL2	0,301021	5,704703	0,002501	MARK2	-0,18359	5,665911	0,025707
CETN2	0,27018	5,470693	0,012388	AP1B1	-0,19801	6,441548	0,008299
ELMSAN1	0,238418	6,136117	0,00719	PQBP1	-0,16323	5,543901	0,026706
VAMP3	0,184022	7,222856	0,007903	MOB1A	-0,34639	8,345369	4,55E-05
DICER1	0,230079	6,584877	0,01216	SLC25A4	-0,27164	5,209709	0,004684
MKL2	0,280835	5,944483	0,004485	KIAA0196	-0,1608	6,236339	0,029856
CIB1	0,410021	5,994966	0,000142	POR	-0,19133	5,854817	0,024931
RP11-147L13.	0,432879	6,205941	6,1E-06	EEF1D	-0,31139	7,827285	5,69E-05
REEP5	0,196912	7,014271	0,001959	AKAP7	-0,35753	2,959629	0,01483
ACADM	0,416024	6,018958	1,16E-06	ZNF689	-0,20795	4,277418	0,017848
COG1	0,235324	5,765038	0,003177	PTPLAD1	-0,367	7,48557	7,74E-05
FRYL	0,254008	6,232831	0,012754	LYRM4	-0,41163	4,808126	0,00028
ZNF256	0,317034	3,858749	0,010176	RUSC1	-0,19542	6,030585	0,023145
SRA1	0,284353	5,677476	0,000821	SPAST	-0,17493	5,892386	0,007641
C14orf159	0,272989	4,780655	0,014437	MRPL49	-0,29391	5,928829	0,000334
MSTO1	0,198714	4,151395	0,029487	TRAF3	-0,42453	5,715924	7,47E-06
ARHGEF40	0,120488	5,918465	0,040381	DHX16	-0,44579	6,305891	5,86E-07
C17orf62	0,299279	6,673793	0,000212	DNAAF2	-0,22309	4,212158	0,021804
NCSTN	0,161485	6,78591	0,017756	TRABD	-0,24165	5,76906	0,002032
ASCC1	0,224253	5,9069	0,001726	GLRX3	-0,34513	6,844192	9,75E-05
PDXDC1	0,184866	7,13334	0,004649	FKBP4	-0,307	6,809491	0,001108
VPS26B	0,175464	6,362107	0,004559	TGDS	-0,44932	4,029353	0,000897
TBC1D2B	0,247874	5,567493	0,004009	CCBL2	-0,46466	4,98448	9,91E-07
CDK19	0,430738	5,413461	0,000448	RALB	-0,27292	6,834741	0,000484
MLLT4	0,363979	7,374298	4,8E-05	POLR2G	-0,30715	6,503274	0,000372
TNKS1BP1	0,362307	7,457866	3,64E-05	DYNLT3	-0,23741	4,882789	0,006578
TECPR2	0,355992	4,341841	0,003372	HIRA	-0,33441	3,927303	0,003948
KDM5A	0,200053	5,85959	0,004959	METTL23	-0,36503	4,354426	0,001664
FUK	0,38067	4,84483	0,000178	CACNB1	-0,35835	4,67444	0,004389
RRAGD	0,235215	5,013864	0,015338	LEO1	-0,21672	5,208277	0,007295
ACOX1	0,425869	6,252676	9,18E-07	JOSD1	-0,14043	6,058905	0,039666
TBC1D20	0,195287	6,559951	0,018848	CCNC	-0,19627	6,122642	0,008019
RNF14	0,194385	5,823492	0,006734	ABCE1	-0,22214	7,215032	0,004018
FAM134A	0,424068	6,817195	9,74E-07	DHPS	-0,43223	5,657129	1,58E-05
UBE2G2	0,171914	7,579267	0,007305	NGDN	-0,16758	4,802278	0,033934

RNF130	0,420973	6,339613	1,93E-06	THUMPD2	-0,43249	4,546007	2,84E-06
PGBD2	0,241337	3,519681	0,026444	DPM1	-0,2733	6,162239	0,000226
ZDHHC3	0,186613	5,866902	0,016412	FUT8	-0,16734	5,405417	0,017683
SETD2	0,368902	6,251038	0,000127	ODC1	-0,14767	7,317832	0,012598
SETD5	0,163873	8,021693	0,012952	FGFR1OP2	-0,30126	5,711364	0,001469
NUDT22	0,236762	4,591264	0,023481	CDK16	-0,30394	7,573308	6,63E-05
HTRA2	0,383504	4,897614	3,36E-05	MIOS	-0,2568	4,90203	0,001987
HSPA5	0,172804	9,437527	0,007164	DNAAF5	-0,30856	5,910741	0,000135
DAB2IP	0,190351	5,935594	0,006693	ZCRB1	-0,20432	5,747185	0,010935
INPP4A	0,160816	6,127278	0,023675	NEDD8	-0,25152	5,398604	0,023125
TBC1D19	0,353692	2,783691	0,046038	KDM1B	-0,26333	5,049189	0,000468
TBC1D24	0,253944	4,983636	0,003231	KLHL13	-0,20788	4,690038	0,026503
POLR3A	0,299258	5,657154	0,000162	SLX4	-0,2337	4,678447	0,048215
MPRIP	0,352602	7,445719	1,65E-05	PMF1	-0,22694	4,071984	0,015236
CD99L2	0,332902	5,745433	0,000126	VPS37B	-0,28524	5,0183	0,001801
GNB2L1	0,349167	10,06962	8,97E-06	METTL4	-0,23262	4,806133	0,016367
RCOR3	0,316145	6,112212	0,003389	XPO6	-0,2567	7,232609	0,000283
RPS24	0,406843	9,028624	3,31E-05	GSPT1	-0,19286	7,905868	0,003514
OAT	0,161198	7,399678	0,049868	AKT1	-0,42093	7,417416	1,57E-05
ISOC2	0,267327	5,315844	0,010585	USP8	-0,33077	6,558278	4,2E-05
DCUN1D4	0,337193	6,533005	5,53E-05	MECP2	-0,36066	6,36133	9,73E-05
RHBDD2	0,279185	5,83768	0,000665	DRAM1	-0,37167	4,816333	6,8E-05
ZFYVE9	0,24849	6,03954	0,001142	DNTTIP2	-0,16265	5,966477	0,012195
RNF144A	0,175696	4,901899	0,009106	KHDRBS1	-0,19856	8,228904	0,005172
EIF2AK3	0,318371	5,282797	7,15E-05	CTNNBL1	-0,40961	5,629225	3,98E-06
CD164	0,278211	8,136628	9,88E-05	LRRC40	-0,47816	4,765049	1,2E-06
HIP1R	0,251866	6,14469	0,00187	MED6	-0,31039	4,629063	0,00027
IVD	0,315612	6,473929	0,000456	TINF2	-0,18407	4,869391	0,029141
VPS39	0,147372	6,332757	0,025872	MFAP1	-0,22306	5,521577	0,004686
IP6K1	0,251376	6,052087	0,000505	TMEM241	-0,32549	3,836855	0,001184
AHCYL1	0,16566	8,687676	0,005987	FAM35A	-0,29203	5,049944	9,21E-05
IRF2	0,218117	4,5098	0,031757	WDR89	-0,3267	4,512031	0,000412
AAR2	0,372413	5,246369	0,0004	ZNF629	-0,35009	6,025318	6,07E-05
NAPA	0,317393	5,160187	0,001158	HAUS6	-0,30356	5,671997	0,00013
AGA	0,302402	3,457372	0,043662	KIF3C	-0,17308	5,405019	0,038589
MEAF6	0,393564	6,230486	2,29E-06	ERAL1	-0,13932	5,660503	0,029246
CDC42SE1	0,137982	7,724196	0,048913	LDHB	-0,19591	9,143906	0,006358
CSGALNACT2	0,310155	5,319187	0,003655	SRBD1	-0,163	5,200617	0,048853
MBNL1	0,310399	6,803025	0,000195	PUF60	-0,14682	6,816794	0,046348
SOCS5	0,190435	5,584779	0,010183	CCAR2	-0,20329	7,565266	0,005549
CTNNA1	0,256178	9,015948	0,000143	SMPD4	-0,36233	6,77679	0,000261
GLUD1	0,283048	7,006001	7,57E-05	SLC5A3	-0,29695	6,01895	0,006677
GORASP1	0,194873	5,480901	0,017563	NRF1	-0,33291	4,527959	0,001779
LARS	0,241884	7,598805	0,003698	PITPNB	-0,22823	6,44218	0,000681
SCARB2	0,223601	7,321886	0,002299	TERF2	-0,16184	5,212752	0,041983
EXOC3	0,34224	6,104549	0,000131	GART	-0,13038	7,205446	0,027426
DNAJC3	0,17309	5,905424	0,022316	SLC25A32	-0,21958	5,303537	0,006596
ETV6	0,316438	4,676181	0,00068	VPS29	-0,31245	6,348881	0,000346
LRPAP1	0,346864	6,513251	0,000157	VKORC1L1	-0,45	5,929781	1,34E-06
CLPB	0,229221	4,413982	0,011807	ZFP36L2	-0,26828	6,168444	0,002437

ASB8	0,344227	4,66585	0,000978	MCMBP	-0,14226	6,907524	0,038948
AKT1S1	0,221325	6,47308	0,010903	HBS1L	-0,26457	5,878595	0,005058
RETSAT	0,27373	5,862007	0,000282	TMEM237	-0,40562	5,604037	7,46E-06
FGD5-AS1	0,364368	7,058542	2,92E-05	CNOT11	-0,28934	6,781568	0,000351
POLR2H	0,299411	6,316296	0,000444	VPS18	-0,30677	4,989393	0,003053
DUSP12	0,23317	5,467523	0,002514	UGP2	-0,21265	6,651283	0,00132
SERTAD1	0,272252	4,686593	0,002221	TET3	-0,21711	5,983955	0,015539
ARPIN	0,305214	5,615689	0,000212	EIF4G2	-0,24924	10,04378	0,000574
UBA6-AS1	0,280416	3,512423	0,043528	ACTN4	-0,30152	8,602554	0,000548
LINC00657	0,29006	8,644697	6,18E-05	C2orf44	-0,24576	4,015906	0,005436
IL13RA1	0,30565	6,889393	3,55E-05	NCK2	-0,28274	5,507594	0,001676
MBD1	0,195566	6,417995	0,004733	UCHL5	-0,12629	6,543094	0,029075
YIPF4	0,251744	6,414361	0,006383	PPP2R2A	-0,18162	6,583816	0,004781
LINC00909	0,339762	4,369094	0,001995	NAE1	-0,47409	6,957135	2,1E-07
PRDX6	0,335067	8,317788	1,93E-05	EMD	-0,33006	5,728404	0,000294
CTDSP1	0,230614	7,045232	0,000753	PSMA7	-0,28606	7,504712	0,001309
YIPF2	0,240758	4,644072	0,011005	SLC5A6	-0,44319	6,354711	7,64E-07
PHKA2	0,200538	5,715202	0,026082	NAA35	-0,24797	5,925031	0,003182
SETD7	0,234543	6,177338	0,004294	DCAF15	-0,28383	5,393167	0,00101
FKBP9	0,165806	6,549471	0,015566	ANP32A	-0,30193	7,377437	3,87E-05
MAPRE2	0,273052	5,550571	0,003996	UTP11L	-0,30523	5,981759	7,55E-05
ATG7	0,28405	5,217736	0,000759	PRPSAP1	-0,34412	6,074761	1,94E-05
FAM195A	0,242405	4,755182	0,007075	AKIRIN1	-0,38689	6,79145	5,04E-06
FAM21A	0,305571	5,211328	0,000972	GEMIN4	-0,30738	5,24959	0,004753
RNF169	0,248025	6,134273	0,000485	NCKAP1	-0,30226	8,509743	7,02E-05
GPBP1L1	0,20492	7,026607	0,005064	WSB2	-0,21438	6,336142	0,009014
ZMAT2	0,308869	6,296992	3,83E-05	SRSF9	-0,31504	7,390366	7,29E-05
YEATS2	0,345622	6,701539	2,41E-05	RBM17	-0,34543	7,056228	9,03E-06
MANEAL	0,230627	5,332389	0,007209	KPNA3	-0,36182	6,593307	3,88E-05
PNMA1	0,184656	6,533879	0,019825	NSMCE4A	-0,36786	5,257071	0,000117
SRI	0,302505	6,878768	3,58E-05	PTRHD1	-0,20888	4,137123	0,034212
USO1	0,178875	7,091412	0,002527	NUP62	-0,33143	6,783608	1,85E-05
XPNPEP1	0,274534	6,464716	0,000171	COQ10B	-0,18149	5,098165	0,035756
FBXO31	0,276892	5,21957	0,000471	RAP1GDS1	-0,36693	6,233371	6,4E-06
ZNF217	0,16968	7,709121	0,019293	MAFG	-0,21937	6,410255	0,003423
ARHGEF9	0,230381	4,856524	0,00566	HSPB11	-0,38738	5,681891	0,000661
NDUFS2	0,123057	7,277058	0,0332	PDS5A	-0,22154	7,778406	0,000695
DCAF8	0,259537	6,692229	0,00398	CCDC124	-0,22374	5,752468	0,047369
ZNF384	0,176096	5,794008	0,011118	CDYL	-0,17198	5,480525	0,033996
BDP1	0,210471	6,11799	0,011118	EXOSC2	-0,31126	5,908977	3,79E-05
RUFY1	0,350851	5,556775	1,02E-05	GRHPR	-0,17785	6,466668	0,009633
CALCOCO2	0,313201	6,985941	0,000146	RNF187	-0,28054	7,377757	0,001141
COX20	0,172981	5,894627	0,004002	NUFIP1	-0,16937	4,269037	0,035168
CYB5R3	0,19022	6,753018	0,027211	AKR1B1	-0,16047	7,613519	0,033399
IMPACT	0,30871	5,95677	0,000665	FIP1L1	-0,3153	6,072479	7,19E-05
ASAH1	0,319224	7,194008	1,78E-05	C7orf55-LUC7I	-0,23568	5,273839	0,01063
WDR90	0,267436	5,474008	0,015507	TWF1	-0,19607	6,615347	0,001856
GRIPAP1	0,150426	6,184915	0,036054	WASF1	-0,34935	4,991321	0,000157
WIPI2	0,261575	6,026058	0,001305	DDX55	-0,38735	5,389089	1,85E-05
ZNF37A	0,254549	6,433366	0,005651	ASNSD1	-0,28624	6,167756	0,000173

PHF3	0,181475	7,209948	0,022922	RRN3	-0,16768	5,797201	0,021309
STX6	0,311676	6,042697	4,6E-05	SMU1	-0,41275	6,269927	1,06E-06
RAB9A	0,31414	5,377145	0,000779	CBX3	-0,17922	7,875411	0,013426
UCKL1	0,252268	5,580738	0,004212	SRPR	-0,22559	6,504267	0,005983
PEX19	0,183172	5,800368	0,007064	SFT2D3	-0,23621	3,74446	0,028012
ISCU	0,221955	6,079932	0,003102	PRR14L	-0,21146	6,031103	0,023781
TOP2B	0,303827	8,129182	4,14E-05	SEH1L	-0,30492	7,139525	7,45E-05
PREPL	0,249757	7,118005	0,002536	ASCC2	-0,20042	5,447971	0,009082
YY1AP1	0,200706	5,865278	0,017756	TMX1	-0,19911	6,024457	0,007922
PPP1CA	0,166812	7,446165	0,03409	SSBP1	-0,21798	6,231901	0,003031
GAB1	0,254708	4,245543	0,029338	ZNF280B	-0,21474	4,166925	0,037906
POM121C	0,214964	5,363535	0,007305	PPTC7	-0,15247	5,777115	0,022398
DYRK1A	0,236352	6,268084	0,002549	RAB8A	-0,22891	6,164038	0,041437
AES	0,218431	7,823492	0,014941	PPP4C	-0,15754	6,518068	0,046061
PRKRIP1	0,22232	4,787752	0,015306	HNRNPL	-0,15967	7,925774	0,011212
SEC22A	0,232721	3,833989	0,047488	GADD45GIP1	-0,24598	5,366122	0,027105
DZIP1L	0,187444	4,70222	0,039399	ABCC4	-0,18702	6,11503	0,018041
PEX5	0,206954	5,807151	0,003604	SUPT20H	-0,31926	6,41083	7,16E-05
PRUNE	0,173499	5,696193	0,011292	LHFPL2	-0,25226	5,203982	0,00672
SIPA1L1	0,185135	6,036977	0,010679	KANSL3	-0,15797	6,756825	0,017662
GID8	0,294928	6,357367	7,49E-05	ISG20L2	-0,25789	6,245572	0,00032
SURF6	0,218528	5,752571	0,003139	EYA3	-0,36139	5,520771	0,00033
PDPR	0,278512	7,034532	0,000443	GTF3C2	-0,16283	6,618056	0,037364
CLCC1	0,190656	6,997698	0,007189	PHF20L1	-0,32247	5,400959	0,004461
PIEZO1	0,170233	7,505573	0,02188	HNRNPLL	-0,21301	6,001682	0,003968
B4GALT4	0,239553	5,362635	0,000946	VRK2	-0,27011	5,529803	0,000543
PPP2R4	0,221073	8,451902	0,001472	HMGXB4	-0,224	5,6679	0,000928
RAB40B	0,165372	4,676445	0,027211	HS1BP3	-0,32409	4,926406	0,00023
LAMTOR5	0,259309	7,013698	0,000371	HIBADH	-0,2837	5,624046	0,009821
RCN1	0,120347	7,488152	0,048429	PTPN14	-0,37731	7,152704	6,89E-05
SACM1L	0,17208	5,958531	0,005389	TANGO6	-0,22337	4,58685	0,020416
UBC	0,183697	9,194812	0,009332	THAP4	-0,2507	5,904335	0,00076
MRPL24	0,173064	6,01905	0,016312	SHMT1	-0,29054	6,134528	0,000679
SPTAN1	0,187933	8,606109	0,003996	NRAS	-0,28466	8,098675	0,000242
DHX30	0,217847	6,733435	0,001792	PFDN2	-0,13695	6,030533	0,03318
KIF16B	0,207808	4,643689	0,010408	AUP1	-0,2639	6,967691	0,001069
TOR1AIP2	0,15142	7,13833	0,021166	COPS3	-0,1495	6,277114	0,04124
NINJ1	0,174325	4,983312	0,025587	TDP2	-0,2731	5,828812	0,000367
38961	0,17151	5,837599	0,013248	SMYD5	-0,17781	5,736221	0,014161
SNX14	0,248901	6,23135	0,001474	SRP72	-0,21691	7,476521	0,000385
RHOA	0,19878	8,994673	0,00619	PEX14	-0,24269	4,610826	0,013303
RPL10	0,205799	8,497418	0,01149	CASP6	-0,21028	4,64971	0,01254
ZNF32	0,204936	5,395407	0,010337	PSMC2	-0,29111	6,700677	0,000733
UBXN6	0,203756	6,083515	0,035619	EIF3D	-0,1809	6,976904	0,004251
ACVR1	0,177495	5,299186	0,014137	VPS54	-0,38225	6,190484	2,03E-06
MKLN1	0,215982	6,736768	0,003912	DPY30	-0,28324	5,534867	0,000511
PUM1	0,142527	7,228052	0,015628	LAS1L	-0,16038	6,350985	0,016236
TSPAN3	0,210737	6,922692	0,007152	FKBP3	-0,23304	6,399277	0,001291
ACTR8	0,187144	5,029035	0,012952	SF3B2	-0,22748	8,173347	0,001504
C6orf89	0,13521	6,457065	0,035168	XIAP	-0,22468	6,33017	0,005756

ATP13A2	0,182934	6,218452	0,012848	SLC27A4	-0,17571	5,612346	0,039936
RARG	0,210042	6,275623	0,00112	DDA1	-0,21312	5,119565	0,011805
RPL13	0,162979	8,289563	0,034613	LAMTOR3	-0,28751	4,707981	0,003449
CD63	0,163672	8,253463	0,031918	ZDHHC7	-0,26842	6,190412	0,003359
PLA2G12A	0,174737	5,733635	0,014762	ACOT13	-0,31035	5,047324	0,000158
SH2B3	0,166389	5,367165	0,03721	NAF1	-0,29376	4,195339	0,004424
WASF2	0,17882	7,53981	0,004357	MAEA	-0,29273	6,017467	0,002332
RAB22A	0,165127	6,363371	0,012122	NPLOC4	-0,17735	8,132639	0,008726
DBN1	0,203556	7,742283	0,002904	CDC37	-0,25678	7,673563	0,001815
HM13	0,185647	7,716262	0,008842	SMARCA5	-0,16345	7,86822	0,006915
ATP2C1	0,179919	7,096964	0,003222	CSNK2A2	-0,14106	6,890442	0,036351
GSS	0,126029	6,656784	0,042827	MT-ND1	-0,24418	12,50092	0,027362
ELF2	0,14802	5,643319	0,027596	KIF21A	-0,21365	5,497098	0,003963
UBQLN2	-0,1554	5,988463	0,012583	EIF3B	-0,25517	7,670026	0,002746
SLC25A17	-0,1539	4,944614	0,030128	HPRT1	-0,26995	5,823913	0,00362
LARP4B	-0,16376	6,857821	0,010812	EWSR1	-0,27762	8,442474	6,31E-05
SPTSSA	-0,18329	5,806403	0,026669	UBN1	-0,22577	6,37531	0,003061
BCR	-0,18627	5,353483	0,008613	TCF20	-0,17171	6,12319	0,012922
ERCC6L2	-0,19871	4,926585	0,0399	NIPSNAP1	-0,37102	6,397459	1,28E-05
IER3IP1	-0,24692	5,995373	0,001763	METTL9	-0,2157	6,870849	0,00188
PWP1	-0,23017	6,414122	0,000382	CSRP2	-0,17111	4,136894	0,03825
GNL2	-0,20787	6,624944	0,002015	SF3A1	-0,16259	7,29927	0,025665
C1orf27	-0,17037	5,401176	0,024454	ARHGDIA	-0,30317	8,388755	0,004589
ZNF609	-0,15494	6,067258	0,039258	RPS23	-0,13073	8,084525	0,048118
ZFAND6	-0,13921	5,737059	0,045751	SNRNP35	-0,20818	3,807086	0,039933
TAF3	-0,17117	4,552895	0,04236	HDAC8	-0,25403	4,702243	0,006338
TAF6	-0,24096	6,077818	0,00153	SMIM20	-0,25522	4,385789	0,01089
SBNO1	-0,20042	6,988673	0,010943	TMEM167A	-0,18885	6,981451	0,006162
PPP1R9B	-0,18116	7,42843	0,006804	DTNBP1	-0,16235	5,097563	0,045108
SYAP1	-0,22552	6,696036	0,001787	SNX5	-0,13246	6,971068	0,027726
TMEM164	-0,16786	4,873955	0,0487	PRICKLE3	-0,15098	4,240156	0,043658
CUL1	-0,22275	6,543067	0,00139	UBXN2A	-0,34457	5,869851	1,09E-05
PRRG1	-0,20846	4,716453	0,006485	ANKRD54	-0,23104	5,152167	0,005405
UBE2I	-0,15476	7,067568	0,027277	CIPC	-0,29924	4,236191	0,020224
RPRD1A	-0,23806	7,12933	0,000366	SLC4A2	-0,17338	7,425844	0,008268
CCAR1	-0,14906	7,692725	0,013187	PRRC2C	-0,24037	8,440089	0,000943
ILVBL	-0,22665	5,843243	0,003172	FARSA	-0,13943	6,194928	0,034456
LYPLA1	-0,14472	6,835651	0,031651	EMC2	-0,24617	5,060761	0,003439
CKAP4	-0,14526	7,066736	0,030351	SUCLA2	-0,24174	5,668314	0,000771
FOXP4	-0,14659	6,042103	0,038604	ZDHHC18	-0,31565	5,714777	0,000369
MPDU1	-0,18117	5,125199	0,01323	DCAF13	-0,24389	6,140198	0,000497
MSRB3	-0,14323	7,021556	0,01359	ODF2	-0,12566	7,167005	0,027008
PTTG1IP	-0,20235	7,734796	0,000772	POLR2C	-0,31743	6,15833	0,000141
KCTD20	-0,18829	7,048903	0,003893	UBE3A	-0,17524	6,860621	0,024448
UQCRFS1	-0,15346	6,221878	0,025547	NOP2	-0,1927	6,217662	0,02519
USP14	-0,22507	7,683002	0,000732	DCTN6	-0,27018	4,443825	0,011048
KAT5	-0,14776	5,211046	0,047575	UBE2J2	-0,2967	5,737024	0,002299
WDR36	-0,17597	5,795592	0,021732	TAF11	-0,21808	5,329143	0,003667
ETFA	-0,16622	6,722713	0,010382	ARHGAP10	-0,18869	4,664302	0,034974
INO80	-0,16164	5,493058	0,030497	BMS1	-0,18508	7,040461	0,008732

RPAP1	-0,14475	5,65298	0,02787	NCS1	-0,27851	6,238151	0,002832
GTF3C5	-0,16589	6,148786	0,013683	PHLDB1	-0,33719	6,868647	7,49E-05
DPP3	-0,16819	5,813371	0,035736	STAMBP	-0,27617	6,283125	0,000159
PGRMC1	-0,19385	7,215232	0,008019	AGFG1	-0,17423	7,174121	0,010754
UBTD2	-0,16784	5,786759	0,033167	CDK5RAP1	-0,1992	5,438855	0,006756
AGPAT1	-0,16412	5,8358	0,022427	KDM1A	-0,33913	7,878466	3,96E-05
AGAP1	-0,17762	6,641452	0,014164	KTI12	-0,28456	3,879987	0,00425
GLYR1	-0,13541	6,714296	0,044531	NOL7	-0,23819	6,082257	0,004547
CAT	-0,13144	5,711628	0,046128	IMP3	-0,18775	4,980333	0,022487
OIP5-AS1	-0,16481	7,102502	0,015338	PYGL	-0,19565	6,617224	0,001216
PPP2R5E	-0,18642	6,375451	0,004858	EIF3M	-0,24481	7,551483	0,000289
INTS10	-0,15608	6,471859	0,015691	MAGEF1	-0,17621	5,806616	0,043615
YTHDF1	-0,14479	6,57764	0,014327	DUSP11	-0,18597	5,01733	0,011717
POLDIP3	-0,11936	6,373962	0,037549	ZNF700	-0,24674	4,923052	0,020603
SPAG9	-0,18373	7,618475	0,005565	TMEM41B	-0,30654	6,404539	9,03E-05
TRMT1L	-0,15739	5,368144	0,020922	VAPB	-0,1923	6,601661	0,003348
BRD7	-0,16489	6,304279	0,007212	SLTM	-0,27427	7,353538	6,23E-05
SDHB	-0,23116	6,528786	0,004682	GLOD4	-0,19048	6,124336	0,005869
DHX35	-0,19385	4,690715	0,038633	QKI	-0,30417	7,810383	0,000188
CDK11B	-0,21729	5,323345	0,007924	SNRPB2	-0,16717	6,529544	0,029021
CHERP	-0,167	6,29363	0,029487	FBXL12	-0,22179	4,713202	0,021324
LSM14B	-0,20202	5,968824	0,004879	INTS9	-0,20752	4,272184	0,041025
TMEM184B	-0,17772	6,152009	0,00778	RPS7	-0,22425	8,392522	0,003268
UEVLD	-0,23657	5,541024	0,004377	SLC25A22	-0,17557	5,548651	0,015491
TFCP2	-0,14898	5,919457	0,024586	PEX13	-0,25295	5,823808	0,00254
MRS2	-0,24056	6,095509	0,000739	YIPF6	-0,26033	5,922021	0,001493
LDB1	-0,16634	5,993846	0,028994	TPM4	-0,27408	8,819742	0,000535
ZKSCAN5	-0,23402	4,898515	0,002427	DEPDC5	-0,18897	3,952568	0,015974
HEATR3	-0,17998	5,140762	0,024033	TOP1	-0,14217	7,473272	0,021326
INTS2	-0,16766	5,672014	0,01987	METTL3	-0,29544	6,054642	0,000273
KLHL5	-0,20209	6,127862	0,012907	CTBP2	-0,22589	6,556722	0,001316
SUDS3	-0,23055	6,139575	0,001038	TBC1D10B	-0,27601	5,809414	0,00059
RBBP4	-0,20236	7,844636	0,007149	MESDC2	-0,19905	5,560426	0,039567
XPO4	-0,16194	6,509399	0,035921	COPS4	-0,15736	5,676912	0,017549
PRPF38A	-0,14481	6,687441	0,02011	DNAJC14	-0,19093	4,789678	0,02125
TCTN3	-0,17659	5,668058	0,014331	OCIAD1	-0,14471	7,077161	0,026686
GPS1	-0,1602	7,123009	0,018672	DDX1	-0,24995	7,595137	0,000156
				RAD23A	-0,21049	6,877709	0,001784

Supplementary Table 4. Gene enrichment analysis						
**Commonly UP-regulated genes (890 genes in comparison, 38055 in universe)**						
Gene Set Name	# Genes in Gene Set (K)	Description	# Genes in Overlap (k)	k/K	p-value	FDR q-value
HALLMARK_INTERFERON_GAMMA_RESPONSE	200	Genes up-regulated in response to IFNG [GeneID=3458].	25	0,125	1,14E-11	5,69E-10
HALLMARK_P53_PATHWAY	200	Genes involved in p53 pathways and networks.	23	0,115	4,07E-10	1,02E-08
HALLMARK_INTERFERON_ALPHA_RESPONSE	97	Genes up-regulated in response to alpha interferon proteins.	16	0,1649	9,51E-10	1,58E-08
HALLMARK_COAGULATION	138	Genes encoding components of blood coagulation system; also up-regulated in platelets.	15	0,1087	9,20E-07	1,15E-05
HALLMARK_APICAL_JUNCTION	200	Genes encoding components of apical junction complex.	17	0,085	5,46E-06	3,41E-05
HALLMARK_EPITHELIAL_MESENCHYMAL_TRANSITION	200	Genes defining epithelial-mesenchymal transition, as in wound healing, fibrosis and metastasis.	17	0,085	5,46E-06	3,41E-05
HALLMARK_INFLAMMATORY_RESPONSE	200	Genes defining inflammatory response.	17	0,085	5,46E-06	3,41E-05
HALLMARK_KRAS_SIGNALING_UP	200	Genes up-regulated by KRAS activation.	17	0,085	5,46E-06	3,41E-05
HALLMARK_ESTROGEN_RESPONSE_EARLY	200	Genes defining early response to estrogen.	16	0,08	2,18E-05	1,21E-04
HALLMARK_HYPOXIA	200	Genes up-regulated in response to low oxygen levels (hypoxia).	15	0,075	8,14E-05	4,07E-04
HALLMARK_UV_RESPONSE_DN	144	Genes down-regulated in response to ultraviolet (UV) radiation.	12	0,0833	1,54E-04	6,99E-04
HALLMARK_IL2_STAT5_SIGNALING	200	Genes up-regulated by STAT5 in response to IL2 stimulation.	14	0,07	2,84E-04	1,09E-03
HALLMARK_TNFA_SIGNALING_VIA_NFKB	200	Genes regulated by NF-kB in response to TNF [GeneID=7124].	14	0,07	2,84E-04	1,09E-03
HALLMARK_ESTROGEN_RESPONSE_LATE	200	Genes defining late response to estrogen.	13	0,065	9,25E-04	3,08E-03
HALLMARK_MYOGENESIS	200	Genes involved in development of skeletal muscle (myogenesis).	13	0,065	9,25E-04	3,08E-03
HALLMARK_IL6_JAK_STAT3_SIGNALING	87	Genes up-regulated by IL6 [GeneID=3569] via STAT3 [GeneID=6774], e.g., during acute phase response.	8	0,092	9,96E-04	3,11E-03
HALLMARK_APOPTOSIS	161	Genes mediating programmed cell death (apoptosis) by activation of caspases.	11	0,0683	1,50E-03	4,41E-03
HALLMARK_KRAS_SIGNALING_DN	200	Genes down-regulated by KRAS activation.	12	0,06	2,79E-03	7,75E-03
HALLMARK_COMPLEMENT	200	Genes encoding components of the complement system, which is part of the innate immune system.	11	0,055	7,77E-03	2,05E-02
HALLMARK_HEDGEHOG_SIGNALING	36	Genes up-regulated by activation of hedgehog signaling.	4	0,1111	9,66E-03	2,42E-02
HALLMARK_PANCREAS_BETA_CELLS	40	Genes specifically up-regulated in pancreatic beta cells.	4	0,1	1,39E-02	3,32E-02
HALLMARK_ALLOGRAFT_REJECTION	200	Genes up-regulated during transplant rejection.	10	0,05	1,99E-02	4,33E-02
HALLMARK_MTORC1_SIGNALING	200	Genes up-regulated through activation of mTORC1 complex.	10	0,05	1,99E-02	4,33E-02

**Commonly DOWN-regulated genes (1089 genes in comparison, 38055 in universe)**						
Gene Set Name	# Genes in Gene Set (K)	Description	# Genes in Overlap (k)	k/K	p-value	FDR q-value
HALLMARK_EPITHELIAL_MESENCHYMAL_TRANSITION	200	Genes defining epithelial-mesenchymal transition, as in wound healing, fibrosis and metastasis.	45	0,225	2,90E-27	1,45E-25
HALLMARK_HYPOXIA	200	Genes up-regulated in response to low oxygen levels (hypoxia).	34	0,17	6,42E-17	1,60E-15
HALLMARK_UV_RESPONSE_DN	144	Genes down-regulated in response to ultraviolet (UV) radiation.	27	0,1875	8,20E-15	1,37E-13
HALLMARK_TNFA_SIGNALING_VIA_NFKB	200	Genes regulated by NF-kB in response to TNF [GeneID=7124].	29	0,145	8,18E-13	1,02E-11
HALLMARK_COAGULATION	138	Genes encoding components of blood coagulation system; also up-regulated in platelets.	24	0,1739	1,40E-12	1,40E-11
HALLMARK_KRAS_SIGNALING_UP	200	Genes up-regulated by KRAS activation.	28	0,14	4,82E-12	4,02E-11
HALLMARK_ESTROGEN_RESPONSE_EARLY	200	Genes defining early response to estrogen.	25	0,125	7,67E-10	5,48E-09
HALLMARK_APOPTOSIS	161	Genes mediating programmed cell death (apoptosis) by activation of caspases.	21	0,1304	7,99E-09	4,99E-08
HALLMARK_ESTROGEN_RESPONSE_LATE	200	Genes defining late response to estrogen.	23	0,115	1,80E-08	1,00E-07
HALLMARK_COMPLEMENT	200	Genes encoding components of the complement system, which is part of the innate immune system.	20	0,1	1,43E-06	6,48E-06
HALLMARK_INTERFERON_GAMMA_RESPONSE	200	Genes up-regulated in response to IFNG [GeneID=3458].	20	0,1	1,43E-06	6,48E-06
HALLMARK_IL6_JAK_STAT3_SIGNALING	87	Genes up-regulated by IL6 [GeneID=3569] via STAT3 [GeneID=6774], e.g., during acute phase response.	12	0,1379	7,00E-06	2,92E-05
HALLMARK_INFLAMMATORY_RESPONSE	200	Genes defining inflammatory response.	18	0,09	2,02E-05	7,21E-05
HALLMARK_KRAS_SIGNALING_DN	200	Genes down-regulated by KRAS activation.	18	0,09	2,02E-05	7,21E-05
HALLMARK_IL2_STAT5_SIGNALING	200	Genes up-regulated by STAT5 in response to IL2 stimulation.	17	0,085	6,97E-05	2,32E-04
HALLMARK_APICAL_JUNCTION	200	Genes encoding components of apical junction complex.	16	0,08	2,26E-04	6,29E-04
HALLMARK_GLYCOLYSIS	200	Genes encoding proteins involved in glycolysis and gluconeogenesis.	16	0,08	2,26E-04	6,29E-04
HALLMARK_MYOGENESIS	200	Genes involved in development of skeletal muscle (myogenesis).	16	0,08	2,26E-04	6,29E-04
HALLMARK_ANDROGEN_RESPONSE	100	Genes defining response to androgens.	10	0,1	5,97E-04	1,57E-03
HALLMARK_ALLOGRAFT_REJECTION	200	Genes up-regulated during transplant rejection.	15	0,075	6,91E-04	1,73E-03
HALLMARK_WNT_BETA_CATENIN_SIGNALING	42	Genes up-regulated by activation of WNT signaling through accumulation of beta catenin CTNNB1 [GeneID=1499].	6	0,1429	1,18E-03	2,80E-03
HALLMARK_CHOLESTEROL_HOMEOSTASIS	74	Genes involved in cholesterol homeostasis.	8	0,1081	1,24E-03	2,83E-03
HALLMARK_APICAL_SURFACE	44	Genes encoding proteins over-represented on the apical surface of epithelial cells, e.g., important for cell polarity (apical area).	6	0,1364	1,51E-03	3,28E-03
HALLMARK_P53_PATHWAY	200	Genes involved in p53 pathways and networks.	12	0,06	1,30E-02	2,60E-02
HALLMARK_XENOBIOTIC_METABOLISM	200	Genes encoding proteins involved in processing of drugs and other xenobiotics.	12	0,06	1,30E-02	2,60E-02

HALLMARK_ANGIOGENESIS	36	Genes up-regulated during formation of blood vessels (angiogenesis).	4	0,1111	1,90E-02	3,65E-02
HALLMARK_INTERFERON_ALPHA_RESPONSE	97	Genes up-regulated in response to alpha interferon proteins.	7	0,0722	2,15E-02	3,97E-02
HALLMARK_PANCREAS_BETA_CELLS	40	Genes specifically up-regulated in pancreatic beta cells.	4	0,1	2,70E-02	4,81E-02
HALLMARK_ADIPOGENESIS	200	Genes up-regulated during adipocyte differentiation (adipogenesis).	11	0,055	2,98E-02	4,96E-02
HALLMARK_HEME_METABOLISM	200	Genes involved in metabolism of heme (a cofactor consisting of iron and porphyrin) and erythroblast differentiation.	11	0,055	2,98E-02	4,96E-02

Supplementary Table 5. IC50s of the different drugs in WT and KO cells

Drug	Cell line	IC50±SD	IC50 ratio KO/WT	p-value
DDP [µM]	A2780<br>A2780 KO	14.4 ± 0.1<br>17.8 ± 6.8	1.23	n.s.
PTX [nM]	A2780<br>A2780 KO	2.3 ± 1.3<br>1.2 ± 0.57	0.52	n.s.
OLA [µM]	A2780<br>A2780 KO	114,3 ± 54,2<br>62,2 ± 15,4	0.5	n.s.
ET743 [nM]	A2780<br>A2780 KO	11.4 ± 1.4<br>13.7 ± 2	1.2	n.s.
**VE822 [µM]**	**A2780<br>A2780 KO**	**0.39 ± 0.07<br>1.99 ± 0.81**	**5.1**	**0.027**
KU55933 [µM]	A2780<br>A2780 KO	23.8 ± 0.2<br>24.7 ± 4.6	1.03	n.s.
PF477736 [µM]	A2780<br>A2780 KO	0.58 ± 0.25<br>0.74 ± 0.17	1.2	n.s.
MK1775 [µM]	A2780<br>A2780 KO	1.2 ± 0.32<br>1.36 ± 0.64	1.13	n.s.
THZ1 [nM]	A2780<br>A2780 KO	18.4 ± 0.5<br>23.8 ± 1.5	1.29	n.s.
THZ1 HYDRO [nM]	A2780<br>A2780 KO	22.8 ± 2<br>24.1 ± 0.42	1.05	n.s.
